# A psychophysical measurement on subjective well-being and air pollution

**DOI:** 10.1038/s41467-019-13459-w

**Published:** 2019-11-29

**Authors:** Yuan Li, Dabo Guan, Yanni Yu, Stephen Westland, Daoping Wang, Jing Meng, Xuejun Wang, Kebin He, Shu Tao

**Affiliations:** 1 Institute of Blue and Green Development, Weihai Institute of Interdisciplinary Research, Shandong University, Weihai, 264209 China; 2 Institute of Resource, Environment and sustainable Development, Jinan University, Guangzhou, 510632 China; 3 Department of Earth System Science, Tsinghua University, Beijing, 100080 China; 4 School of Design, University of Leeds, Leeds, LS2 9JT UK; 5 School of Urban and Regional Science, Institute of Finance and Economics Research, Shanghai University of Finance and Economics, Shanghai, 200433 China; 6 The Bartlett School of Construction and Project Management, University College London, London, WC1E 7HB UK; 7 Laboratory for Earth Surface Processes, College of Urban and Environmental Sciences, Peking University, Beijing, 10080 China; 8 State Key Joint Laboratory of Environmental Simulation and Pollution Control, School of Environment, Tsinghua University, Beijing, 100084 China

**Keywords:** Environmental impact, Environmental social sciences

## Abstract

Although the physical effects of air pollution on humans are well documented, there may be even greater impacts on the emotional state and health. Surveys have traditionally been used to explore the impact of air pollution on people’s subjective well-being (SWB). However, the survey techniques usually take long periods to properly match the air pollution characteristics from monitoring stations to each respondent’s SWB at both disaggregated spatial and temporal levels. Here, we used air pollution data to simulate fixed-scene images and psychophysical process to examine the impact from only air pollution on SWB. Findings suggest that under the atmospheric conditions in Beijing, negative emotions occur when PM_2.5_ (particulate matter with a diameter less than 2.5 µm) increases to approximately 150 AQI (air quality index). The British observers have a stronger negative response under severe air pollution compared with Chinese observers. People from different social groups appear to have different sensitivities to SWB when air quality index exceeds approximately 200 AQI.

## Abstract (web summary)

Air pollution can affect people’s emotional status and well-being. Here, the authors simulate fixed-scene images to show that under the atmospheric conditions in Beijing, negative emotions occur when air quality index of PM_2.5_ increases to approximately 150.

## Introduction

Subjective well-being (SWB) has attracted increasing attention from researchers and policymakers in recent decades^1–4^. For example, Bhutan officially established the gross national happiness (GNH) measure to legally replace the traditional economic policy goal of increased gross domestic product (GDP)^5^. Many developed countries are likely to consider SWB during planning processes and when assessing the impacts of policy decisions. Studies show that SWB is significantly negatively related to many air pollutants^6–9^, and individuals place a higher value on the loss of an environmental feature than on gaining an equivalent feature^10,11^. Researchers using surveys usually evaluate people’s overall SWB state via interviews or questionnaires covering rich information on the idiosyncrasies of subjects. Researchers continue to make efforts to match air pollution data and happiness data in a more disaggregated way. For example, in a study by Zhang et al. in 2017, they successfully valued air quality using moment-to-moment happiness data on a daily and local level and found that bad daily air quality does not affect overall life satisfaction that much but reduces hedonic happiness and increases the rate of depressive symptoms^12^. The psychophyscial method was first used to estimate scenic beauty in the 1970s^13^ and was then widely used to establish visual air quality standards due to its robustness and effectiveness^14–17^. In 2018, Li et al. reviewed the features and limitations of previous survey studies on quantifying the effects of air pollution on SWB, and further displayed the progress of psychophysics and its application in landscape and air quality research, and then proposed using a psychophysics application to quantify air pollution impact on SWB^18^. In 2018, Yang et al. adopted a psychophysical method to collect self-reported data and the analysis method which is widely employed in a survey approach to analyze and measure the moment-to-moment emotions^19^. They found that extreme emotional experiences related to hazardous air conditions may overpower people’s memory and mislead their judgement on improved air quality. In our current research, we adopted the classic colour management technique to process and control air quality images. The Categorical Judgement Model, one of the popular methods in the visual perception field, was adopted to design experiments and collect and process psychophysical data. The psychophysical data processing method applied allowed us to closely qualify the air pollution effect on SWB and directly monitor the trends of SWB and effects as air pollution changes^18^. Wider air pollutants scales were covered and richer air pollutants were considered in our study.

This study employed psychophysical methods and experiments to investigate people’s emotion’s changes due to air pollution (see Methods). Relevent ethical procedures were approved by the Ethics and Research Committee at Shandong University (Weihai). Informed consent was obtained from every individual observer.

## Results and discussion

### General air pollution effects

We simulated a set of air pollution images of Beijing by building a model to explain the relationship between colour information from colour-managed fixed-scene digital images and collected hourly air pollution data and weather/climate data in Beijing^5^. Then, we conducted laboratory-based psychophysical visual experiments. Observers were asked to use their positive and negative emotions as a ruler to measure/judge simulated image samples exhibiting various air pollution levels^20^. Thus, the personal SWB data and air pollution data with fixed locations/scenes, times, weather and climate conditions, could be perfectly matched. The psychophysical method used for analyzing the data also automatically removes the idiosyncrasies of the observers without the need to collect personal data that have the potential to influence SWB^13^. The details of the method are provided in the supporting information (SI) and Supplementary Fig. 1.

**Fig. 1:**
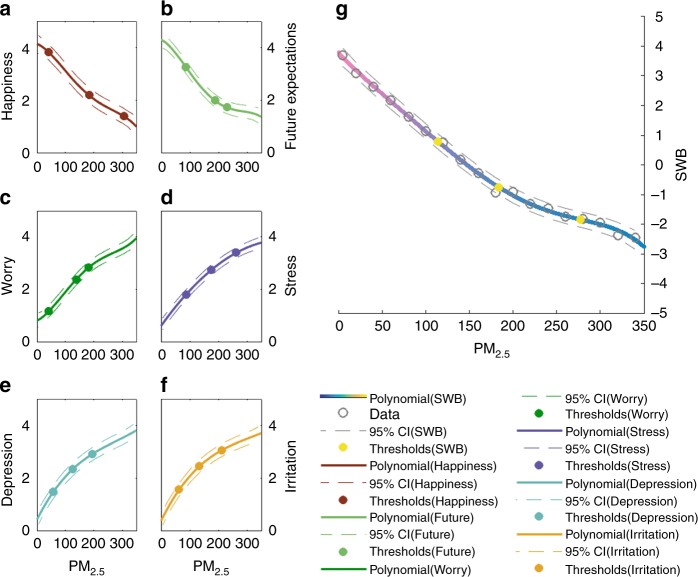
Polynomial fitting of air pollutants and perceptual data for all observers. PM_2.5_ is selected to represent the increase in air pollution combinations, including PM_2.5_, SO_2_, NO_2_, O_3_ and CO. As PM_2.5_ increases, the other pollutants increase with fixed ratios. Solid lines in all sub-figures are statistically fitted lines and dash lines are 95% confidence interval lines (CI). **a, b** The changes in the positive emotions of happiness (dark red) and expectation (light green) with changes in air pollution. **c**–**f** The changes in the negative emotions of worry (green), stress (purple), depression (sky blue) and irritation (yellow) with changes in air pollution. **g** The changes in SWB with changes in air pollution. SWB is in the perceptual range of [−5 5]. An SWB of 5 represents the most positive condition (magenta); a SWB of 0 represents a neutral condition (beige); a SWB of −5 represents the most negative condition (dark brown). See corresponding raw standardized scale value (z-score) data in Supplementary Fig. 5.

SWB is estimated by subtracting the average z-score (standardized scale values) of all negative emotions from the average of all positive emotions for each air condition^20^; thus, if SWB is greater than zero, a positive emotion dominates, and if SWB is less than zero, a negative emotion dominates. Polynomial was adopted to fit SWB and each emotion with PM_2.5_. The R-squared of all regressions is higher than 0.98. The detailed statistical indicators of polynomial fitting and coefficients for Fig. 1 are presented in Supplementary Table 2 in SI. Threshold regression was adopted to locate and estimate the numbers of thresholds. All the tested threshold regressions achieve a significant level of *p* < 0.0001. The detailed statistical indicators of threshold regressions for Fig. 1 are presented in SI from Supplementary Tables 3–9. Generally, SWB changes negatively with increasing air pollution but in different phases (see Fig. 1g and Supplementary Table 9). In first pollutant regions, where PM_2.5_ has not achieved the first threshold of 114 AQI, positive emotions are recorded, but people’s SWB scores are very sensitive (SWB falling slope = 3.71) to any change in stimuli (air pollution) until their emotions become negative when PM_2.5_ is approximately 150 AQI and SO_2_, NO_2_, O_3_ and CO simultaneously increase to 14.7, 33.8, 9.5 and 19.1, respectively. In this study, PM_2.5_ is used as a representative pollutant, and the relations between all other air pollutants and PM_2.5_ are illustrated in the SI (see Supplementary Fig. 2). In the second air pollutant region, SWB continues to decrease but at a slightly slower speed (SWB falling slope is 3.28) until PM_2.5_ increases to the second threshold 184 AQI. People’s perception of SWB is still sensitive to air pollution changes but not to the same degree as before. SWB declines much more gently as air pollution increases. When PM_2.5_ increases above approximately 184 AQI getting into the third air pollutant regions and further passing the third threshold of 277 AQI, SWB becomes stable (albeit very negative) and the SWB falling slope reduces to lower than 1.55.

Similar changes can also be found in positive emotions (Fig. 1a, b and Supplementary Tables 3, 4). Both happiness and future expectations decrease fast in the first two regions before PM_2.5_ increases to about 190 AQI (the second thresholds in both sub-figures). Then future expectations (fu-exp) and happiness steadily decline before PM_2.5_ increases to 229 AQI and 305 AQI separately. After happiness and future expectations pass the third thresholds both of them remain more stable. Interestingly, when air condition is under 80 AQI (corresponding to the first pollutant region in Fig. 1b; the first region and 1/3 of the second region in Fig. 1a), the indicator of people’s happiness tends to decline more gradually than fu-exp, according to the regional falling slopes. This result indicates that as air pollution becomes serious and tends to break people’s bottom line, people will lose future expectations quickly rather than gradually, such as losing their happiness. However, after the air pollution level exceeds 190 AQI getting into the third and fourth regions, the falling slopes of fu-exp become less, and less than the falling slopes of happiness. This result indicates that as air pollution becomes serious and breaks people’s bottom line, their fu-exp will become numb more quickly than their happiness. Negative emotions have positive relationships with increasing air pollution, and all of them show changes with air pollution that occur in three key stages (see Fig. 1c–f and Supplementary Tables 5–8). People’s worry (181 AQI), stress (175 AQI), depression (194 AQI) and irritation (213 AQI) sharply increase until PM_2.5_ exceeds around 190 AQI, after which the rising slope reduces significantly and shows negative feelings become less sensitive. People’s negative emotions tend to be more stable than before when PM_2.5_ gets into the fourth region. Interestingly, stress, depression and irritation all have relatively lower starting points than worry, and they exhibit sharper increases with lower rising slopes from zero until PM_2.5_ increases to nearly 50 AQI. This finding suggests that people’s stress, depression and irritation are more sensitive to air pollution than worry at very low levels of air pollution, and people start to worry about air pollution as the air quality reaches higher levels (although they already feel more stress, depression and irritation).

### Effects from social factors

**Fig. 2:**
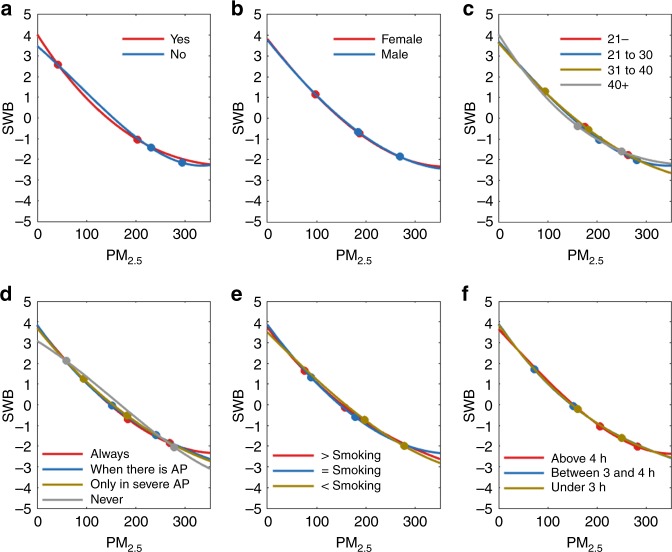
Polynomial fitting of air pollutants and subjective well-being in social groups. **a** The effect of whether or not people have children on their SWB under air pollution (red refers to yes, and blue refers to no). **b** Gender has little effect on people’s SWB (red refers to female and blue refers to male). **c** The effect of age group on SWB (red refers to people younger than 21; blue refers to people between 21 and 30; brown refers to people between 31 and 40; grey refers to people older than 40). **d** The effects of attitude towards the necessity of wearing a mask on SWB (red refers to people who always wear masks; blue refers to people who wear masks when there is air pollution; brown refers to people who wear masks only during severe air pollution; grey refers to people who never wear masks when going outside). **e** The effect of knowledge of the harmfulness of air pollution on SWB (red refers to people who think the harmfulness of air pollution is more serious than smoking; blue refers to people who think the harmfulness of air pollution is similar to smoking; brown refers to people who think the harmfulness of air pollution is less serious than smoking; grey refers to people who think air pollution is harmless and causes only discomfort). **f** The effect of the average daily exposure time to outdoor air on SWB (red refers to people who stay outdoors less than 3 h each day; blue refers to people who stay outdoors between 3 and 4 h each day; brown refers to people who stay outdoors more than 4 h each day). See raw z-score data in Supplementary Fig. 6.

Social factors that may affect the subjective responses of people to air pollution were investigated, including whether they were parents, their gender, their age group, their attitude towards the necessity of wearing a mask outdoors, their knowledge of the harmfulness of air pollution, and their outdoor exposure time. Polynomial regression and Threshold regression were adopted to analyze raw z-score data. The R-squared of all polynomial regressions is higher than 0.98 and all the tested threshold regression achieve a significant level of *p* < 0.0001. The detailed statistical indicators for Fig. 2 are presented in SI from Supplementary Tables 10–33. It was found that whether people are parents or not influenced their SWB response to different air pollutants. Figure 2a and Supplementary Tables 10–12 show that people with children (red) had higher SWB z-scores when PM_2.5_ is lower than 42 AQI (first threshold of red line). Prior to air pollution level increases to around 40 AQI, the decline slope of SWB for people who have children is twice faster than the value of people who do not have children (−0.05 versus −0.02). Moreover, the SWB scores of parents decline more rapidly as air pollution increases than those of people without children when air pollution is between 42 and 230 AQI (first threshold of blue line). Compared with the people who have children, the SWB of people without children appears to remain much steadier during the decline in air quality. Furthermore, when air pollution becomes truly hazardous (PM_2.5_ > 230 AQI), the SWB of parents tends to remain stable and higher than that of people without children.

**Fig. 3:**
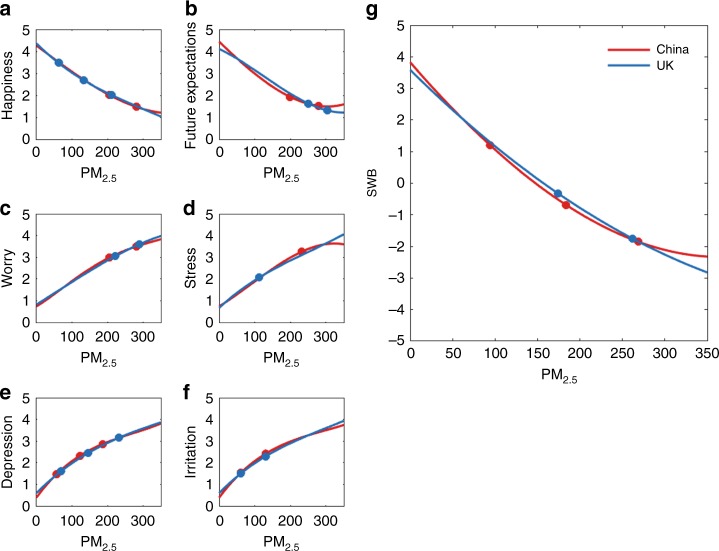
Polynomial fitting of air pollutants and perceptual data in country backgrounds. **a, b** The change in the positive emotions of happiness and future expectations with changes in air pollution. **c**, **d**–**f** The changes in the negative emotions of worry, stress, depression and irritation with changes in air pollution. **g** The changes in SWB with changes in air pollution. Positive and negative emotions and SWB are compared between observers living in China and the UK. The red line indicates that the observers in China have stronger emotions than those in the UK under the given air pollution condition (PM_2.5_); the blue line indicates that the observers in the UK have stronger emotions than those in China under the given air pollution condition (PM_2.5_).

After air pollution level breaks 230 AQI, the decline slope of SWB are −0.0068 and −0.0113, respectively, for people with and without children. This finding suggests that people with children are more sensitive and emotional to air quality changes and that they also become numb to very hazardous air quality earlier than people without children. Figure 2b and Supplementary Tables 13–15 shows the comparison of the standardized z-scores of SWB for males (blue) and females (red). Surprisingly, the finding shows that females are slightly happier at very low air pollution levels and numb slightly earlier to hazardous air conditions than the males. Figure 2c and Supplementary Tables 16–20 show the SWB responses to air pollution for four age groups. The grey line is higher than the other lines when the air pollution values are very low (PM_2.5_ is roughly below 40 AQI), and then it sharply declines as air pollution increases to about 205 AQI (the first threshold of blue line). After that, when air pollution achieves a hazardous level, the grey line stays above all the other lines. This finding indicates that the SWB of older people (between the ages of 41 and 56 in this study; shown in grey) is found to be higher under good air conditions than that of younger people (red, blue and brown). Moreover, the SWB of older people (grey) declines faster than that of younger people as air pollution deteriorates from about 40 to 205 AQI. However, the SWB of older people (grey) remains stable after PM_2.5_ increases to 250 AQI (the second threshold of grey line). This result suggests that older people care more about air quality and health than younger people and also become numb to very hazardous air quality earlier than younger people. Figure 2d and Supplementary Tables 21–25 show the SWB comparisons among people with different attitudes towards wearing masks. This figure indicates that when the air condition is very good under 59 AQI (the first threshold of grey line), the red and blue lines are higher than the brown and grey lines, and then as the air condition deteriorates to about 250 AQI around the second thresholds of red, blue and grey lines, the red and blue lines drop more sharply than the brown and grey ones. However, after that, when the air condition reaches a hazardous level, the red and blue lines remain above the brown and grey lines. When air quality is good and hazardous, the people who always wear masks (all Chinese observers have this choice) and wear masks during periods with air pollution (red, blue and brown), have higher SWB than people who never wear masks (grey). This result indicates that the more carefully people tend to protect themselves, the higher SWB they will have in good air conditions and they will become numb earlier in hazardous air conditions. However, during the deterioration of air pollution from moderate about 60 AQI to very unhealthy conditions about 250 AQI, the SWB of people that more carefully protect themselves (red, blue and brown) declines more rapidly than that of people who never wear masks (grey). In this attitude question about the necessity of wearing mask, all the Chinese observers selected Always to wearing a mask. Figure 2e and Supplementary Tables 26–29 show the SWB comparisons among people with different beliefs about the harmfulness of air pollution. When the air condition is very good with PM_2.5_ lower than about 50 AQI, the red and blue lines are higher than the brown one. However, when the air condition reaches a hazardous level with PM_2.5_ above 277 AQI (the second threshold of brown line), the red and blue lines remain above the brown line. People who believe that air pollution is more harmful (red) than or similar (blue) to smoking have higher z-scores of SWB than people that believe air pollution is less harmful (green) at the beginning and end of the air pollution scale. However, in between the good and hazardous air conditions with PM_2.5_ from around 50 to 280 AQI, people who believe air pollution is more harmful than smoking have lower SWB z-scores. Again, people’s knowledge of the general harmfulness of air pollution also proves that the more harmful people believe air pollution is, the more sensitive they are to the variation in air quality. A similar tendency is shown in Fig. 3f, which indicates the effect of outdoor exposure time on SWB judgement. Figure 2f and Supplementary Tables 30–33 show that the brown line (under 3 h outside per day) and blue line (between 3 and 4 h per day) is higher than and red (more than 4 h per day) line at the beginning and also the end of the PM_2.5_ scale. This result indicates that the shorter people stay outdoors, the higher z-scores of SWB they have during good and hazardous air conditions. This result could be because the shorter time people are able to spend outdoors, the more people are sensitive to air quality changes.

### Effects from national backgrounds

Long-term living in an environment with serious air pollution can affect people’s SWB and emotions. All of the Chinese observers lived in Beijing for at least 2 years and experienced serious air pollution, especially during the cold seasons. Their raw z-score data were compared with data for observers living in the UK in Supplementary Fig. 7. In Fig. 3, polynomial fittings of air pollutants and perceptual data (SWB and emotions) are plotted in China and UK backgrounds. R-squared of all the fittings is above 0.9690. Threshold regression was adopted to estimate the locations and the significance of thresholds. All the statistical indicators are presented in SI from Supplementary Tables 34–52. Clearly, under good air conditions where PM_2.5_ is under about 50 AQI, the observers in China (red) had obviously higher z-scores and stronger positive responses than UK observers (blue) regarding future expectations and SWB (see Fig. 3b, g). In Fig. 3g, before air pollution increases to around 100 AQI, the decline slopes of SWB are −0.0279 and −0.0224, respectively, for Chinese and British. As PM_2.5_ increased to about 270 AQI the second threshold of blue line in Fig. 3g, the z-scores of future expectations and SWB for the Chinese observers decreased faster (with falling slopes of 2.84 and 5.54) than those of the observers living in the UK (with falling slopes of 2.60 and 5.32). In contrast, the z-scores of future expectations and SWB for the observers living in the UK declined much more smoothly as the air pollution conditions deteriorated. These relatively strong mood swings could result from the frustration of long-term living in environments with air pollution and the rejoicing for blue sky conditions. Not surprisingly, when PM_2.5_ increases and exceeds the thresholds (around 290 AQI in Fig. 3b and 270 AQI in Fig. 3g), Chinese observers show a much more stable response for indicators of both future expectations and SWB. For example, after air pollution breaks around 270 AQI, the decline slope of SWB are −0.0057 and −0.0122, respectively, for Chinese and British. Figure 2b, g show that for Chinese observers the increase in PM_2.5_ from approximately 180 to 350 AQI reduces the z-scores of future expectations by 0.51 units and those of SWB by 1.70 units. Comparatively, a similar extent of PM_2.5_ increase from 0 to 180 AQI reduces future expectations by 2.29 units and SWB by 4.29 units. The responses of the UK observers also reflect this unusually stable response shown by the Chinese observers to serious air pollution, as the responses of the observers in the UK continued to decrease gradually until 270 AQI the last threshold of blue line in Fig. 3g, and a large jump was found for the worst air conditions shown in this study. This result indicates that long-term living in an environment with serious air pollution can increase the tolerance level to hazardous air pollution and make people numb to it. These trends were confirmed by the negative emotions for both groups of observers living in China and the UK (see Fig. 3c–f). Chinese observers tended to give a lower score for almost all negative emotions when the sky was blue, which indicates they have more positive emotions than people living in the UK when sunshine and blue sky are available. Additionally, when the air pollution becomes very unhealthy or hazardous (for example, PM_2.5_ above 300 AQI), UK observers usually gave a higher score than Chinese observers to negative emotions. This finding suggests that the emotions of the observers living in the UK are more sensitive under conditions of severe air pollution, and these observers have a stronger negative mood response.

We conducted a comparative analysis between Chinese and British observers in aspects of age, gender, outdoor time spending and the knowledge of the harmfulness of air pollution. Polynomial regression is adopted to fit SWB and air pollution data in Supplementary Figs. 8–11. The country comparison focusing on age indicates that the SWB of people who have experienced serious air pollution, such as Chinese citizens, is more sensitive to air condition changes and among the experienced people the older ones are even more sensitive than the younger ones. For those who have no experience of serious air pollution, like the British, the factor of age does not make too much difference on SWB under different air conditions (Supplementary Fig. 8). It can be concluded, based on the country comparison focusing on gender, that in China under frequent serious air pollution, gender plays a little role in the effect of SWB under different air pollution conditions. However, in the UK the overall air quality is much higher than China, females show more sensitivity than males in SWB with air quality changes (Supplementary Fig. 9). Based on the country comparison focusing on outdoor time spending, it is found that in serious air pollution areas like China those people who have less opportunities to spend time outside care more about the air quality when they are out. They are eager to enjoy the limited outdoor time they have each day. However, British people who spend longer outdoor time daily are more sensitive in SWB to air pollution than those who are spending 2 h or less each day. This could be due to the fact that people living in the UK have been used to excellent air quality. It is very difficult for people to accept spending lots of time outdoors in air pollution conditions (Supplementary Fig. 10). The country comparison on the knowledge of the harmfulness of air pollution indicates that when people do not believe air pollution affects health, the experience of living in serious air pollution—which Chinese have—is displayed more strongly. For those British with the knowledge that air pollution is more harmful than—or similar to—smoking, the gap between the two countries is shrinking (Supplementary Fig. 11).

### Policy implications

The impact of long-term (approximately 2 months) air pollution on SWB is quantified by the visual psychophysical method. This research identifies the thresholds of air pollution impact to mental health and demonstrate a new method that could replace or augment traditional surveys to enhance our knowledge of the impact of air pollution on people’s subjective experience and well-being. A boundary of 150 AQI is found where people’s SWB is approximately 0 and becomes negative. After 184 AQI (the second threshold in Fig. 1g), people start to become numb to air pollution until approximately 277 AQI (the third threshold in Fig. 1g). Some social groups may reflect thresholds more strongly, such as people with experience of living in serious air pollution, people with children, older people, people who care about their health, and people who spend more time outdoors. We suggest that special air hazard measures could be considered during the governance process to treat sensitive groups to build a society with better SWB. Some special figures in AQI (150, 184 and 277) should be taken into consideration during policy development.

## Method

### Experimental design

Two psychophysical experiments were conducted in China and the UK. Observers made judgements on six emotions in response to air pollution image samples under standard laboratory viewing conditions. The six emotions were happiness, expectations for the future, worry, stress, depression and irritation. The workflow, including the preparation of experimental samples, characterization of the monitor, the visual experiments and analyses, is shown in Supplementary Fig. 1.

In both visual experiments, a total of 18 different air pollution image samples were considered. These samples were simulated images intended to differ in only air pollution levels with identical weather/climate conditions, such as humidity, wind direction, wind speed, temperature, sea level (SL) pressure, cloud cover and sun angle (controlled by the number of minutes after sunrise). Therefore, in each simulated image, the weather/climate conditions were the same, but the air pollutants, including PM_2.5_, SO_2_, CO, O_3_ and NO_2,_ were different. This goal is achieved by the following steps.

### Colour information and air quality model building

First, to obtain the targeted image samples for the experiments, a model was constructed to explain the relationship between the colour information (the RGB values) from air pollution photos and the corresponding pollutant and weather/climate data of the photos. A total of 180 photos were taken by Sigma DP3 Merril in sunlight mode and the photos were saved in raw format so that the real sky colour information could be accurately recorded. The reason of using the fixed scene is to exclude the effects from scene beauty difference on people’s emotions so that the only difference among simulated images is air conditions. Therefore, a model between colour information of each photo and its corresponding air condition can be built. To match the colour information in each photo, hourly air pollutant data of PM_2.5_, SO_2_, CO, O_3_ and NO_2_ were obtained from the US embassy in Beijing, and weather/climate data were obtained every 3 h from the World Meteorological Organization (see supporting data 1). The **PM**_2.5_ varied from 17 to 399, ozone varied from 2 to 32, **NO**_2_ varied from 5 to 38, **SO**_2_ varied from 2 to 50, and **CO** varied from 2 to 60. By taking all pollutants and climate factors into consideration, the colour-AQI model **M1** gives best statistical relationship. The colour changing in image simulation is mainly driven by coloured air pollution, for example PM_2.5_. Equation (1) was used to obtain the colour-AQI model **M1** of the coefficients between photo colour information **I**_RGB_ and air pollution and weather/climate data. The adjusted R^2^ between the colour information (RGB values) of the simulated images and original photos was 0.83.

1 $${\mathbf{M}}1 = \frac{{{\mathbf{I}}_{{\mathbf{RGB}}}}}{{\left[ {\begin{array}{*{20}{c}} {{\mathbf{Ti}}} & {{\mathbf{PM}}_{2.5}} & {{\mathbf{O}}_3} \end{array}\begin{array}{*{20}{c}} {{\mathbf{CO}}} & {{\mathbf{NO}}_2} & {{\mathbf{SO}}_2\begin{array}{*{20}{c}} {{\mathbf{Te}}} & {{\mathbf{WS}}} & {{\mathbf{Hum}}}\end{array}\begin{array}{*{20}{c}}&{{\mathbf{Pre}}} & {\cos \left( {{\mathbf{WD}} \times {\mathbf{\pi }}/180} \right)\begin{array}{*{20}{c}} {{\mathbf{Cl}}} & {\mathbf{C}} \end{array}} \end{array}} \end{array}} \right]}}$$

where **I**_RGB_ is a 111 by *n* matrix, which refers to the RGB values of all pixels of each of the 111 photos

**M1** is a 13 by *n* matrix, which refers to all 12 variable coefficients and one constant;

**Ti** refers to the number of minutes since sunrise on the day the photo was shot to the shooting time;

**Te** refers to the temperature at the hour the photo was shot;

**WS** refers to the wind speed at the hour the photo was shot;

**Hum** refers to the relative humidity at the hour the photo was shot;

**Pre** refers to the SL pressure of the location at the hour the photo was shot;

**WD** refers to the wind direction at the hour the photo was shot;

**Cl** refers to the cloud cover at the hour the photo was shot;

**PM**_2.5_, **O**_3_, **CO**, **NO**_2_ and **SO**_2_ refer to the air pollutant levels in AQI;

**C** is a constant.

### Image samples production

Second, to generate a new set of air pollution images for the experiments, all air pollutant and weather/climate variables were estimated. Fixed values of the weather/climate variables **Ti**, **Te**, **WS**, **Hum**, **Pre**, **WD** and **Cl** were given for all simulated air quality images, and they were 140, −1, 4, 44, 1026, 204 and 0.5, respectively. Interestingly, the air pollutant data of SO_2_, CO, O_3_ and NO_2_ were all found to be related to PM_2.5_, so they were not fixed values but changed with PM_2.5_ values when producing the simulated images. A power model was used to predict O_3_ and NO_2_ from PM_2.5_, with R values of 0.74 and 0.86. The first-order polynomial was used to predict SO_2_ and CO, with R values of 0.71 and 0.86, as shown in Supplementary Fig. 2. With the fixed weather/climate values and the models between the other air pollutants and PM_2.5_, a set of predicted air quality images with various PM_2.5_ and other air pollutant values (AQI of PM_2.5_ ranged from 5 to 340) were generated, as shown in Supplementary Fig. 3. To simplify the explanation, PM_2.5_ was used as the key air pollutant indicator in the discussion of the main text. However, this does not mean that the other air pollutants remained the same when PM_2.5_ changed.

For both experiments, the same physical display unit model was used to collect the visual data, which were characterized using standard colour science methods^21^. The RGB values were therefore adjusted to account for the characteristics (and settings) of the monitors used in the experiments to enable accurate colorimetric display. Monitor characterizations were conducted for each display in the UK and Beijing, which were both based on 36 colour samples with 18 neutral samples, 3 pure colours (red, green and blue) and 15 samples of other colours. The channel independence of both displays was estimated, and their CIELAB colour differences ΔE were 1.23 (in the UK laboratory) and 1.68 (in the Beijing laboratory) units with black correction. A characterization model was used, and the parameters for this model were determined for both displays. In total, 15 colour samples were selected from the original sample set to test the performance of both display characterization models. Thus, the CIELAB ΔE can be obtained from the original XYZ values and the new measurements after image processing by the characterization models. The average CIELAB ΔE between the two measurements for the 15 samples was 0.58 for the display in the UK laboratory and 0.76 for the display in the Beijing laboratory. Thus, all simulated images were adjusted based on the colour characterization models before the experiments.

### Psychophyscial experiments

Two psychophysical experiments were conducted using two groups of observers in Beijing and the UK in a dark room with constant lighting conditions. The traditional Categorical Judgement was used to collect data during both experiments. Categorical Judgement is a widely used method for collecting and analyzing data in modern psychophysical research^22,23^. It is developed from Thurstone’s law of comparative judgement^24^. This theory aims to locate a given series of stimuli with different amounts of some attribute to which observers can have different response on a psychophysical continuum. In total, 18 images of different air quality conditions were examined, and 6 emotions were evaluated. During the experiment, one image (24 cm × 16 cm) was presented at a time in the centre of the display (see Supplementary Fig. 4), and the image was viewed from approximately 80 cm. Experimental simulated images are presented to each observer by random order. The observers were presented with 7 buttons underneath each image that they could click to select one of the 6 emotions. Then, the air quality image disappeared and was replaced by a new image. This process continued until all 6 emotions were judged. Before the experiment started, some basic information on the observers was collected, including gender, age and attitudes regarding the necessity to wear a mask and the impact of air pollution on health, the average number of hours spent outside and whether they had children. Then, the observers were asked to imagine that in the next 5–10 years, they would live for approximately 2 months in the air quality conditions shown on the display. The 6 emotion questions are Please select the level of happiness you receive from the 7 levels (in each case 1 refers to the lowest level, and 7 refers to the highest level); What are your expectations for the future?; Please select the level of worry you feel from the 7 levels; Please select the level of stress you feel from the 7 levels; Please select the level of depression you feel from the 7 levels; Please select the level of irritation you feel from the 7 levels. A total of 79 observers, 38 from the UK and 41 from China, took part in the study. Ten of the UK observers were originally from China, and the rest were mostly from Europe. The observers were university students or staff consisting of 31 males and 48 females, and their ages ranged from 18 to 56. All of the observers passed the Ishihara colour vision test^25^, and there were no time restrictions. Each observer made 108 (18 images × 6 questions) observations; thus, there were 8532 observations (18 × 6 × 79) in both visual experiments.

### Data analysis

The classic method of Torgerson’s law of categorical judgement was used to estimate the interval scale values from the raw psychophysical data^22,26^, as shown in Eq. (2). This processing data procedure automatically removes the idiosyncrasies of observers resulting from the use of their own standards to judge the experimental images. Using this procedure, the interval scale value *s* of each simulated image for a given effect was estimated. However, the ranges of the scale values for each effect differed, so that they could not be combined for further calculations. Equation (3) was applied to standardize the scale value *s* for all effects^27^. Thus, all the perceptual parameters *Z* for different emotions were approximately 2.5 with limits of 0 to 5. SWB researchers usually use 3 kinds of measures, life evaluation (a reflective assessment on a person’s life), effect measures (a person’s feelings or emotional states, typically measured with reference to a particular point in time)^20^ and eudaimonic measures (a sense of meaning and purpose in life)^20,28^. Here, SWB is measured based on the Kahneman model (DRM)^29^. Using his model, the net effects of each selected activity in a day are estimated. The overall SWB of the subject can be calculated by considering the duration of each activity. The net effect of each activity is the average of the positive effects minus the average of the negative effects. Multiple items are used to reduce the impact of question ambiguity or conceptual fuzziness^28^. The aim of our research is not to assess the national SWB as empirical research but to directly assess the response to air pollution. Thus, in our case, the effect on SWB from viewing air pollution images could be measured as the net effect of the average of all positive effects (expectations for the future, happiness) minus the average of all negative effects (stressed, depressed, worry, irritation). The SWB values based on positive and negative effects caused by air quality were estimated by using the averaged z-scores *Z* of positive emotions (happiness and expectation) minus the average z-scores *Z* of negative emotions (worry, stress, depression and irritation) for each simulated image. The z-scores of all emotions and SWB are shown in Supplementary Table 1.

2 $${\boldsymbol{t}}_{{\boldsymbol{gf}}} - {\boldsymbol{s}}_{{\boldsymbol{jf}}} = {\boldsymbol{x}}_{{\boldsymbol{jgf}}}\left( {{\boldsymbol{\sigma }}_{{\boldsymbol{jf}}}^2 + {\boldsymbol{\sigma }}_{{\boldsymbol{gf}}}^2 - 2{\boldsymbol{r}}_{{\boldsymbol{jgf}}}{\boldsymbol{\sigma }}_{{\boldsymbol{jf}}}{\boldsymbol{\sigma }}_{{\boldsymbol{gf}}}} \right)^{1/2}$$

where ***t***_*g*_ is the mean location of the ***g***th category boundary on the perceptual continuum emotion ***f***;

***s***_*j*_ is the scale value of simulated image ***j***;

***σ***_*g*_ is the standard deviation of the ***g***th category boundary;

***σ***_*j*_ is the standard deviation of simulated image ***j***;

***r***_*jg*_ is the correlation coefficient between the momentary positions of simulated image ***j*** and category boundary ***g***;

***x***_*jg*_ is the unit accounted for in the normal deviate corresponding to the proportion of times simulated image ***j*** is sorted below boundary ***g***.

3 $${\boldsymbol{Z}}_{{\boldsymbol{sjf}}} = \frac{{{\boldsymbol{s}}_{{\boldsymbol{jf}}} - {\bar{\boldsymbol{s}}}_{\boldsymbol{f}}}}{{{\boldsymbol{\sigma }}_{\boldsymbol{f}}}} + 5$$

where ***Z***_*sjf*_ is the standardized z-score of scale value ***s*** of the simulated image ***j*** of the emotion ***f***;

***s***_*jf*_ is the scale value of the simulated image ***j*** of emotion ***f***;

${\bar{\boldsymbol{s}}}_{\boldsymbol{f}}$ is the mean of variable ***s*** of the emotion ***f***;

***σ***_*g*_ is the standard deviation of variable ***s*** of the emotion ***f***.

### Reporting summary

Further information on research design is available in the Nature Research Reporting Summary linked to this article.

## Supplementary information

Supplementary Information

Reporting Summary

## Data Availability

All data and matlab codes are deposited at our data publishing website – China Emission Accounts and Datasets (http://www.ceads.net/wp-content/uploads/delightful-downloads/2019/10/data-link.zip). Those data can be also obtained from the corresponding author on reasonable request. Polynomial regression has been adopted to fit PM_2.5_ and Categorical Judgement Model processed perception data including all emotions and SWB. Cross-validation analysis is applied to help to decide the orders of polynomials in each fitting. Threshold regression analysis is adopted to test the number, position and significance of thresholds in each curve. All the statistical indicators and plots of raw z-score data are presented in the SI, Supporting Results session.

## References

[1] Danner D, Snowdon D, Friesen W (2001). Positive emotions in early life and longevity: findings from the Nun Study. J. Pers. Soc. Psychol..

[2] Ostir GV, Markides KS, Peek MK, Goodwin JS (2001). The association between emotional well-being and the incidence of stroke in older adults. Psychosom. Med..

[3] Cohen S, Doyle WJ, Turner RB, Alper CM, Skoner DP (2003). Emotional style and susceptibility to the common cold. Psychosom. Med..

[4] Steptoe A, Wardle J, Marmot M (2005). Positive affect and health-related neuroendocrine, cardiovascular, and inflammatory processes. Proc. Natl Acad. Sci. U. S. A..

[5] Schmitt M (2013). Subjective well-being and air quality in Germany. Schmollers Jahrbuch.

[6] Welsch H (2006). Environment and happiness: valuation of air pollution using life satisfaction data. Ecol. Econ..

[7] Smyth R, Mishra V, Qian X (2008). The environment and well-being in urban China. Ecol. Econ..

[8] MacKerron G, Mourato S (2009). Life satisfaction and air quality in London. Ecol. Econ..

[9] Zhang X, Zhang X, Chen X (2017). Valuing air quality using happiness data: the case of China. Ecol. Econ..

[10] Brown T, Gregory R (1999). Why the WTA-WTP disparity matters. Ecol. Econ..

[11] Welsch H (2002). Preferences over prosperity and pollution: environmental valuation based on happiness surveys. Kyklos.

[12] Zhang X, Zhang X, Chen X (2017). Happiness in the air: How does a dirty sky affect mental health and subjective well-being?. J. Environ. Econom. Manage.ment.

[13] Daniel, T. C. & Boster, R. S. *Measuring Landscape Esthetics: the Scenic Beauty Estimation Method* (USDA Forest Service, U.S. department of agriculture, 1976).

[14] Ely, D. W., Leary, J. T., Stewart, T. R. & Ross, D. M. in *84th Annual Meeting & Exhibition* (Air & Waste Management Association).

[15] Pryor SC (1996). Assessing public perception of visibility for standard setting exercises. Atmos. Environ..

[16] Fajardo OA, Jiang J, Hao J (2013). Assessing young people’s preferences in urban visibility in Beijing. Aerosol Air Qual. Res..

[17] Smith AE (2013). An evaluation of the robustness of the visual air quality “preference study” method. J. Air Waste Manage. Assoc..

[18] Li Y, Guan D, Tao S, Wang X, He K (2018). Review of air pollution impact on subjective well-being: survey versus visual psychophysics. J. Clean. Prod..

[19] Yang J (2018). Biased perception misguided by affect: How does emotional experience lead to incorrect judgments about environmental quality?. Global Environ. Change.

[20] Diener E (1984). Subjective Well-Being. Psychol. Bull..

[21] Berns, R. S. & Katoh, N. in *Colour Engineering* (eds Green, P. & MacDonald, L. W.) (John Wiley & Sons, 2002).

[22] Engeldrum, P. G. *Psychometric Scaling a Toolkit Imaging Systems Development* 1st edn (Imcotek Press, 2000).

[23] Liu H, Huang M, Cui G, Luo MR, Melgosa M (2013). Color-difference evaluation for digital images using a categorical judgment method. J. Opt. Soc. Am..

[24] Thurstone LL (1927). A law of comparative judgement. Psychol. Rev..

[25] Ishihara, S. *Test for Color-blindness*. 38 Plates Edition edn (Kanehara Trading INC, 2014).

[26] Torgerson, W. S. *Theory and Methods of Scaling* (John Wiley, 1958).

[27] Latimer, D. A. & Hogo, H. The effects of a tmospheric optical conditions on perceived scenic beauty. *Atmos. Environ*. **15**, 1865–1874 (1981).

[28] OECD. *OECD Guidelines on Measuring Subjective Well-being* (OECD, 2013). 24600748

[29] Kahneman D, Kreuger AB, Schkade D, Schwartz N, Stone A (2004). Towards national well-being accounts. Am. Econ. Rev..

