# Supplementary material for: A psychophysical measurement on subjective well-being and air pollution
Source: Nat Commun. 2019 Nov 29;10:5473. doi: 10.1038/s41467-019-13459-w (PMC6884631; doi:10.1038/s41467-019-13459-w)
Supplement: Supplementary file 1 — Supplementary Information [file 41467_2019_13459_MOESM1_ESM.docx]

**Supplementary Information for**

**A psychophysical measurement on subjective well-being and air pollution**

**by Li et al.**

# Supplementary Figures


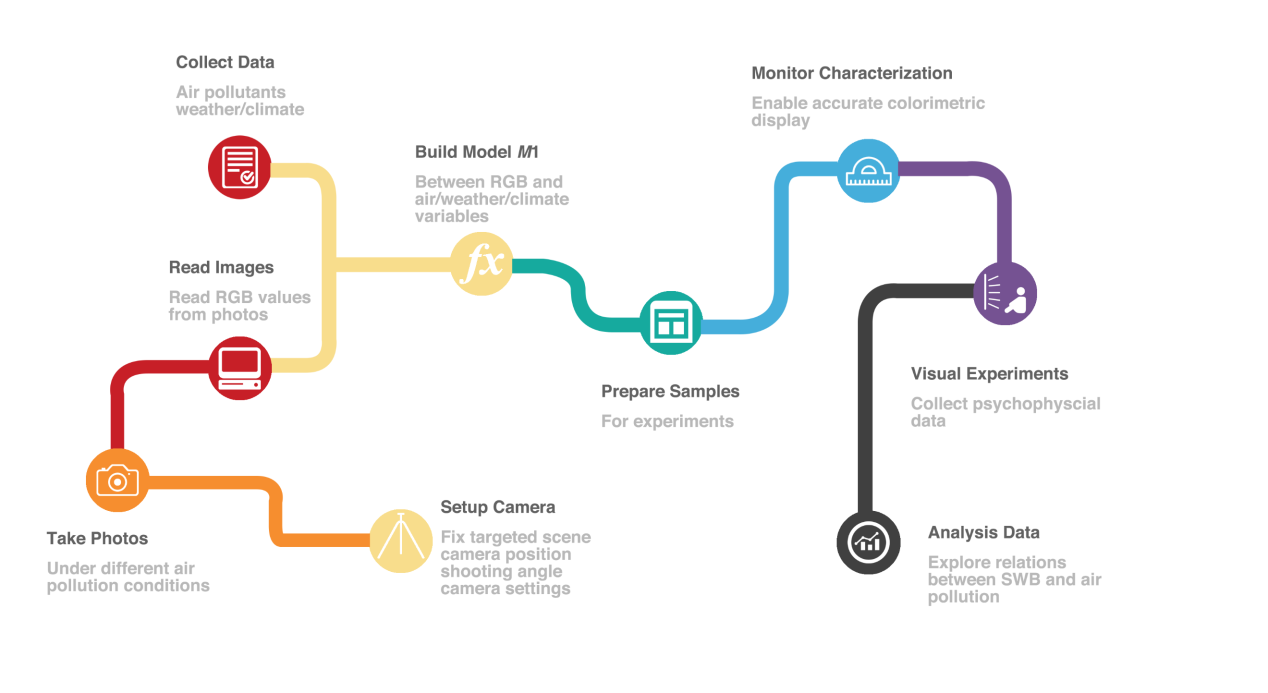


## Supplementary Figure 1: The workflow of this research. The research started with collection of air pollutant data (red) and colour data of scene for each air pollution level (red). Model 1 was built based on colour data and air pollution data (yellow) and the experimental samples were produced based on Model 1 (green). After monitor characterization (blue), visual experiment was conducted (purple).

## Supplementary Figure 2: Relations between PM_2.5_ and other air pollutants. The subfigures shows the data and fitted curve between PM_2.5_ and O_3_, NO_2_, SO_2_, and CO. The r values in each subfigures shows the model performance of each fitting.

## Supplementary Figure 3: Simulated air quality images with various PM_2.5_ values. These 18 images with same scene but different air pollution levels were used in visual experiment as samples for observers.


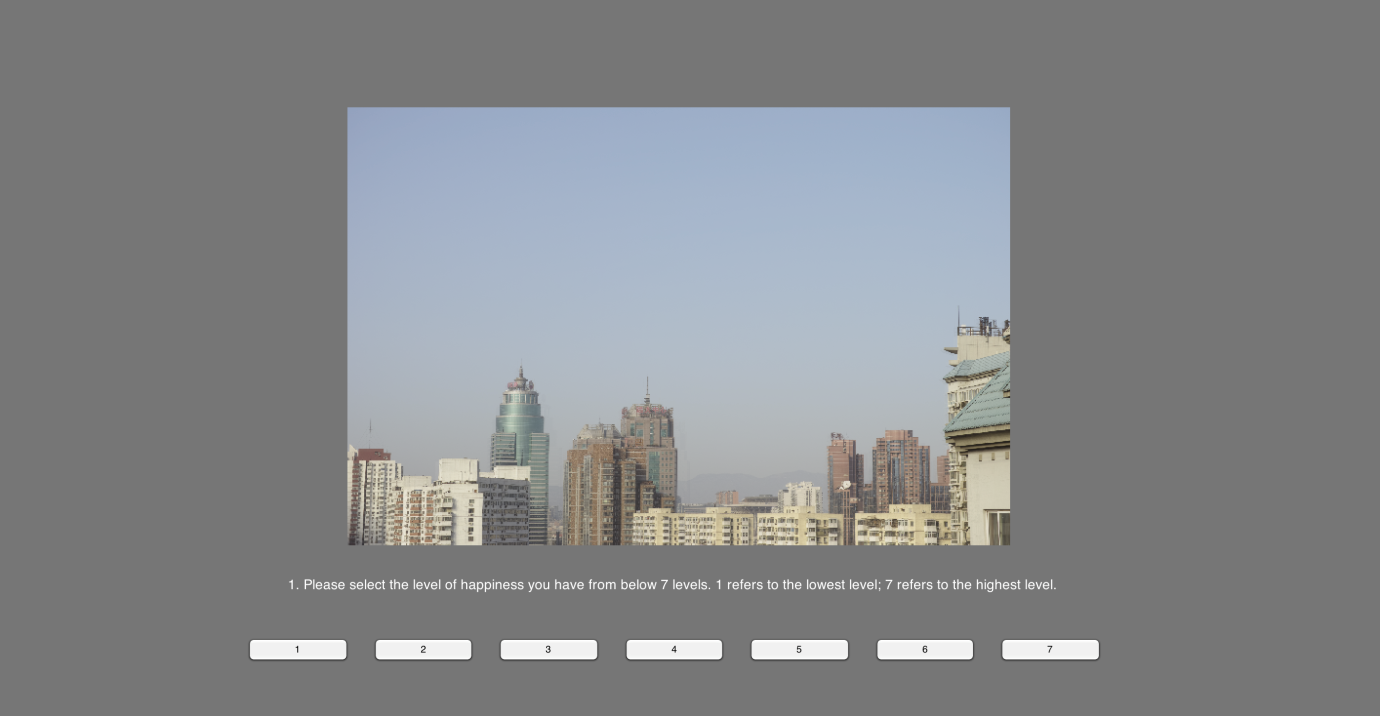


## Supplementary Figure 4: The typical GUI for the categorical judgment experiment. Experimental interface shows how experiment going on. Observers have to judge their emotion levels for example happiness according to the level of air pollution shown in the displayed image. After clicking one button for their choice, a new image with different air pollution level will display.

## **Supplementary Figure 5: Raw z-score data for Figure 1.** PM_2.5_ is selected to represent the increase in air pollution combinations, including PM_2.5_, SO_2_, NO_2_, O_3_ and CO. As PM_2.5_ increases, the other pollutants increase with fixed ratios. a, b, The changes in the positive emotions of happiness (dark red) and expectation (light green) with changes in air pollution. c, d, e, f, The changes in the negative emotions of worry (green), stress (purple), depression (sky blue) and irritation (yellow) with changes in air pollution. g, The changes in SWB with changes in air pollution. SWB is in the perceptual range of [-5 5]. A SWB of 5 represents the most positive condition (magenta); a SWB of 0 represents a neutral condition (beige); a SWB of -5 represents the most negative condition (dark brown).

## **Supplementary Figure 6: Raw z-scores data for Figure 2.** a, the effect of whether or not people have children on their SWB under air pollution (red refers to yes, and blue refers to no); b, gender has little effect on people’s SWB (red refers to female and blue refers to male); c, the effect of age group on SWB (red refers to people younger than 21; blue refers to people between 21 and 30; green refers to people between 31 and 40; grey refers to people older than 40); d, the effects of attitude towards the necessity of wearing a mask on SWB (red refers to people who always wear masks; blue refers to people who wear masks when there is air pollution; green refers to people who wear masks only during severe air pollution; grey refers to people who never wear masks when going outside); e, the effect of knowledge of the harmfulness of air pollution on SWB (red refers to people who think the harmfulness of air pollution is more serious than smoking; blue refers to people who think the harmfulness of air pollution is similar to smoking; green refers to people who think the harmfulness of air pollution is less serious than smoking; grey refers to people who think air pollution is harmless and causes only discomfort); f, the effect of the average daily exposure time to outdoor air on SWB (red refers to people who stay outdoors less than 3 hours each day; blue refers to people who stay outdoors between 3 and 4 hours each day; green refers to people who stay outdoors more than 4 hours each day).

## Supplementary Figure 7: Raw Z-Score data for Figure 3. a, b, The change in the positive emotions of happiness and future expectations with changes in air pollution. c, d, e, f, The changes in the negative emotions of worry, stress, depression and irritation with changes in air pollution. g, The changes in SWB with changes in air pollution. Positive and negative emotions and SWB are compared between observers living in China and the UK. The red line indicates that the observers in China have stronger emotions than those in the UK under the given air pollution condition (PM_2.5_); the blue line indicates that the observers in the UK have stronger emotions than those in China under the given air pollution condition (PM_2.5_).

## Supplementary Figure 8: Polynomial fitting of SWB against air pollution between China and UK age groups. a, the effect of country background on their SWB under air pollution for people under 31 (red refers to Chinese, and blue refers to UK); b, the effect of country background on their SWB under air pollution for people above 30 (red refers to Chinese, and blue refers to UK);c, the effect of age group on SWB for Chinese (pink refers to people younger than 31; dark red refers to people above 30); d, the effect of age group on SWB for the British (light blue refers to people younger than 31; dark blue refers to people above 30).

## Supplementary Figure 9: Polynomial fitting of SWB against air pollution between China and UK gender groups. a, the effect of country background on their SWB under air pollution for male (red refers to Chinese, and blue refers to UK); b, the effect of country background on their SWB under air pollution for female (red refers to Chinese, and blue refers to UK); c, the effect of gender on SWB for Chinese (pink refers to female; dark red refers to male); d, the effect of gender on SWB for British (light blue refers to female; dark blue refers to male).

## Supplementary Figure 10: Polynomial fitting of SWB against air pollution between China and UK exposure time groups. a, the effect of country background on their SWB under air pollution for people spending 2 hours or less outdoors daily (red refers to Chinese, and blue refers to UK); b, the effect of country background on their SWB under air pollution for people spending more than 2 hours outdoors daily (red refers to Chinese, and blue refers to UK); c, the effect of daily outdoor exposure time on SWB for Chinese (pink refers to female; dark red refers to male); d, the effect of daily outdoor exposure time on SWB for the British (light blue refers to female; dark blue refers to male).

## Supplementary Figure 11: Polynomial fitting of SWB against air pollution between China and UK knowledge impact groups. a, the effect of country background on their SWB under air pollution for people believing air pollution is similar to or even more harmful than smoking (red refers to Chinese, and blue refers to UK); b, the effect of country background on their SWB under air pollution for people believing air pollution is less harmful than smoking or no effect on health (red refers to Chinese, and blue refers to UK); c, the effect of the knowledge of the harmfulness of air pollution on SWB for Chinese (pink refers to female; dark red refers to male); d, the effect of the knowledge of the harmfulness of air pollution on SWB for the British (light blue refers to female; dark blue refers to male).

# Supplementary Tables

| PM_2.5_ | 5 | 20 | 40 | 60 | 80 | 100 | 120 | 140 | 160 | 180 | 200 | 220 | 240 | 260 | 280 | 300 | 320 | 340 |
| --- | --- | --- | --- | --- | --- | --- | --- | --- | --- | --- | --- | --- | --- | --- | --- | --- | --- | --- |
| Happiness | 4.22 | 3.82 | 3.93 | 3.76 | 3.28 | 2.93 | 3.06 | 2.72 | 2.42 | 2.04 | 2.15 | 1.85 | 1.77 | 1.63 | 1.59 | 1.50 | 1.20 | 1.12 |
| Expectation | 4.34 | 4.09 | 3.94 | 3.49 | 3.41 | 3.15 | 2.84 | 2.62 | 2.19 | 1.80 | 1.91 | 1.79 | 1.74 | 1.63 | 1.64 | 1.57 | 1.40 | 1.46 |
| Worry | 0.82 | 0.94 | 1.16 | 1.33 | 1.62 | 1.99 | 2.14 | 2.24 | 2.71 | 2.89 | 2.92 | 3.06 | 3.23 | 3.54 | 3.35 | 3.44 | 3.80 | 3.82 |
| Stress | 0.65 | 1.02 | 1.19 | 1.36 | 1.77 | 1.88 | 2.03 | 2.59 | 2.47 | 2.83 | 2.93 | 3.07 | 3.32 | 3.45 | 3.48 | 3.59 | 3.68 | 3.70 |
| Depression | 0.52 | 0.70 | 1.41 | 1.52 | 1.75 | 1.89 | 2.31 | 2.51 | 2.61 | 2.81 | 2.97 | 3.31 | 3.13 | 3.16 | 3.48 | 3.44 | 3.70 | 3.77 |
| Irritation | 0.36 | 0.78 | 1.42 | 1.59 | 1.72 | 1.89 | 2.28 | 2.65 | 2.55 | 2.93 | 2.99 | 3.10 | 3.21 | 3.37 | 3.46 | 3.48 | 3.56 | 3.68 |
| SWB | 3.70 | 3.10 | 2.64 | 2.17 | 1.63 | 1.13 | 0.76 | 0.17 | -0.28 | -0.95 | -0.92 | -1.32 | -1.47 | -1.75 | -1.82 | -1.95 | -2.39 | -2.45 |

## Supplementary Table 1: The z-scores for 18 simulated images on all feelings and SWB for all observers. These are the processed perception data after standardization. Each column refers to a certain air pollution level and each row refers to a perception including Happiness, Expectation, Worry, Stress, Depression, Irritation and Subjective Well Being.

|  | a  Happiness | b  Future Expectations | c  Worry | d  Stress | e  Depression | f  Irritation | g  SWB |
| --- | --- | --- | --- | --- | --- | --- | --- |
| order of polynomial | 4 | 4 | 4 | 2 | 3 | 3 | 5 |
| coefficient 1 | -7.00E-10 | -8.42E-10 | 7.09E-10 | -1.77E-05 | 6.84E-08 | 6.10E-08 | -6.43E-12 |
| coefficient 2 | 5.05E-07 | 6.27E-07 | -5.09E-07 | 0.0153 | -5.73E-05 | -5.70E-05 | 4.68E-09 |
| coefficient 3 | -0.0001 | -0.0001 | 0.0001 | 0.6004 | 0.0214 | 0.0220 | -1.09E-06 |
| coefficient 4 | -0.0041 | -0.0055 | 0.0051 |  | 0.4146 | 0.3742 | 0.0001 |
| coefficient 5 | 4.1479 | 4.3060 | 0.7984 |  |  |  | -0.0315 |
| coefficient 6 |  |  |  |  |  |  | 3.7848 |
| degrees of freedom | 13 | 13.0000 | 13 | 15 | 14 | 14 | 12 |
| norm of the residuals | 0.4801 | 0.3945 | 0.3645 | 0.3612 | 0.4401 | 0.4228 | 0.4000 |
| R-Squared | 0.9864 | 0.9908 | 0.9922 | 0.9923 | 0.9886 | 0.9895 | 0.9976 |

## Supplementary Table 2: Polynomial Regression statistics For Figure 1. This table shows the statistics information for the fittings in all subfigures in Figure 1. The statistics includes order of polynomial, coefficients of models and degree of freedom, norm of the residuals and R squared. The columns refer to each subfigure from a to g.


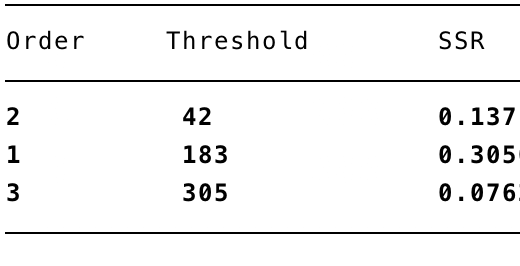


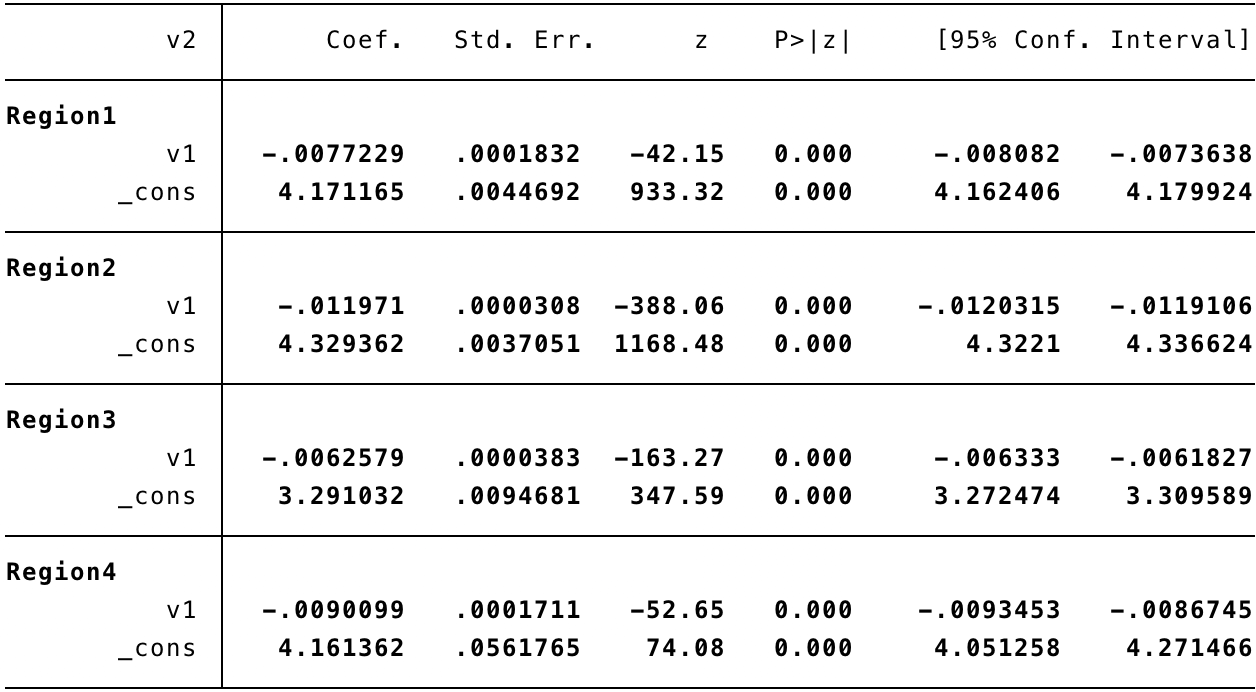


## Supplementary Table 3: Threshold Regression statistics For Figure1a (Happiness). The upper table shows the suggested thresholds including the order of significance (first and third column) and their values/locations in PM_2.5_ scale (second column). The lower table shows the curve trend for each region according to the thresholds listed in the upper table (from the second row to the bottom row). The Coef. (in column) and v1 (in row) refers to the slope of the curve in a region and the Coef. (in column) and cons (in row) refers to the intercept in that region.


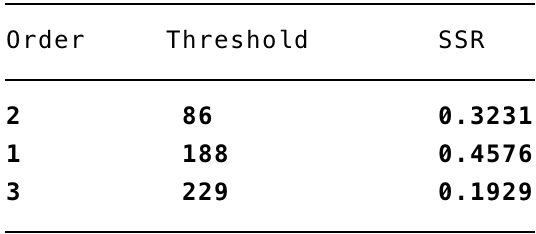


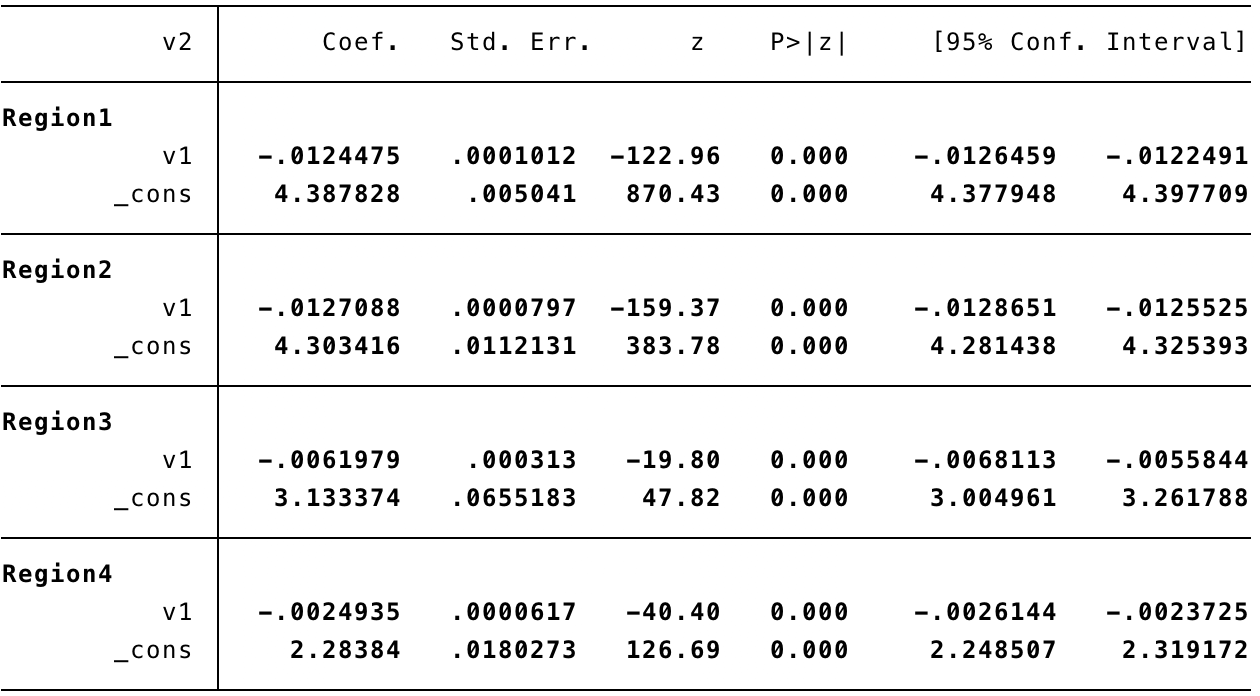


## Supplementary Table 4: Threshold Regression statistics For Figure1b (Future Expectations). The upper table shows the suggested thresholds including the order of significance (first and third column) and their values/locations in PM_2.5_ scale (second column). The lower table shows the curve trend for each region according to the thresholds listed in the upper table (from the second row to the bottom row). The Coef. (in column) and v1 (in row) refers to the slope of the curve in a region and the Coef. (in column) and cons (in row) refers to the intercept in that region.


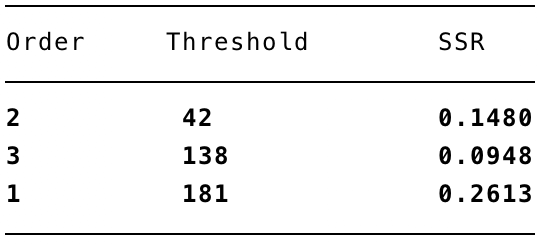

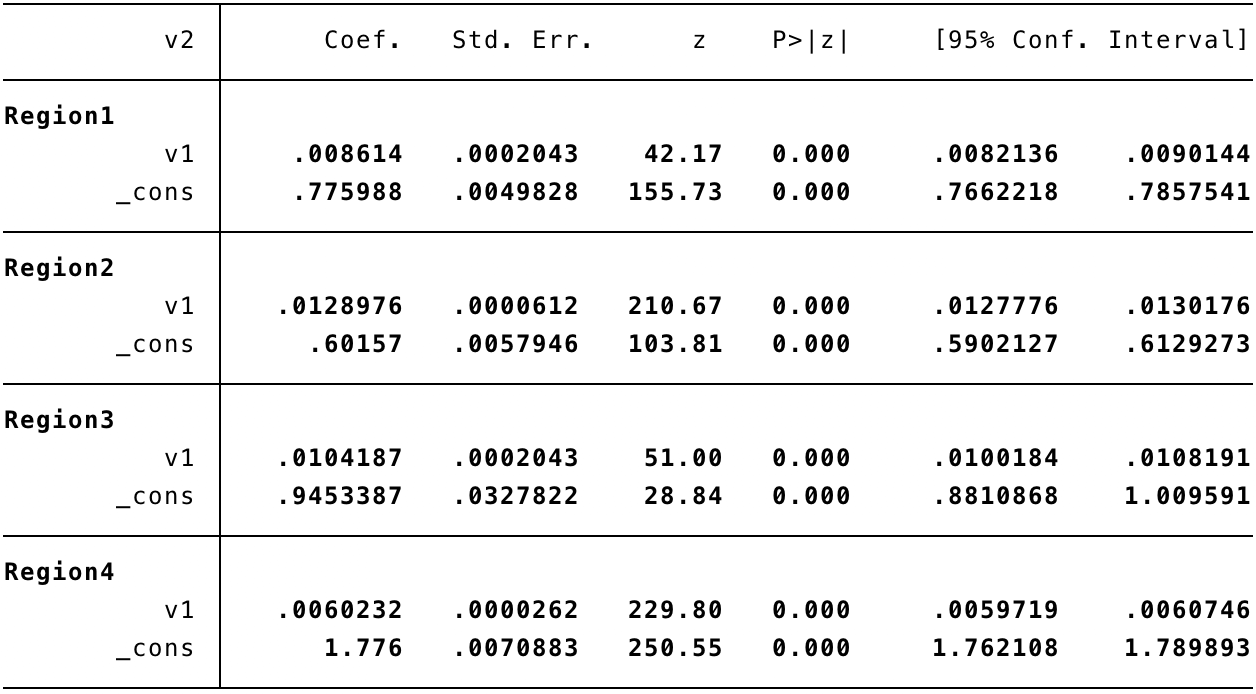


## Supplementary Table 5: Threshold Regression statistics For Figure1c (Worry). The upper table shows the suggested thresholds including the order of significance (first and third column) and their values/locations in PM_2.5_ scale (second column). The lower table shows the curve trend for each region according to the thresholds listed in the upper table (from the second row to the bottom row). The Coef. (in column) and v1 (in row) refers to the slope of the curve in a region and the Coef. (in column) and cons (in row) refers to the intercept in that region.


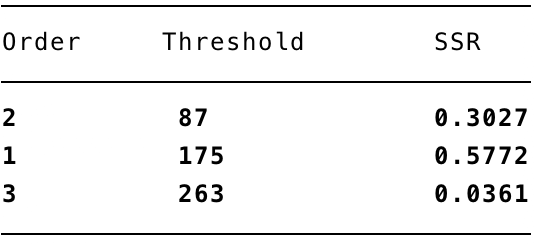

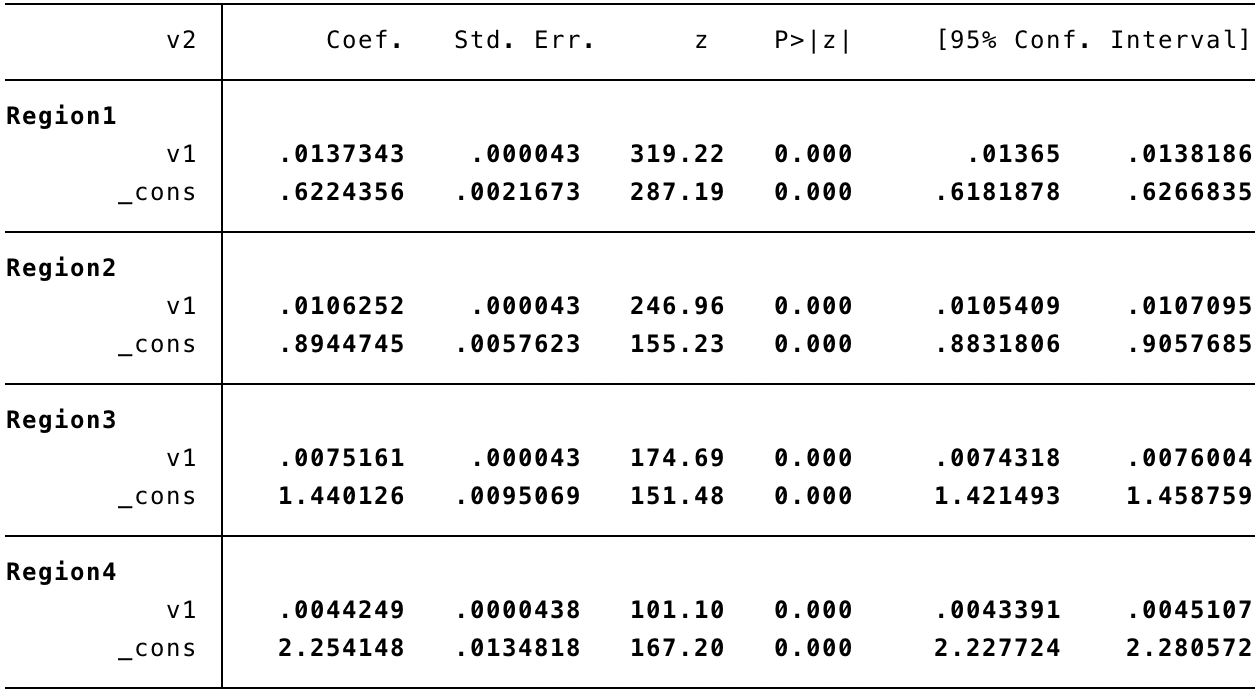


## Supplementary Table 6: Threshold Regression statistics For Figure1d (Stress). The upper table shows the suggested thresholds including the order of significance (first and third column) and their values/locations in PM_2.5_ scale (second column). The lower table shows the curve trend for each region according to the thresholds listed in the upper table (from the second row to the bottom row). The Coef. (in column) and v1 (in row) refers to the slope of the curve in a region and the Coef. (in column) and cons (in row) refers to the intercept in that region.


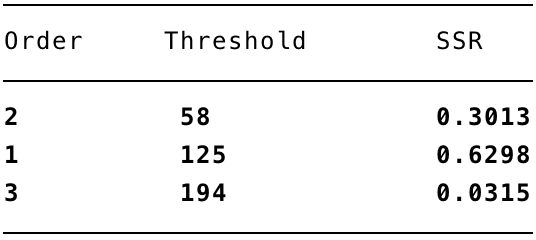

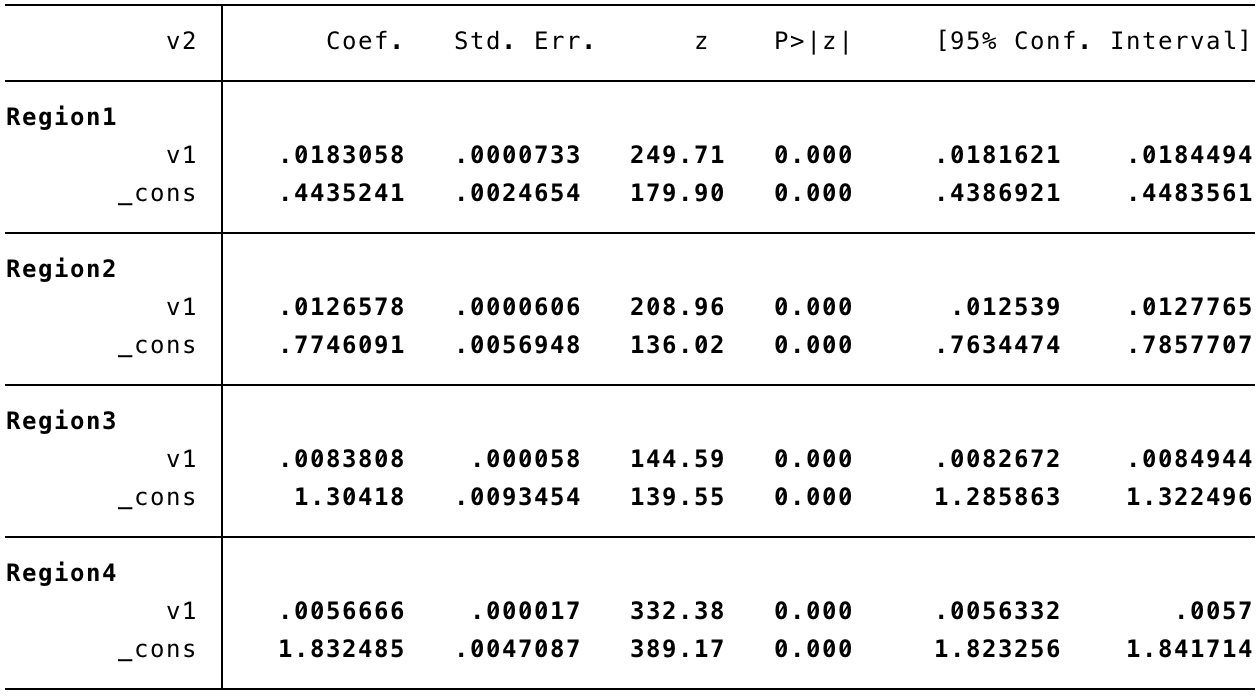


## Supplementary Table 7: Threshold Regression statistics For Figure1e (Depression). The upper table shows the suggested thresholds including the order of significance (first and third column) and their values/locations in PM_2.5_ scale (second column). The lower table shows the curve trend for each region according to the thresholds listed in the upper table (from the second row to the bottom row). The Coef. (in column) and v1 (in row) refers to the slope of the curve in a region and the Coef. (in column) and cons (in row) refers to the intercept in that region.


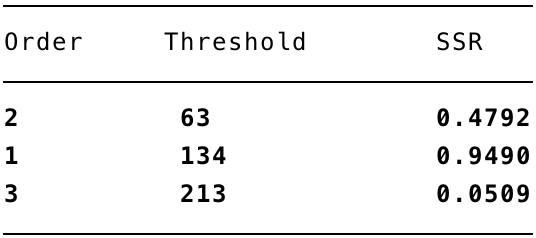

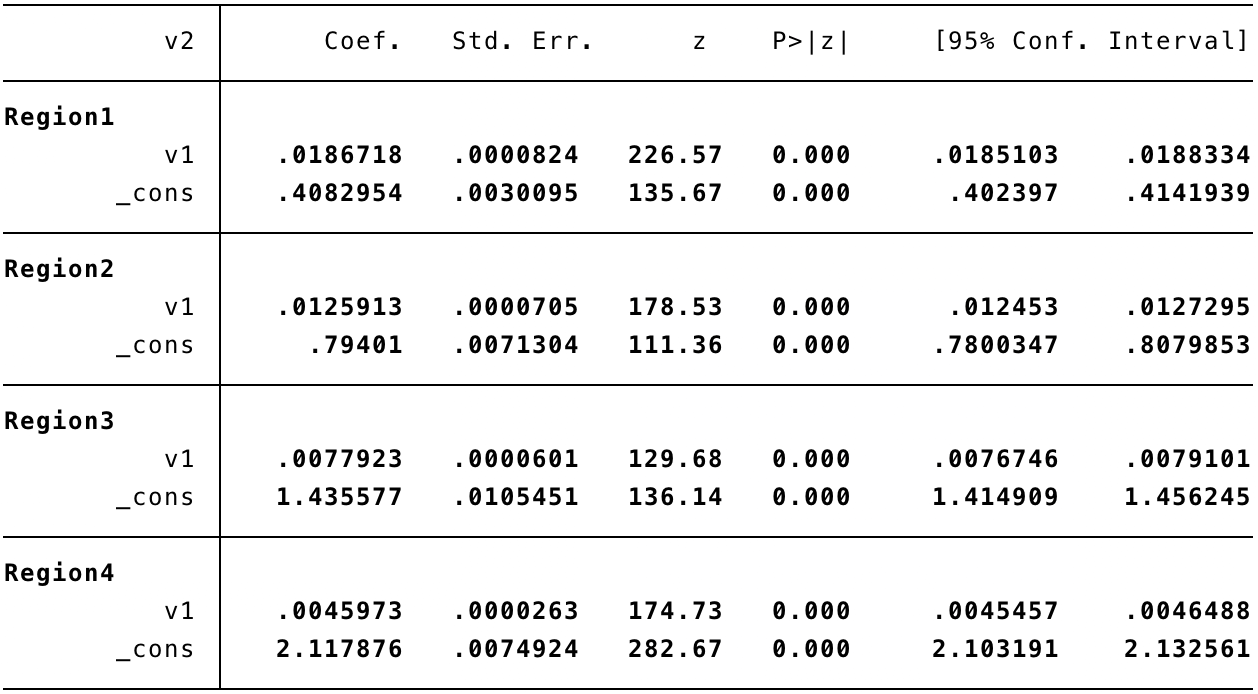


## Supplementary Table 8: Threshold Regression statistics For Figure1f (Irritation). The upper table shows the suggested thresholds including the order of significance (first and third column) and their values/locations in PM_2.5_ scale (second column). The lower table shows the curve trend for each region according to the thresholds listed in the upper table (from the second row to the bottom row). The Coef. (in column) and v1 (in row) refers to the slope of the curve in a region and the Coef. (in column) and cons (in row) refers to the intercept in that region.


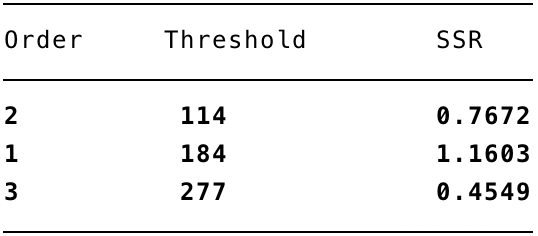

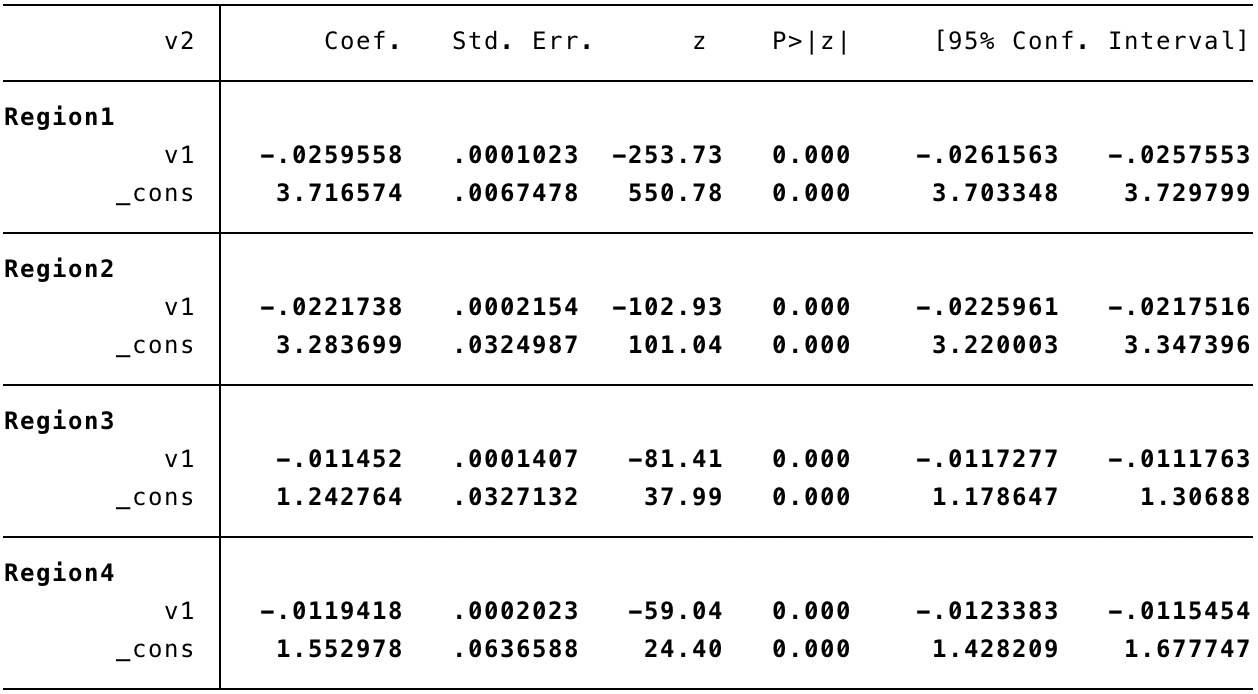


## Supplementary Table 9: Threshold Regression statistics For Figure1g (SWB). The upper table shows the suggested thresholds including the order of significance (first and third column) and their values/locations in PM_2.5_ scale (second column). The lower table shows the curve trend for each region according to the thresholds listed in the upper table (from the second row to the bottom row). The Coef. (in column) and v1 (in row) refers to the slope of the curve in a region and the Coef. (in column) and cons (in row) refers to the intercept in that region.

|  | Childen_yes | Children_no |
| --- | --- | --- |
| order of polynomial | 3 | 3 |
| coefficient 1 | -3.32E-08 | 1.28E-07 |
| coefficient 2 | 6.64E-05 | -3.33E-05 |
| coefficient 3 | -0.03706769 | -2.04E-02 |
| coefficient 4 | 4.024908984 | 3.47E+00 |
| degrees of freedom | 14 | 14 |
| norm of the residuals | 0.8886 | 0.7226 |
| R-Squared | 0.9881 | 0.9921 |

## Supplementary Table 10: Polynomial Regression statistics For Figure2a. This table shows the statistics information for the curve fittings in Figure 2a. The statistics includes order of polynomial, coefficients of models and degree of freedom, norm of the residuals and R squared. The columns refer to the status of observers regarding to having children.


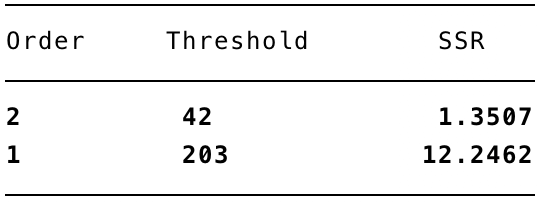

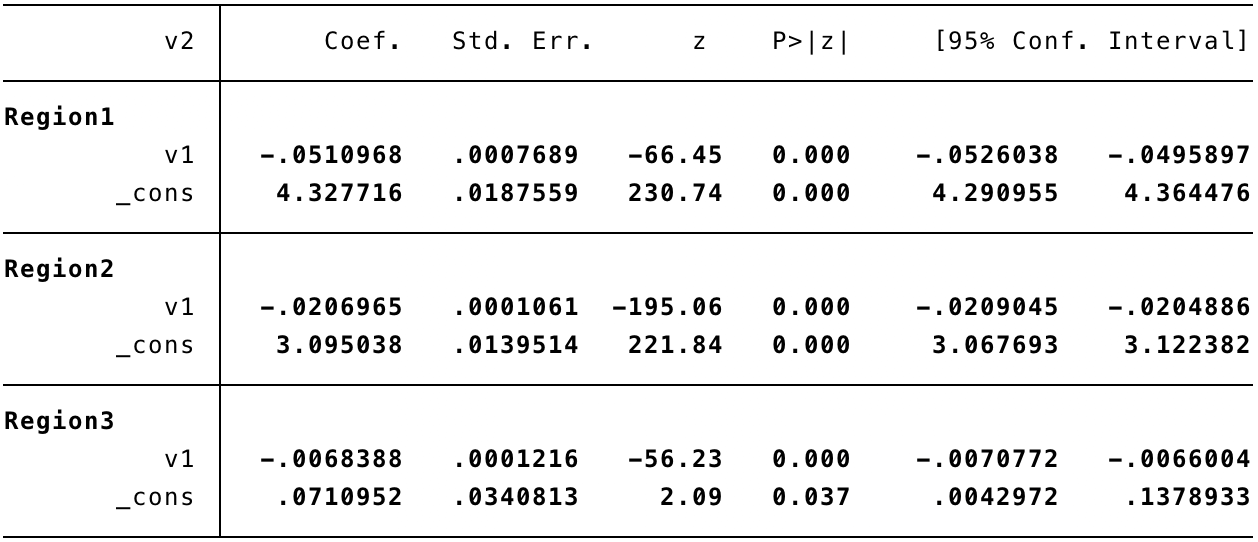


## Supplementary Table 11: Threshold Regression statistics For Figure2a (children-yes). The upper table shows the suggested thresholds including the order of significance (first and third column) and their values/locations in PM_2.5_ scale (second column). The lower table shows the curve trend for each region according to the thresholds listed in the upper table (from the second row to the bottom row). The Coef. (in column) and v1 (in row) refers to the slope of the curve in a region and the Coef. (in column) and cons (in row) refers to the intercept in that region.


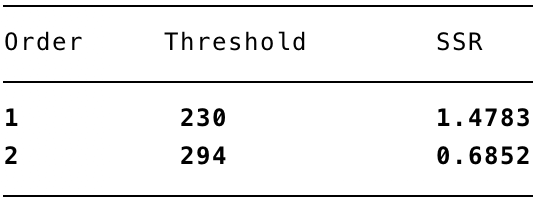

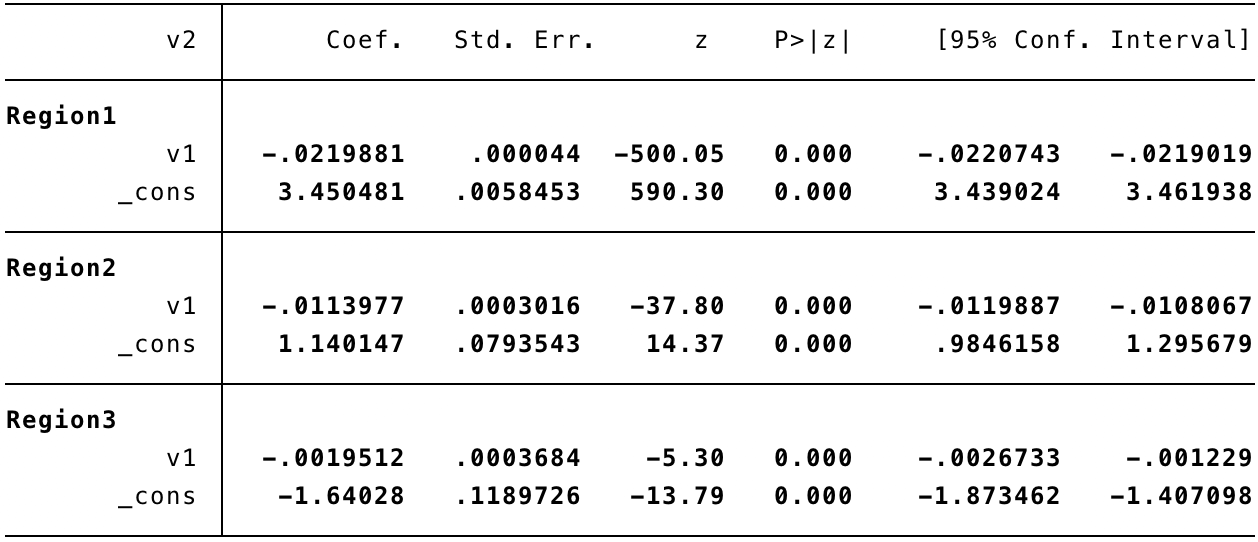


## Supplementary Table 12: Threshold Regression statistics For Figure2a (children-no). The upper table shows the suggested thresholds including the order of significance (first and third column) and their values/locations in PM_2.5_ scale (second column). The lower table shows the curve trend for each region according to the thresholds listed in the upper table (from the second row to the bottom row). The Coef. (in column) and v1 (in row) refers to the slope of the curve in a region and the Coef. (in column) and cons (in row) refers to the intercept in that region.

|  | female | male |
| --- | --- | --- |
| order of polynomial | 3 | 3 |
| coefficient 1 | 2.82E-08 | 2.05E-08 |
| coefficient 2 | 2.69E-05 | 2.72E-05 |
| coefficient 3 | -0.0304 | -0.0298 |
| coefficient 4 | 3.8212 | 3.7741 |
| degrees of freedom | 14 | 14 |
| norm of the residuals | 0.5732 | 0.4249 |
| R-Squared | 0.9951 | 0.9973 |

## Supplementary Table 13: Polynomial Regression statistics For Figure2b. This table shows the statistics information for the curve fittings in Figure 2b. The statistics includes order of polynomial, coefficients of models and degree of freedom, norm of the residuals and R squared. The columns refer to the gender of observers.


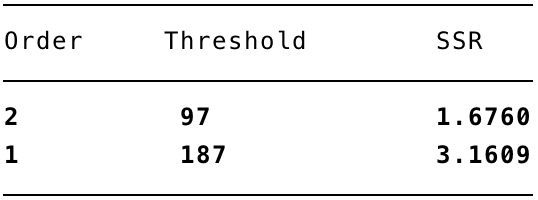

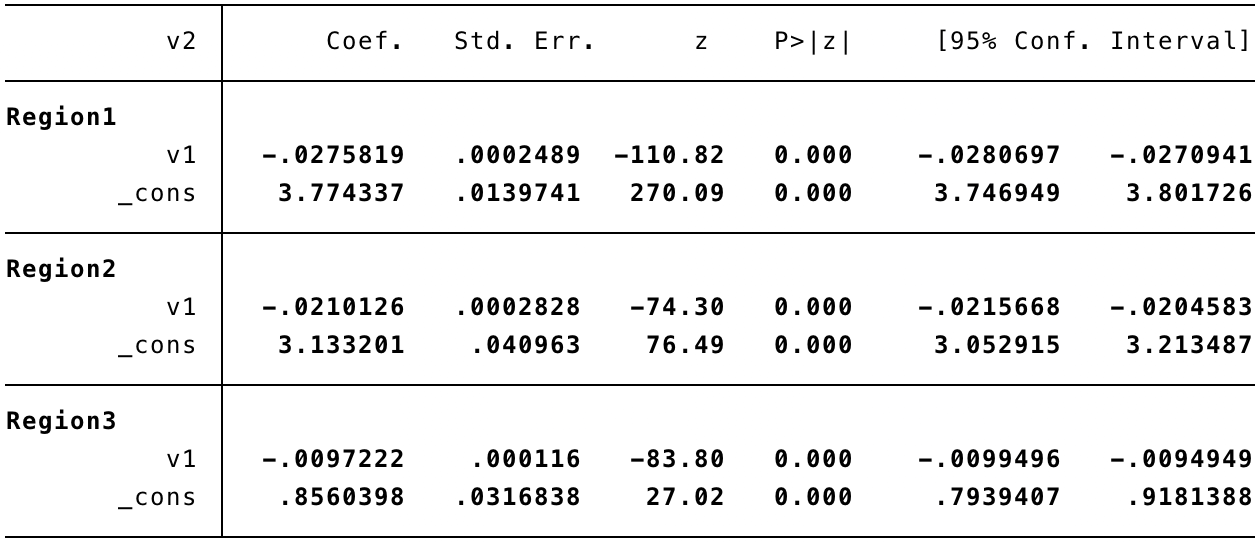


## Supplementary Table 14: Threshold Regression statistics For Figure2b (female). The upper table shows the suggested thresholds including the order of significance (first and third column) and their values/locations in PM_2.5_ scale (second column). The lower table shows the curve trend for each region according to the thresholds listed in the upper table (from the second row to the bottom row). The Coef. (in column) and v1 (in row) refers to the slope of the curve in a region and the Coef. (in column) and cons (in row) refers to the intercept in that region.


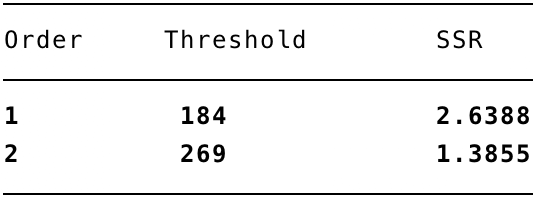

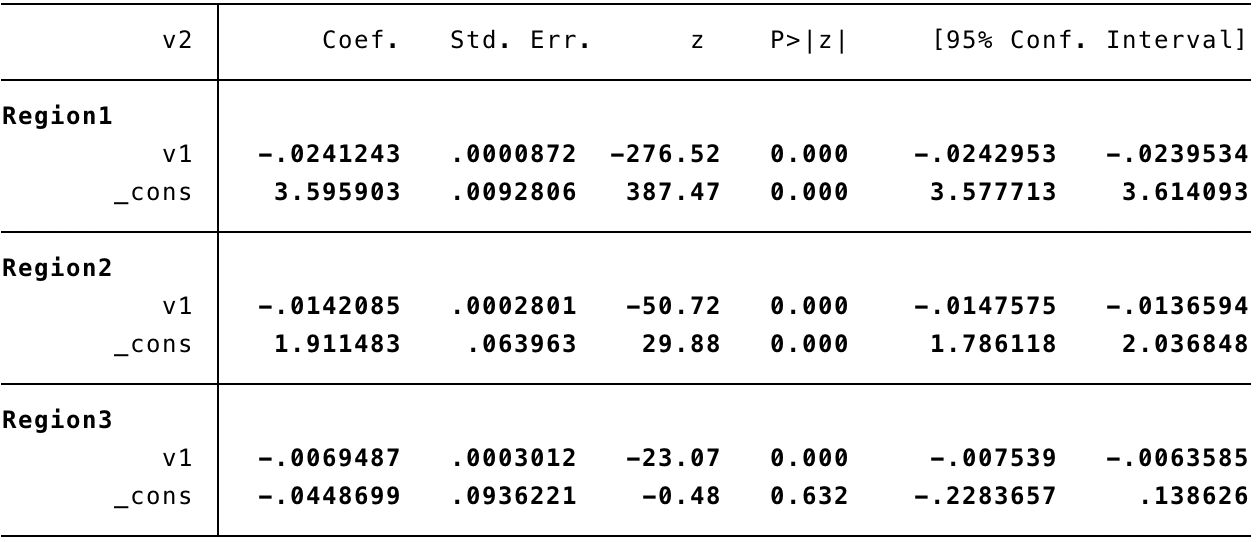


## Supplementary Table 15: Threshold Regression statistics For Figure2b (male). The upper table shows the suggested thresholds including the order of significance (first and third column) and their values/locations in PM_2.5_ scale (second column). The lower table shows the curve trend for each region according to the thresholds listed in the upper table (from the second row to the bottom row). The Coef. (in column) and v1 (in row) refers to the slope of the curve in a region and the Coef. (in column) and cons (in row) refers to the intercept in that region.

|  | younger than 21 | 21 to 30 | 31 to 40 | older than 40 |
| --- | --- | --- | --- | --- |
| order of polynomial | 3 | 3 | 3 | 3 |
| coefficient 1 | 1.07E-09 | 6.84E-08 | 1.33E-08 | -3.95E-08 |
| coefficient 2 | 2.99E-05 | 3.75E-06 | 2.23E-05 | 7.17E-05 |
| coefficient 3 | -0.0287 | -0.0268 | -0.0274 | -0.0381 |
| coefficient 4 | 3.6903 | 3.7037 | 3.64E+00 | 4.05E+00 |
| degrees of freedom | 14 | 14 | 14 | 14 |
| norm of the residuals | 0.6523 | 0.6781 | 0.7196 | 0.9849 |
| R-Squared | 0.9936 | 0.9931 | 0.9922 | 0.9853 |

## Supplementary Table 16: Polynomial Regression statistics For Figure2c. This table shows the statistics information for the curve fittings in Figure 2c. The statistics includes order of polynomial, coefficients of models and degree of freedom, norm of the residuals and R squared. The columns refer to the age of observers.


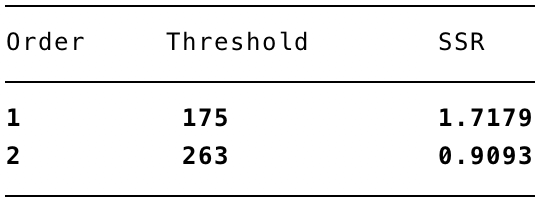

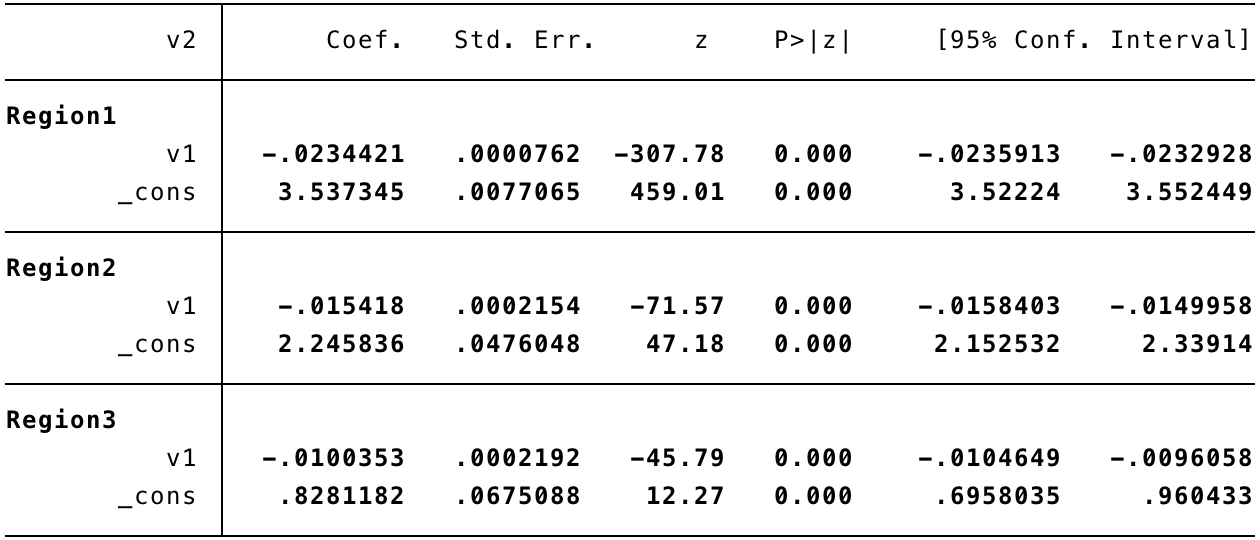


## Supplementary Table 17: Threshold Regression statistics For Figure2c (younger than 21). The upper table shows the suggested thresholds including the order of significance (first and third column) and their values/locations in PM_2.5_ scale (second column). The lower table shows the curve trend for each region according to the thresholds listed in the upper table (from the second row to the bottom row). The Coef. (in column) and v1 (in row) refers to the slope of the curve in a region and the Coef. (in column) and cons (in row) refers to the intercept in that region.


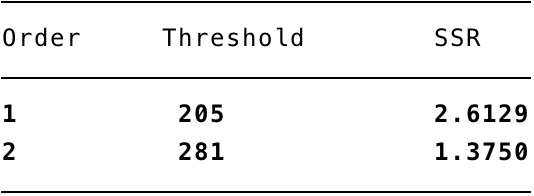

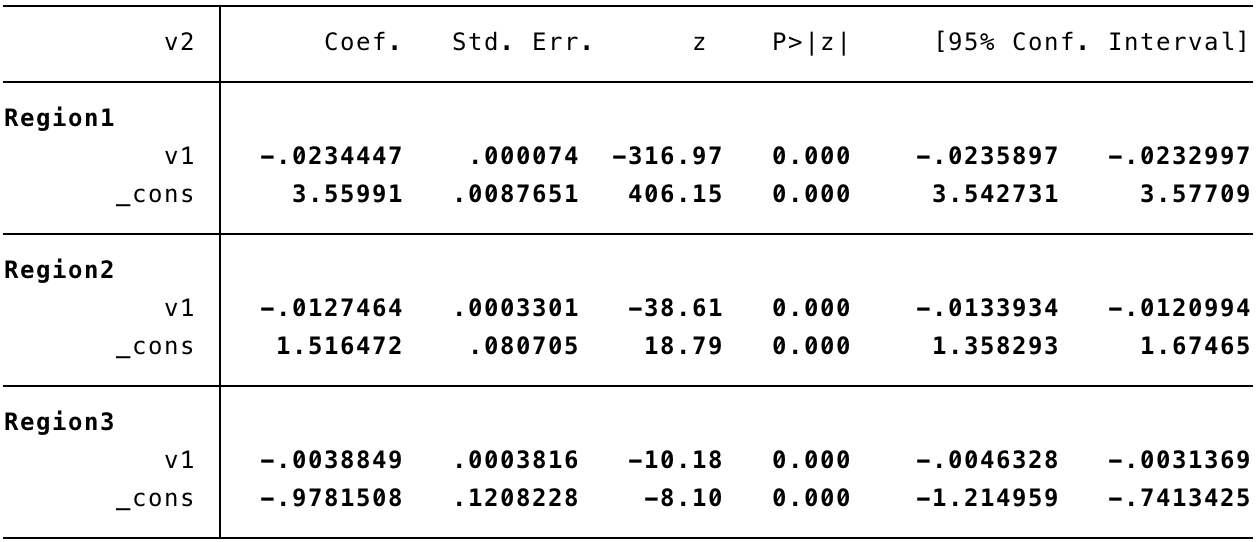


## Supplementary Table 18: Threshold Regression statistics For Figure2c (21 to 30). The upper table shows the suggested thresholds including the order of significance (first and third column) and their values/locations in PM_2.5_ scale (second column). The lower table shows the curve trend for each region according to the thresholds listed in the upper table (from the second row to the bottom row). The Coef. (in column) and v1 (in row) refers to the slope of the curve in a region and the Coef. (in column) and cons (in row) refers to the intercept in that region.


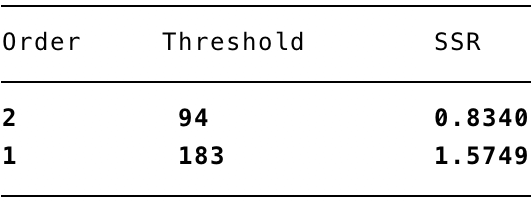

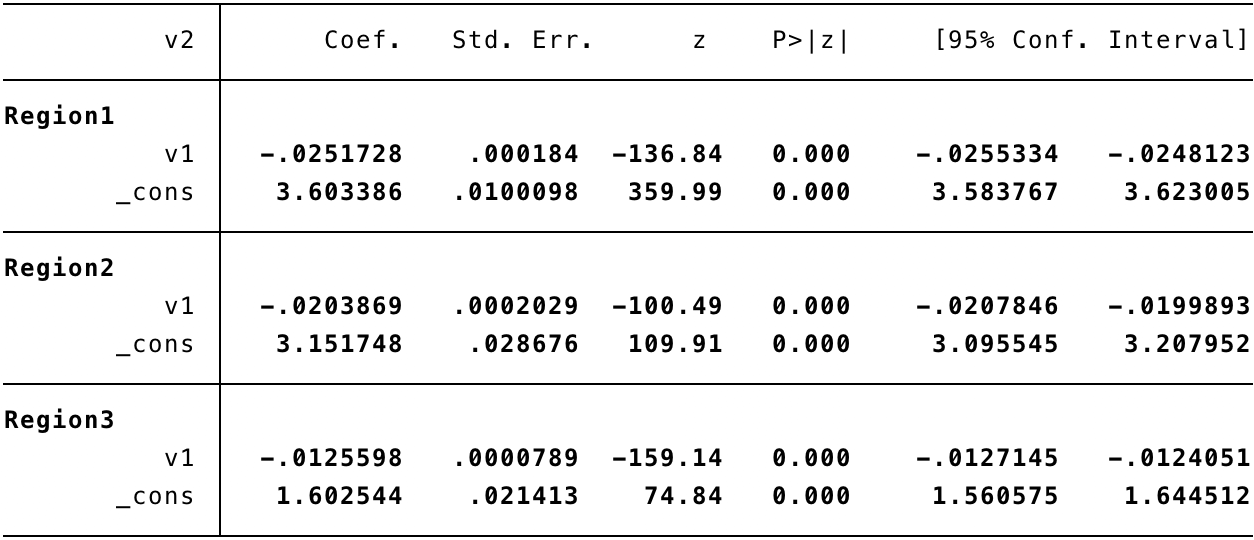


## Supplementary Table 19: Threshold Regression statistics For Figure2c (31 to 40). The upper table shows the suggested thresholds including the order of significance (first and third column) and their values/locations in PM_2.5_ scale (second column). The lower table shows the curve trend for each region according to the thresholds listed in the upper table (from the second row to the bottom row). The Coef. (in column) and v1 (in row) refers to the slope of the curve in a region and the Coef. (in column) and cons (in row) refers to the intercept in that region.


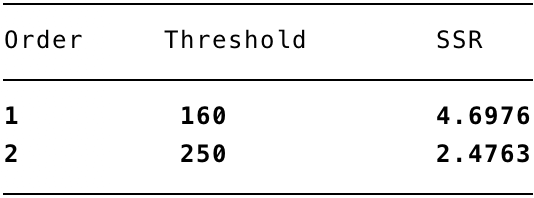

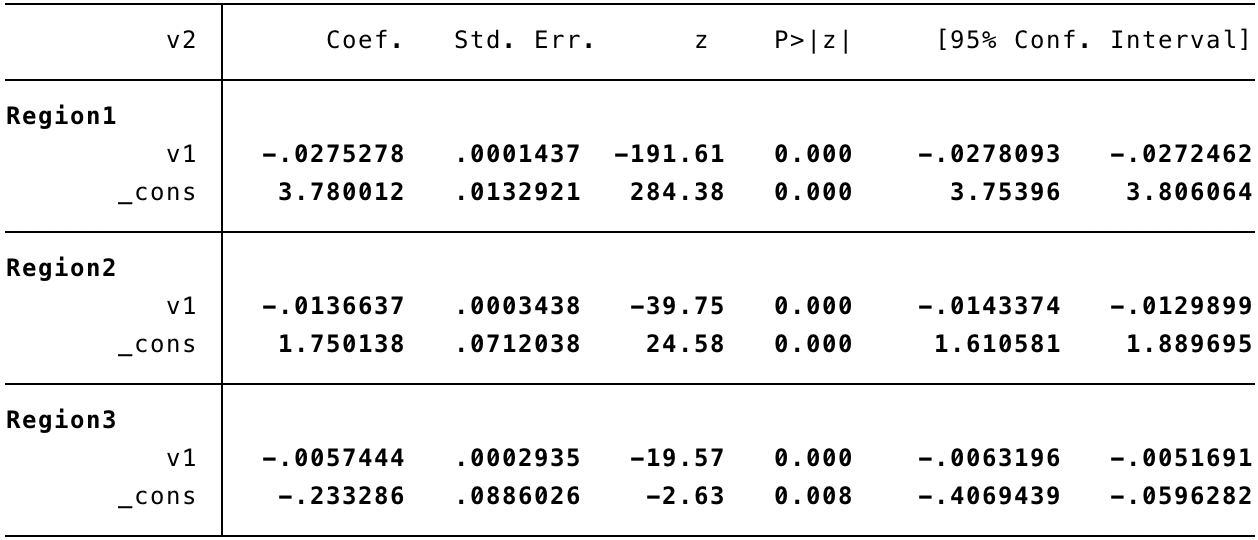


## Supplementary Table 20: Threshold Regression statistics For Figure2c (older than 40). The upper table shows the suggested thresholds including the order of significance (first and third column) and their values/locations in PM_2.5_ scale (second column). The lower table shows the curve trend for each region according to the thresholds listed in the upper table (from the second row to the bottom row). The Coef. (in column) and v1 (in row) refers to the slope of the curve in a region and the Coef. (in column) and cons (in row) refers to the intercept in that region.

|  | Always | When there is an air pollution | Only in severe air pollution | Never |
| --- | --- | --- | --- | --- |
| order of polynomial | 3 | 3 | 3 | 3 |
| coefficient 1 | 2.21E-08 | -4.35E-08 | -1.40E-08 | 7.20E-08 |
| coefficient 2 | 3.03E-05 | 5.82E-05 | 3.63E-05 | -3.38E-05 |
| coefficient 3 | -0.0309 | -3.36E-02 | -0.0293 | -0.0146282 |
| coefficient 4 | 3.8259 | 3.8587 | 3.7005 | 3.08282552 |
| degrees of freedom | 14 | 14 | 14 | 14 |
| norm of the residuals | 0.8535 | 0.5452 | 0.5873 | 0.6948 |
| R-Squared | 0.9891 | 0.9955 | 0.9948 | 0.9927 |

## Supplementary Table 21: Polynomial Regression statistics For Figure2d. This table shows the statistics information for the curve fittings in Figure 2d. The statistics includes order of polynomial, coefficients of models and degree of freedom, norm of the residuals and R squared. The columns refer to the attitude of wearing mask of observers.


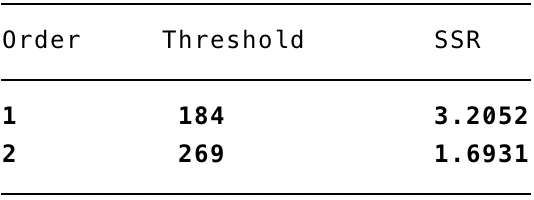

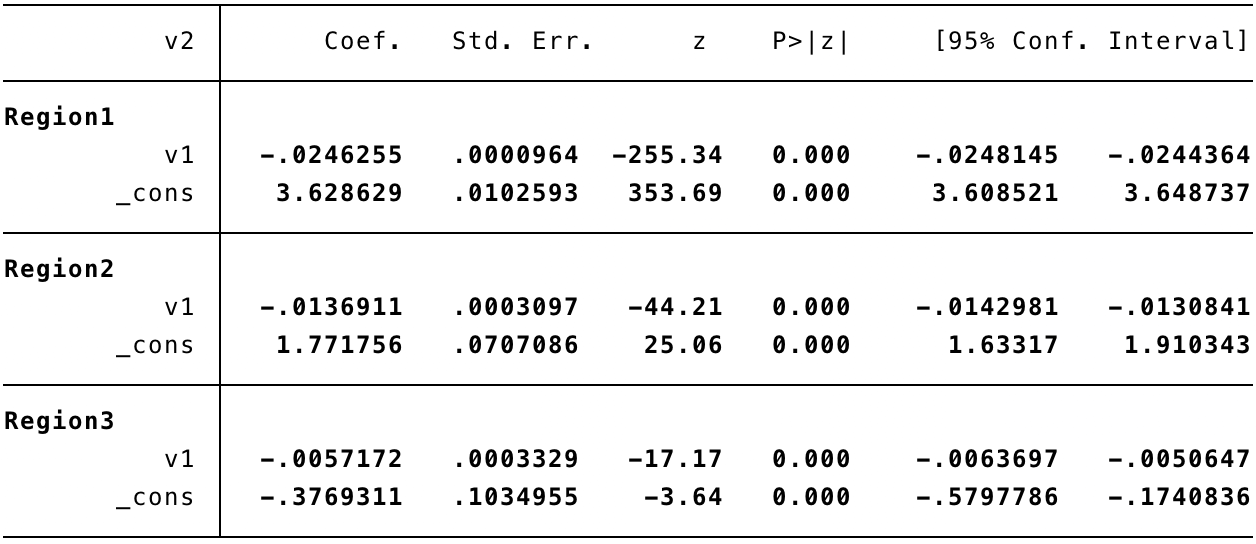


## Supplementary Table 22: Threshold Regression statistics For Figure2d (always). The upper table shows the suggested thresholds including the order of significance (first and third column) and their values/locations in PM_2.5_ scale (second column). The lower table shows the curve trend for each region according to the thresholds listed in the upper table (from the second row to the bottom row). The Coef. (in column) and v1 (in row) refers to the slope of the curve in a region and the Coef. (in column) and cons (in row) refers to the intercept in that region.


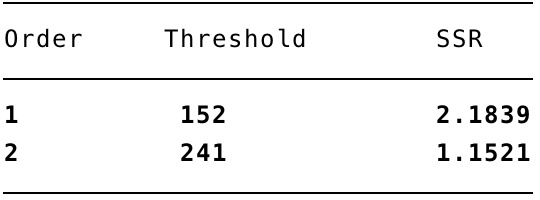

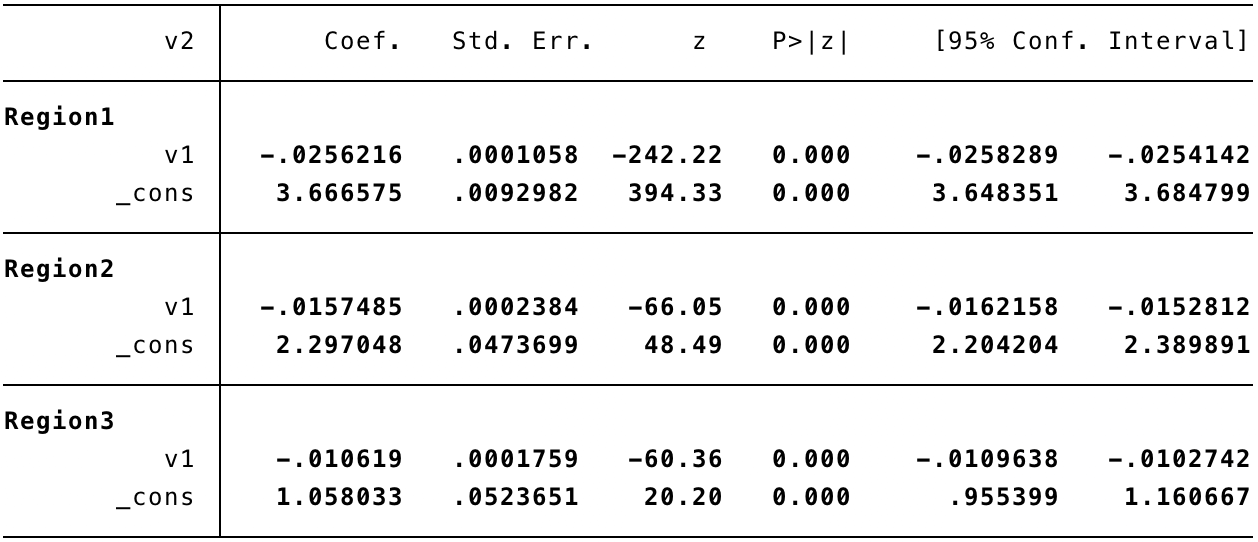


## Supplementary Table 23: Threshold Regression statistics For Figure2d (when there is an air pollution). The upper table shows the suggested thresholds including the order of significance (first and third column) and their values/locations in PM_2.5_ scale (second column). The lower table shows the curve trend for each region according to the thresholds listed in the upper table (from the second row to the bottom row). The Coef. (in column) and v1 (in row) refers to the slope of the curve in a region and the Coef. (in column) and cons (in row) refers to the intercept in that region.


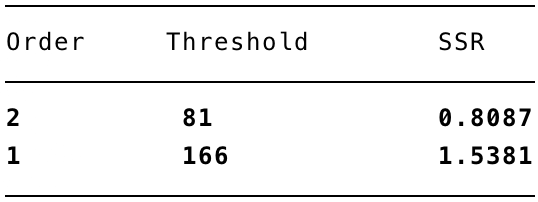

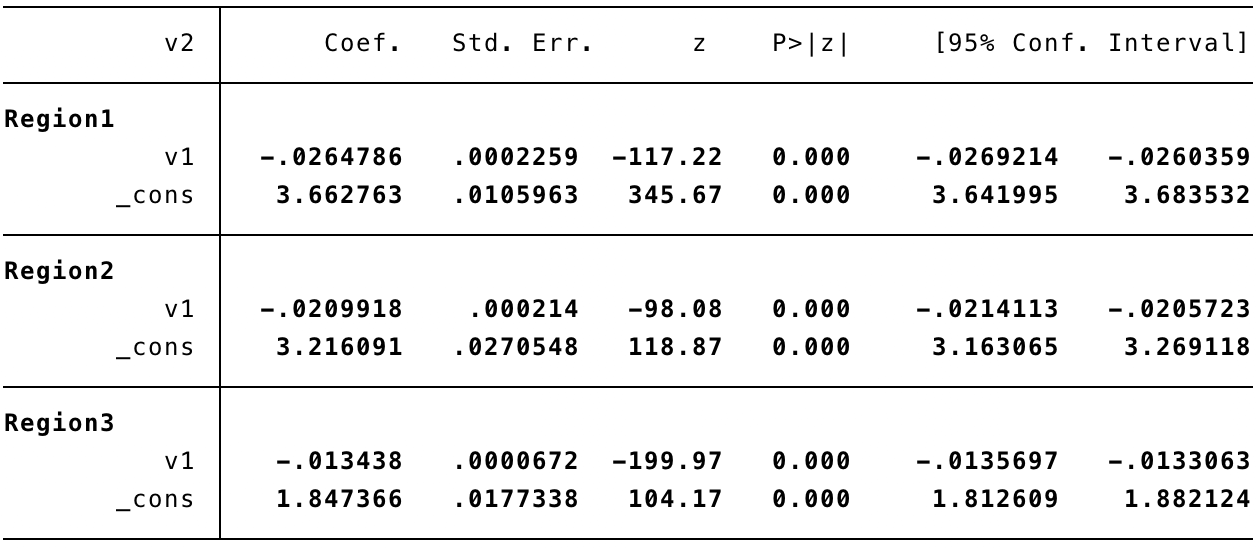


## Supplementary Table 24: Threshold Regression statistics For Figure2d (Only in severe air pollution). The upper table shows the suggested thresholds including the order of significance (first and third column) and their values/locations in PM_2.5_ scale (second column). The lower table shows the curve trend for each region according to the thresholds listed in the upper table (from the second row to the bottom row). The Coef. (in column) and v1 (in row) refers to the slope of the curve in a region and the Coef. (in column) and cons (in row) refers to the intercept in that region.


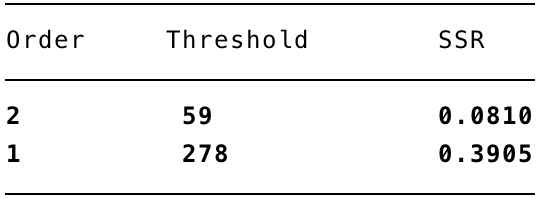

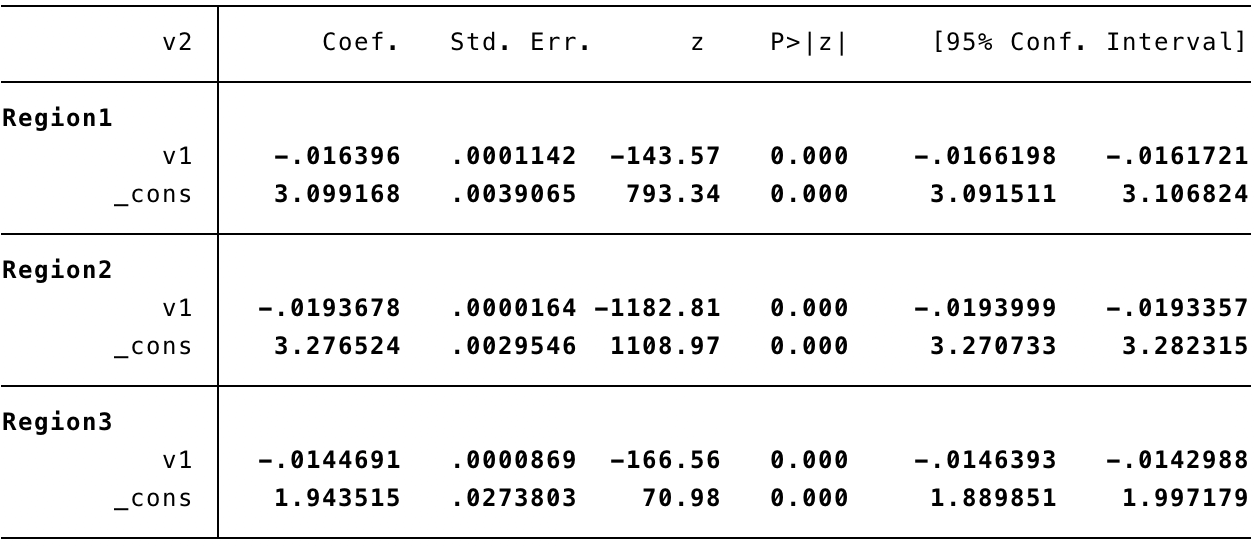


## Supplementary Table 25: Threshold Regression statistics For Figure2d (never). The upper table shows the suggested thresholds including the order of significance (first and third column) and their values/locations in PM_2.5_ scale (second column). The lower table shows the curve trend for each region according to the thresholds listed in the upper table (from the second row to the bottom row). The Coef. (in column) and v1 (in row) refers to the slope of the curve in a region and the Coef. (in column) and cons (in row) refers to the intercept in that region.

|  | More serious than smoking | Similar to smoking | Less serious than smoking |
| --- | --- | --- | --- |
| order of polynomial | 3 | 3 | 3 |
| coefficient 1 | -2.91E-08 | 8.01E-09 | 2.70E-08 |
| coefficient 2 | 4.74E-05 | 3.85E-05 | 7.35E-06 |
| coefficient 3 | -3.12E-02 | -0.0322 | -0.0239 |
| coefficient 4 | 3.73E+00 | 3.8669 | 3.5017 |
| degrees of freedom | 14 | 14 | 14 |
| norm of the residuals | 1.2582 | 0.6382 | 0.3781 |
| R-Squared | 0.9760 | 0.9939 | 0.9979 |

## Supplementary Table 26: Polynomial Regression statistics For Figure2e. This table shows the statistics information for the curve fittings in Figure 2e. The statistics includes order of polynomial, coefficients of models and degree of freedom, norm of the residuals and R squared. The columns refer to the attitude of smoking of observers.


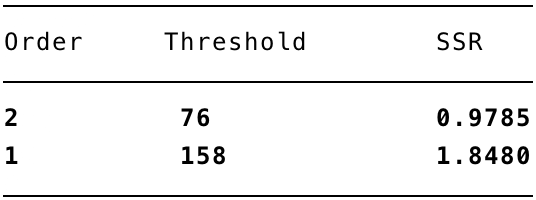

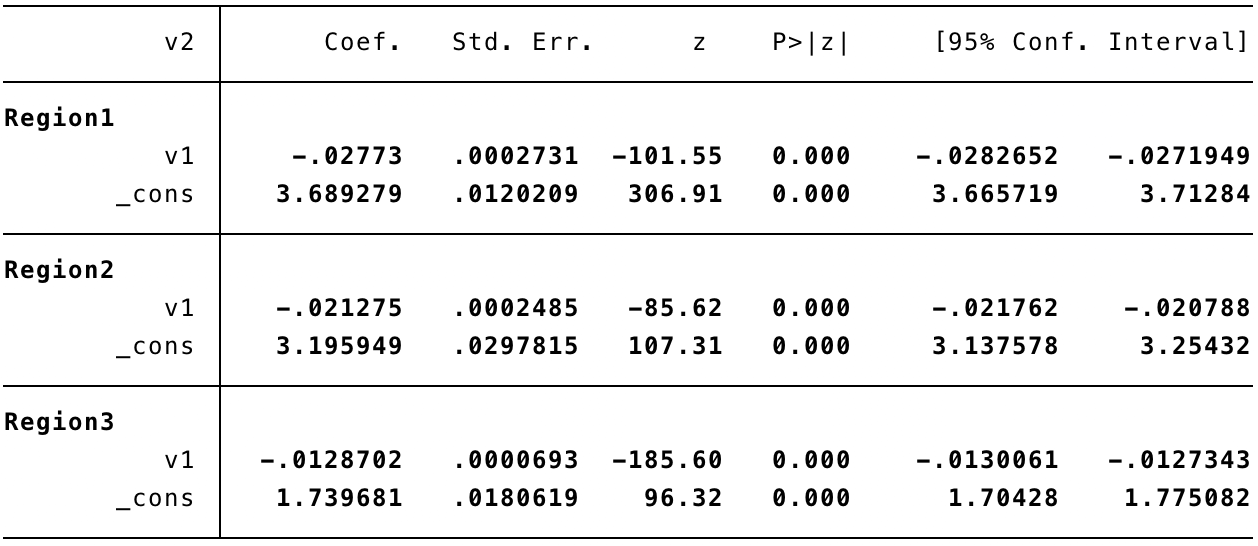


## Supplementary Table 27: Threshold Regression statistics For Figure2e (More serious than smoking). The upper table shows the suggested thresholds including the order of significance (first and third column) and their values/locations in PM_2.5_ scale (second column). The lower table shows the curve trend for each region according to the thresholds listed in the upper table (from the second row to the bottom row). The Coef. (in column) and v1 (in row) refers to the slope of the curve in a region and the Coef. (in column) and cons (in row) refers to the intercept in that region.


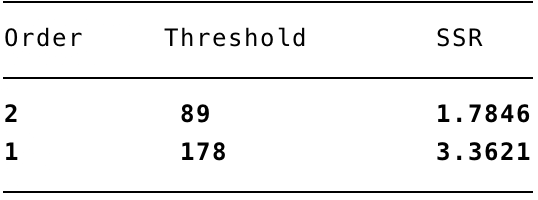

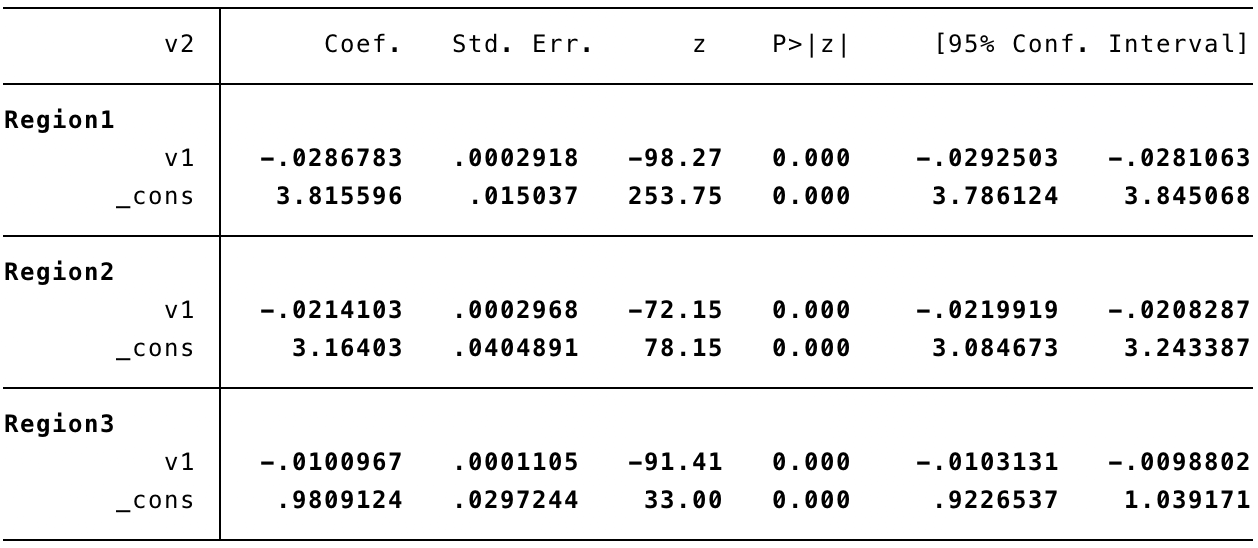


## Supplementary Table 28: Threshold Regression statistics For Figure2e (Similar to smoking). The upper table shows the suggested thresholds including the order of significance (first and third column) and their values/locations in PM_2.5_ scale (second column). The lower table shows the curve trend for each region according to the thresholds listed in the upper table (from the second row to the bottom row). The Coef. (in column) and v1 (in row) refers to the slope of the curve in a region and the Coef. (in column) and cons (in row) refers to the intercept in that region.


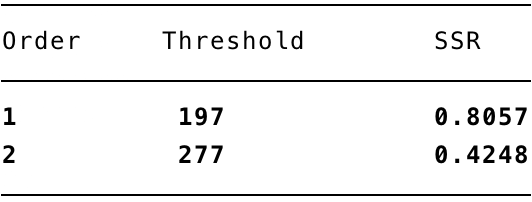

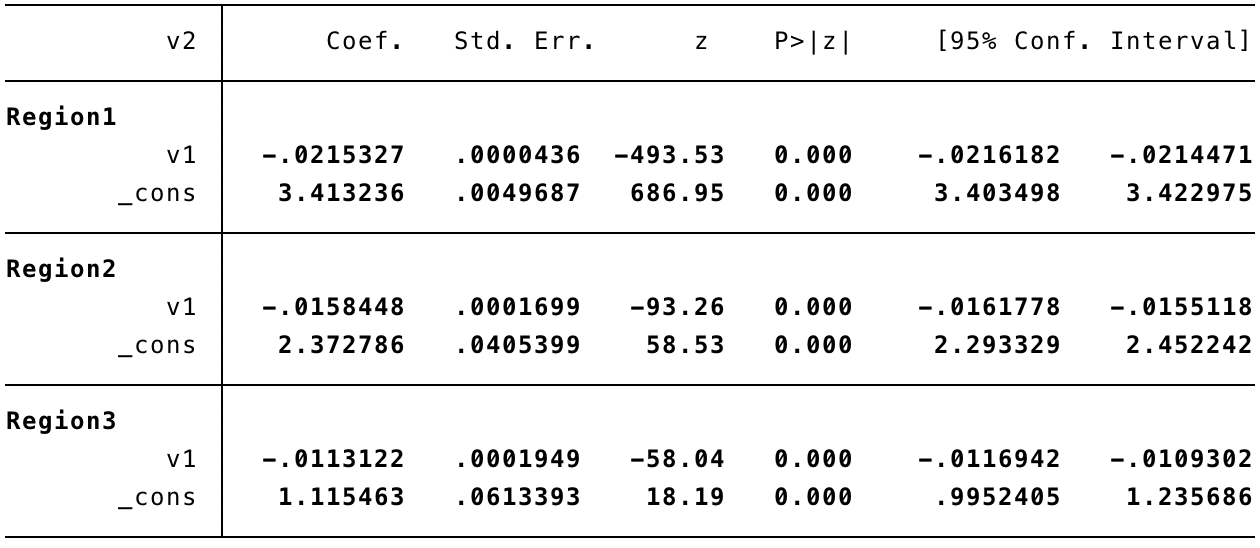


## Supplementary Table 29: Threshold Regression statistics For Figure2e (Less serious than smoking). The upper table shows the suggested thresholds including the order of significance (first and third column) and their values/locations in PM_2.5_ scale (second column). The lower table shows the curve trend for each region according to the thresholds listed in the upper table (from the second row to the bottom row). The Coef. (in column) and v1 (in row) refers to the slope of the curve in a region and the Coef. (in column) and cons (in row) refers to the intercept in that region.

|  | Above 4 hours | Between 3 and 4 hours | Under 3 hours |
| --- | --- | --- | --- |
| order of polynomial | 3 | 3 | 3 |
| coefficient 1 | 6.69E-08 | -4.92E-08 | -2.93E-08 |
| coefficient 2 | 1.11E-06 | 6.36E-05 | 5.26E-05 |
| coefficient 3 | -2.58E-02 | -0.0347 | -0.0331 |
| coefficient 4 | 3.6449 | 3.9032 | 3.8550 |
| degrees of freedom | 14 | 14 | 14 |
| norm of the residuals | 0.7959 | 0.5412 | 0.7273 |
| R-Squared | 0.9905 | 0.9956 | 0.9921 |

## Supplementary Table 30: Polynomial Regression statistics For Figure2f. This table shows the statistics information for the curve fittings in Figure 2f. The statistics includes order of polynomial, coefficients of models and degree of freedom, norm of the residuals and R squared. The columns refer to the outdoor time of observers.


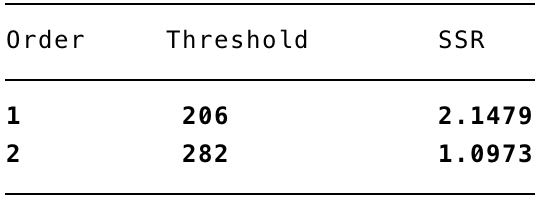

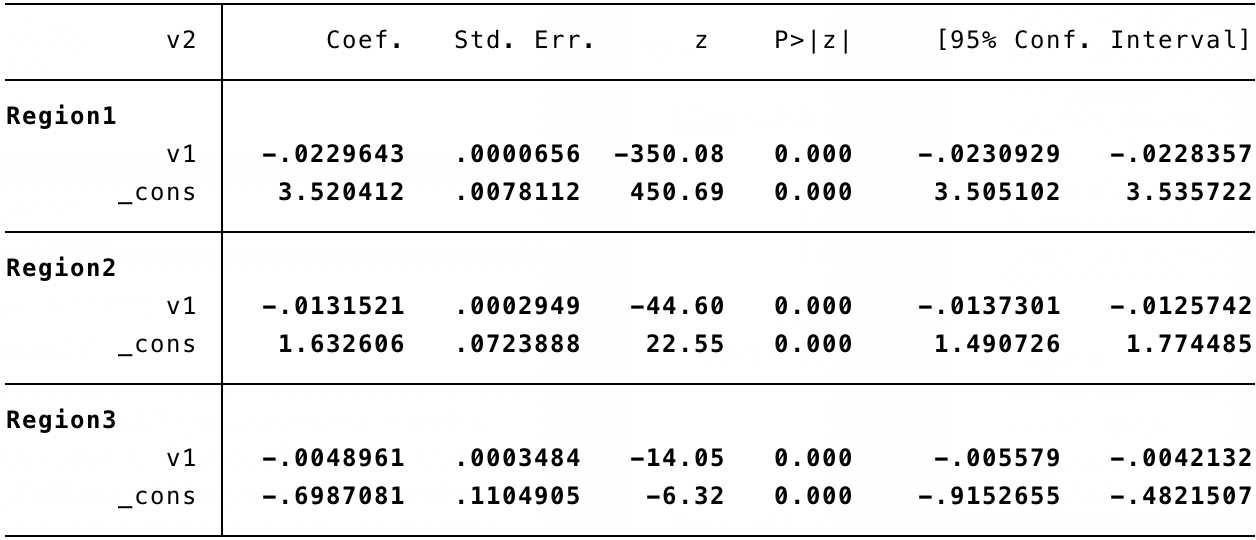


## Supplementary Table 31: Threshold Regression statistics For Figure2f (Above 4 hours). The upper table shows the suggested thresholds including the order of significance (first and third column) and their values/locations in PM_2.5_ scale (second column). The lower table shows the curve trend for each region according to the thresholds listed in the upper table (from the second row to the bottom row). The Coef. (in column) and v1 (in row) refers to the slope of the curve in a region and the Coef. (in column) and cons (in row) refers to the intercept in that region.


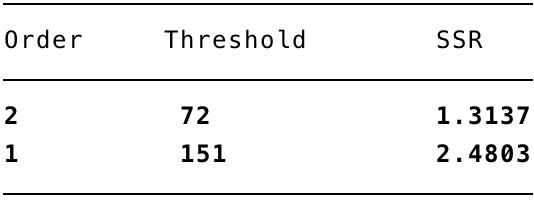

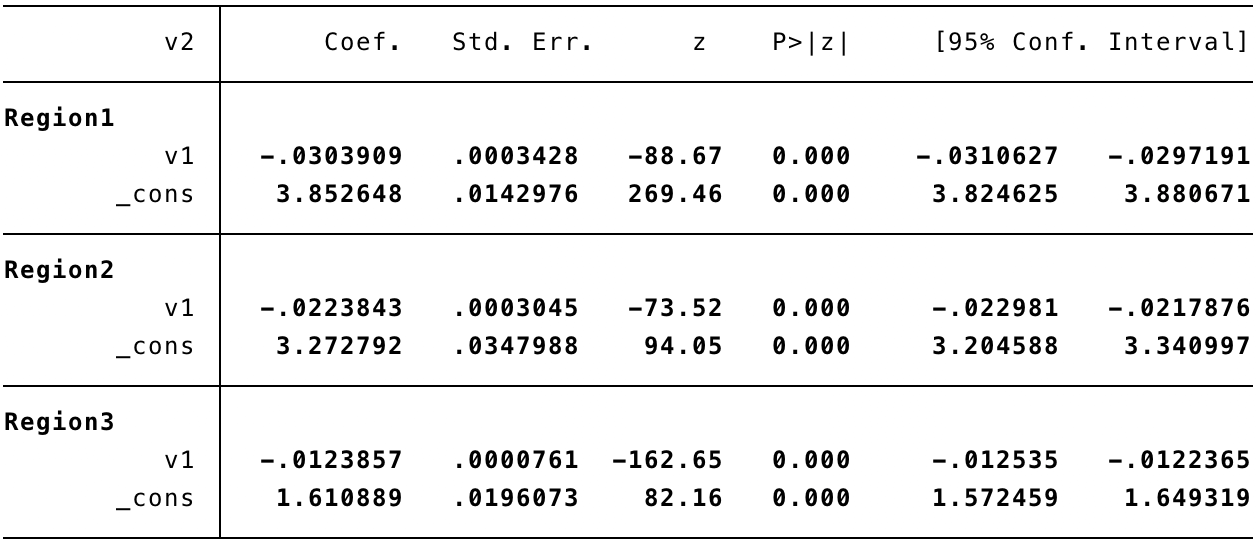


## Supplementary Table 32: Threshold Regression statistics For Figure2f (Between 3 and 4 hours). The upper table shows the suggested thresholds including the order of significance (first and third column) and their values/locations in PM_2.5_ scale (second column). The lower table shows the curve trend for each region according to the thresholds listed in the upper table (from the second row to the bottom row). The Coef. (in column) and v1 (in row) refers to the slope of the curve in a region and the Coef. (in column) and cons (in row) refers to the intercept in that region.


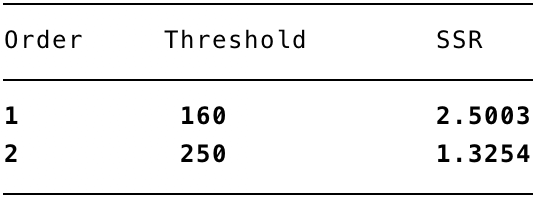

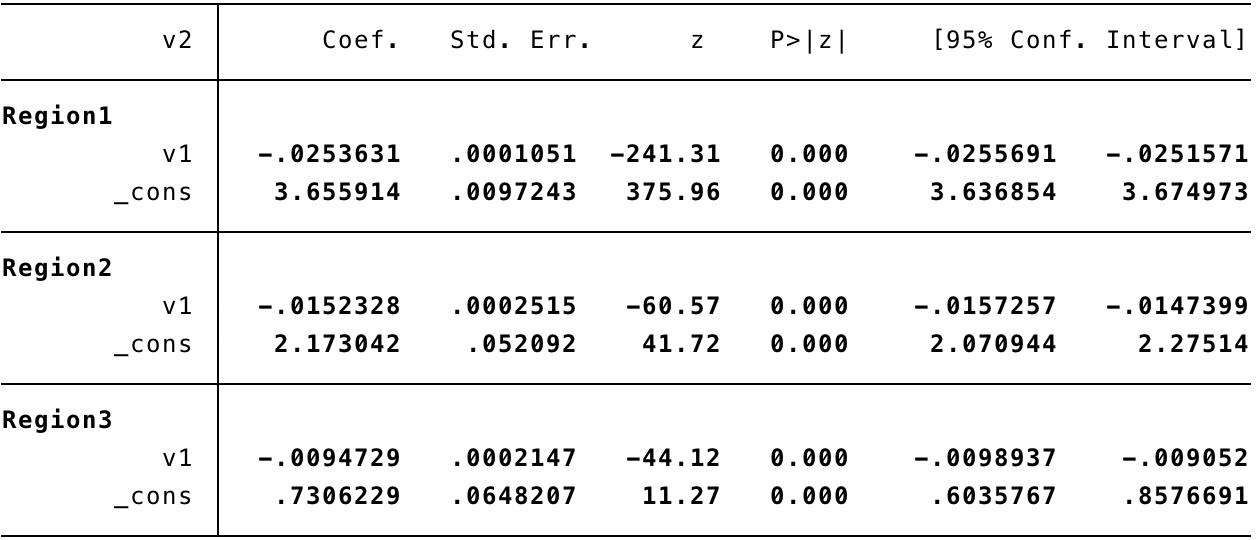


## Supplementary Table 33: Threshold Regression statistics For Figure2f (Under 3 hours). The upper table shows the suggested thresholds including the order of significance (first and third column) and their values/locations in PM_2.5_ scale (second column). The lower table shows the curve trend for each region according to the thresholds listed in the upper table (from the second row to the bottom row). The Coef. (in column) and v1 (in row) refers to the slope of the curve in a region and the Coef. (in column) and cons (in row) refers to the intercept in that region.

|  | Happiness China | Happiness UK |
| --- | --- | --- |
| order of polynomial | 3 | 3 |
| coefficient 1 | 2.62E-08 | -3.30E-08 |
| coefficient 2 | 1.10E-06 | 3.03E-05 |
| coefficient 3 | -0.0124 | -0.0161 |
| coefficient 4 | 4.2899 | 4.3870 |
| degrees of freedom | 14 | 14 |
| norm of the residuals | 0.6288 | 0.3757 |
| R-Squared | 0.9767 | 0.9917 |

## Supplementary Table 34: Polynomial Regression statistics For Figure3a (Happiness). This table show the statistics information for the fittings in Figure 3a. The statistics includes order of polynomial, coefficients of models and degree of freedom, norm of the residuals and R squared. The columns refer to the country background observers.


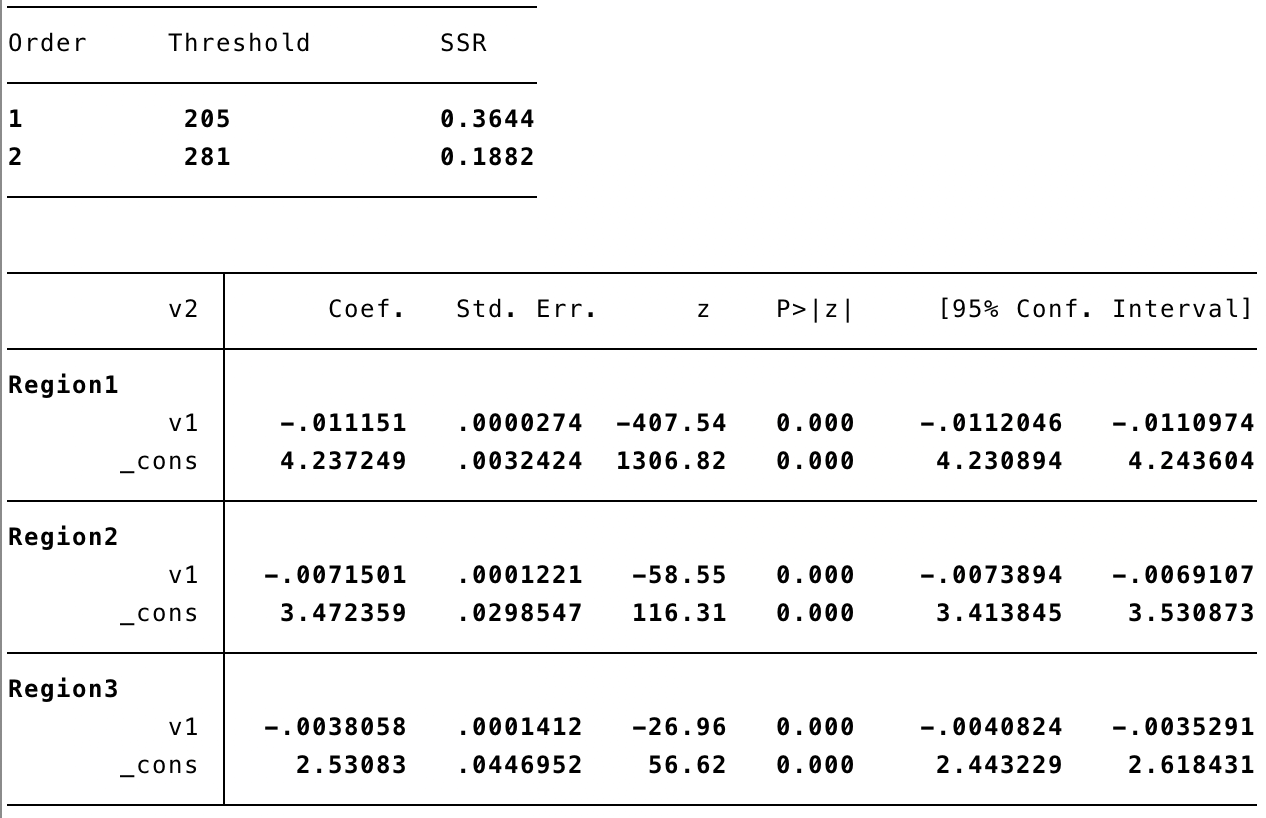


## Supplementary Table 35: Threshold Regression statistics For Figure3a (Happiness China). The upper table shows the suggested thresholds including the order of significance (first and third column) and their values/locations in PM_2.5_ scale (second column). The lower table shows the curve trend for each region according to the thresholds listed in the upper table (from the second row to the bottom row). The Coef. (in column) and v1 (in row) refers to the slope of the curve in a region and the Coef. (in column) and cons (in row) refers to the intercept in that region.


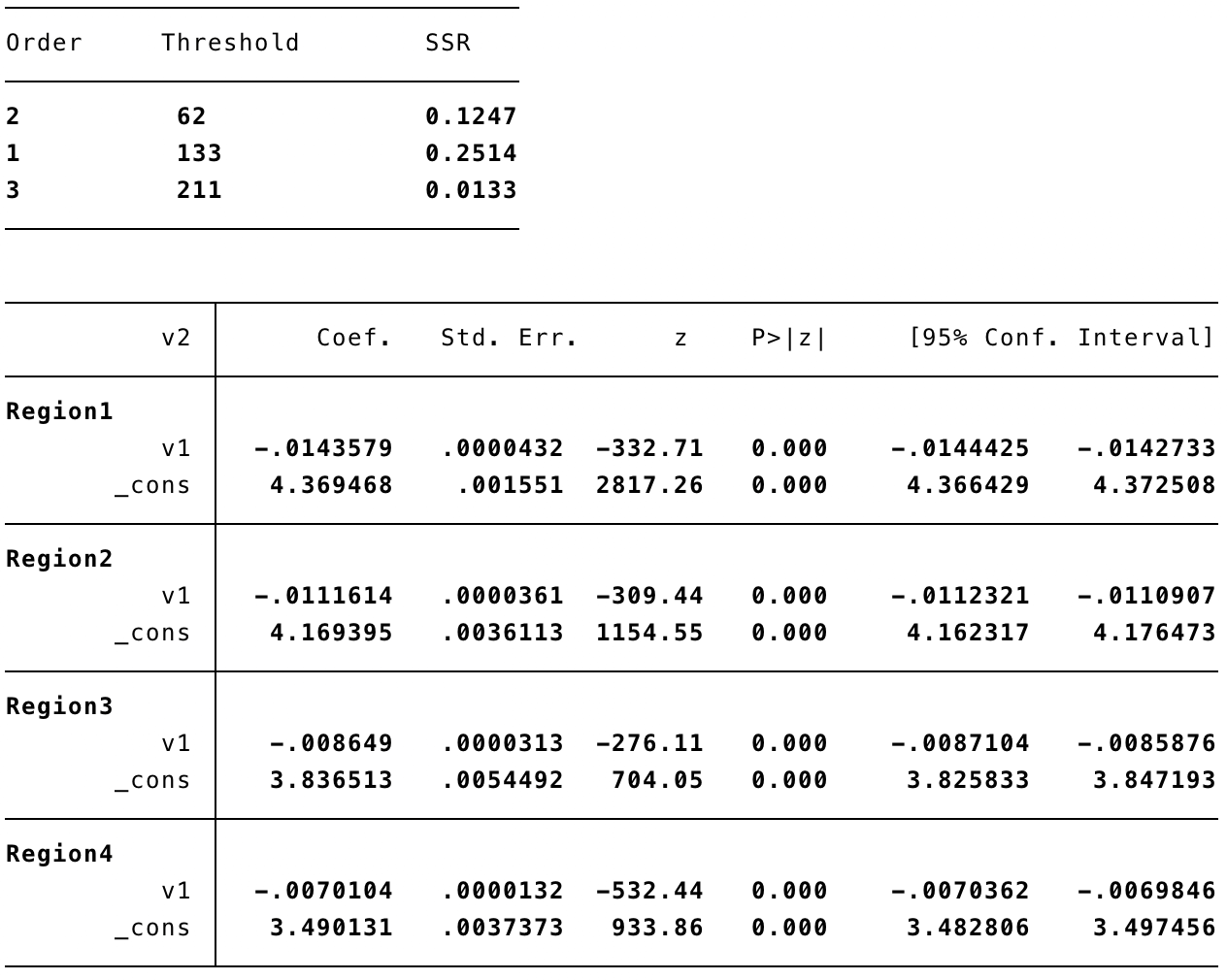


## Supplementary Table 36: Threshold Regression statistics For Figure3a (Happiness UK). The upper table shows the suggested thresholds including the order of significance (first and third column) and their values/locations in PM_2.5_ scale (second column). The lower table shows the curve trend for each region according to the thresholds listed in the upper table (from the second row to the bottom row). The Coef. (in column) and v1 (in row) refers to the slope of the curve in a region and the Coef. (in column) and cons (in row) refers to the intercept in that region.

|  | Future Expectations China | Future Expectations UK |
| --- | --- | --- |
| order of polynomial | 3 | 3 |
| coefficient 1 | 3.98E-08 | 7.40E-08 |
| coefficient 2 | 7.87E-06 | -2.78E-05 |
| coefficient 3 | -0.0158 | -0.00763959 |
| coefficient 4 | 4.4720 | 4.134537135 |
| degrees of freedom | 14 | 14 |
| norm of the residuals | 0.6690 | 0.5387 |
| R-Squared | 0.9737 | 0.9829 |

## Supplementary Table 37: Polynomial Regression statistics For Figure3b (Future Expectations). This table show the statistics information for the fittings in Figure 3b. The statistics includes order of polynomial, coefficients of models and degree of freedom, norm of the residuals and R squared. The columns refer to the country background observers.


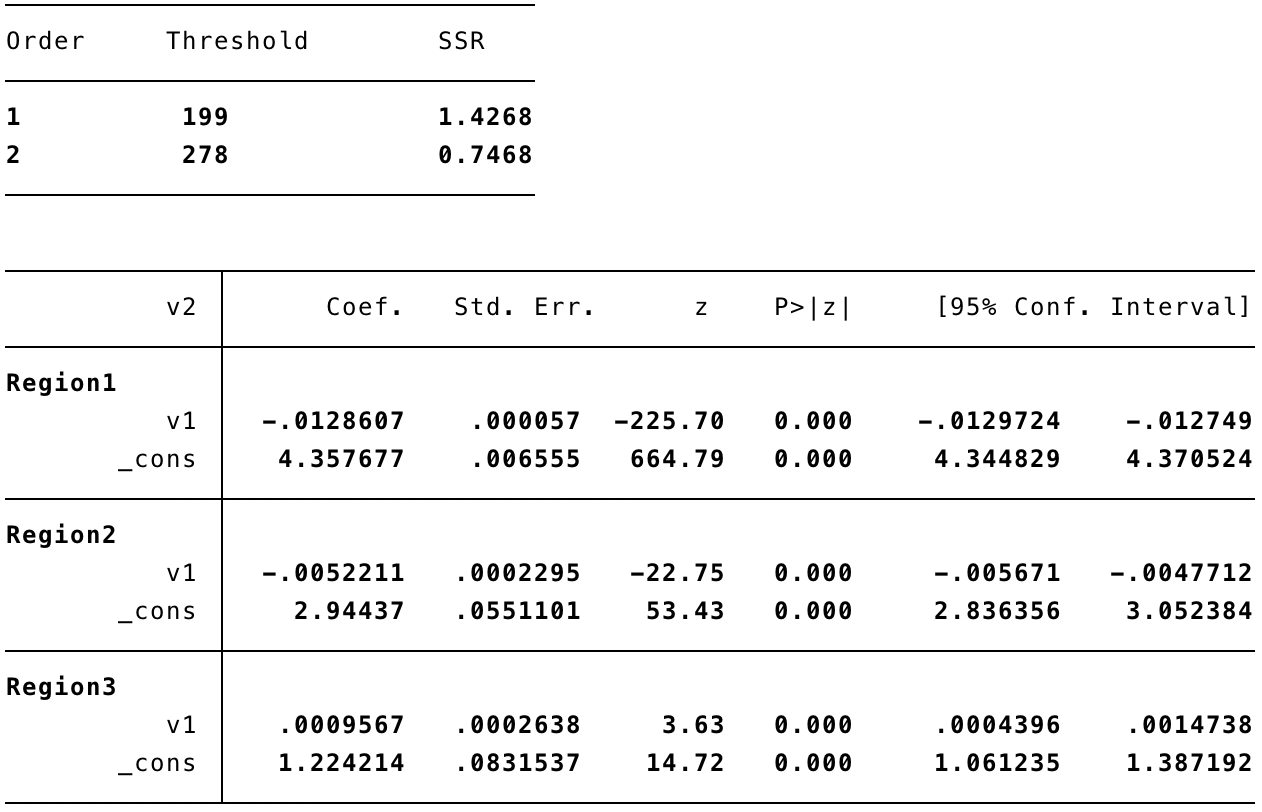


## Supplementary Table 38: Threshold Regression statistics For Figure3b (Future Expectations China). The upper table shows the suggested thresholds including the order of significance (first and third column) and their values/locations in PM_2.5_ scale (second column). The lower table shows the curve trend for each region according to the thresholds listed in the upper table (from the second row to the bottom row). The Coef. (in column) and v1 (in row) refers to the slope of the curve in a region and the Coef. (in column) and cons (in row) refers to the intercept in that region.

**
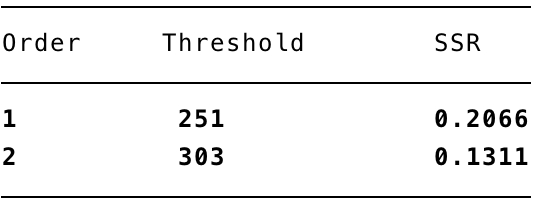

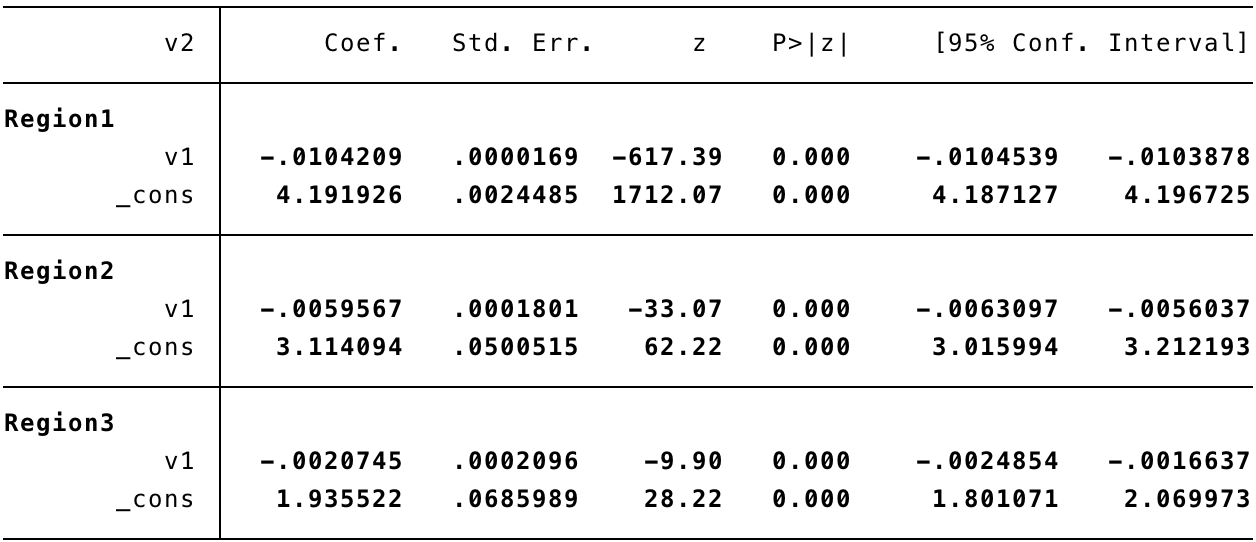
**

## Supplementary Table 39: Threshold Regression statistics For Figure3b (Future Expectations UK). The upper table shows the suggested thresholds including the order of significance (first and third column) and their values/locations in PM_2.5_ scale (second column). The lower table shows the curve trend for each region according to the thresholds listed in the upper table (from the second row to the bottom row). The Coef. (in column) and v1 (in row) refers to the slope of the curve in a region and the Coef. (in column) and cons (in row) refers to the intercept in that region.

|  | Worry China | Worry UK |
| --- | --- | --- |
| order of polynomial | 3 | 3 |
| coefficient 1 | -2.53E-08 | -2.08E-08 |
| coefficient 2 | -1.68E-06 | 4.01E-06 |
| coefficient 3 | 0.0125 | 0.0102 |
| coefficient 4 | 0.7060 | 0.8287 |
| degrees of freedom | 14 | 14 |
| norm of the residuals | 0.6170 | 0.5419 |
| R-Squared | 0.9776 | 0.9827 |

## Supplementary Table 40: Polynomial Regression statistics For Figure3c (Worry). This table show the statistics information for the fittings in Figure 3c. The statistics includes order of polynomial, coefficients of models and degree of freedom, norm of the residuals and R squared. The columns refer to the country background observers.


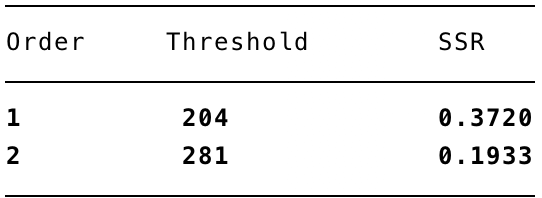

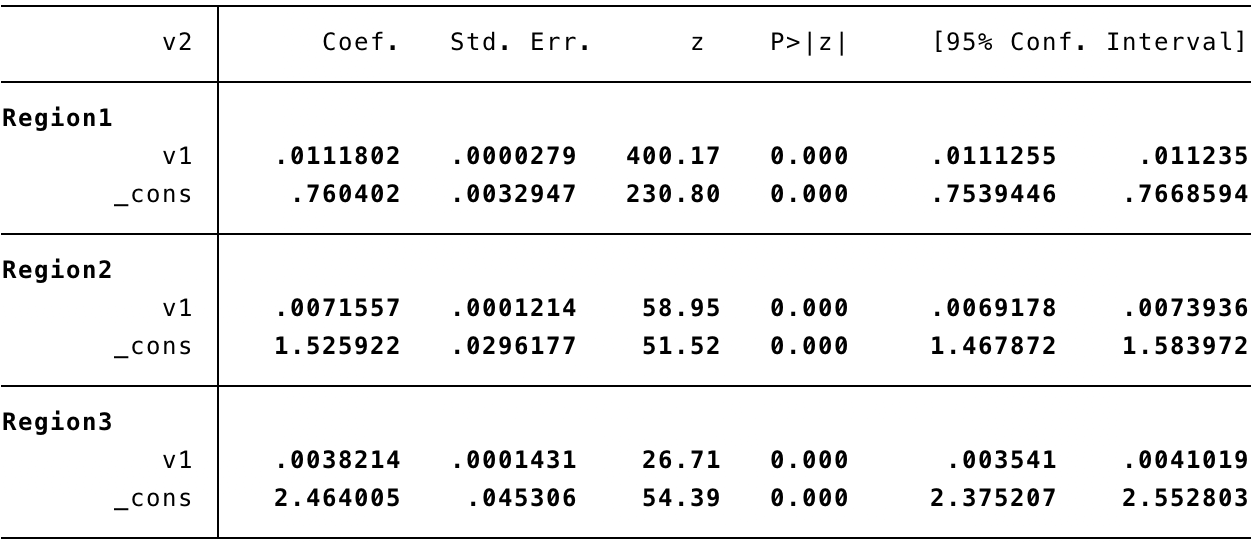


## Supplementary Table 41: Threshold Regression statistics For Figure3c (Worry China). The upper table shows the suggested thresholds including the order of significance (first and third column) and their values/locations in PM_2.5_ scale (second column). The lower table shows the curve trend for each region according to the thresholds listed in the upper table (from the second row to the bottom row). The Coef. (in column) and v1 (in row) refers to the slope of the curve in a region and the Coef. (in column) and cons (in row) refers to the intercept in that region.


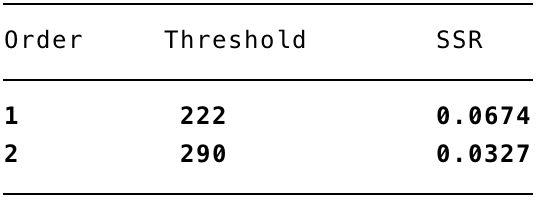

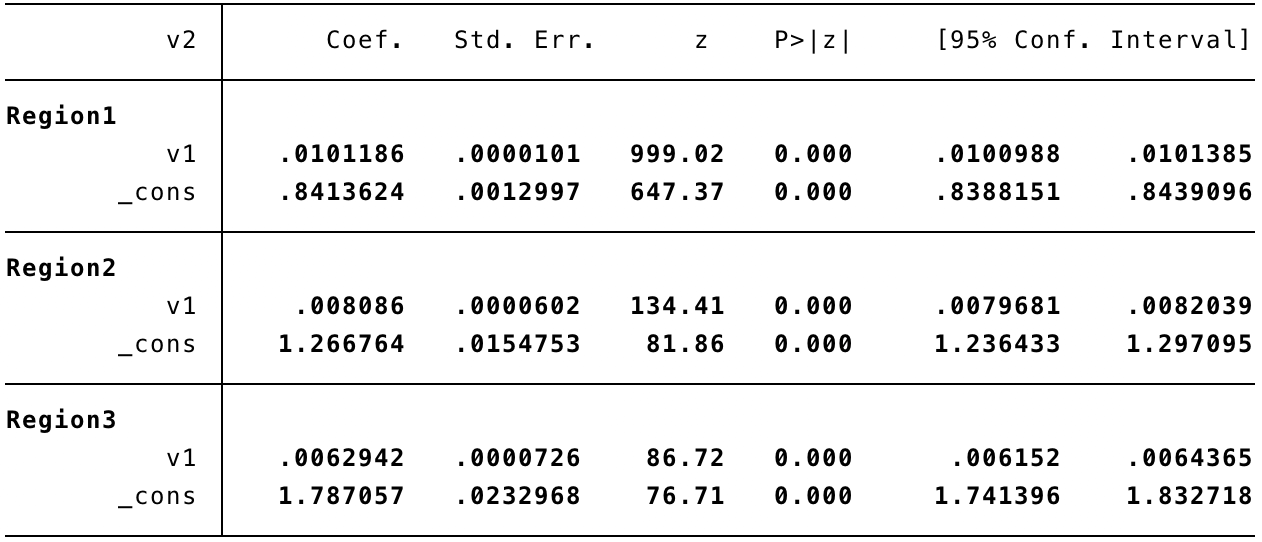


## Supplementary Table 42: Threshold Regression statistics For Figure3c (Worry UK). The upper table shows the suggested thresholds including the order of significance (first and third column) and their values/locations in PM_2.5_ scale (second column). The lower table shows the curve trend for each region according to the thresholds listed in the upper table (from the second row to the bottom row). The Coef. (in column) and v1 (in row) refers to the slope of the curve in a region and the Coef. (in column) and cons (in row) refers to the intercept in that region.

|  | Stress China | Stress UK |
| --- | --- | --- |
| order of polynomial | 3 | 3 |
| coefficient 1 | -7.28E-08 | 3.94E-08 |
| coefficient 2 | 1.96E-05 | -2.97E-05 |
| coefficient 3 | 0.0102 | 0.0153 |
| coefficient 4 | 0.7414 | 0.6697 |
| degrees of freedom | 14 | 14 |
| norm of the residuals | 0.5971 | 0.4544 |
| R-Squared | 0.9790 | 0.9879 |

## Supplementary Table 43: Polynomial Regression statistics For Figure3d (Stress). This table show the statistics information for the fittings in Figure 3d. The statistics includes order of polynomial, coefficients of models and degree of freedom, norm of the residuals and R squared. The columns refer to the country background observers.


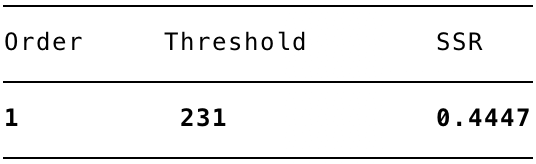

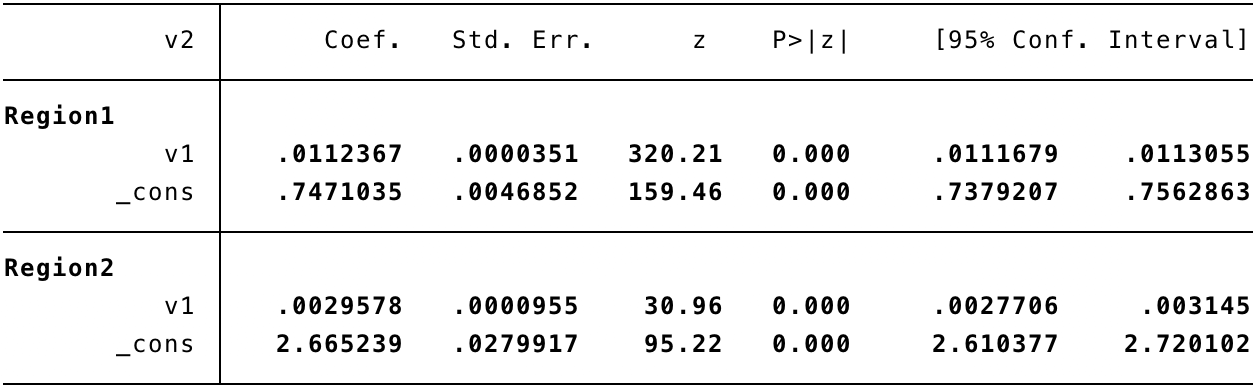


## Supplementary Table 44: Threshold Regression statistics For Figure3d (Stress China). The upper table shows the suggested thresholds including the order of significance (first and third column) and their values/locations in PM_2.5_ scale (second column). The lower table shows the curve trend for each region according to the thresholds listed in the upper table (from the second row to the bottom row). The Coef. (in column) and v1 (in row) refers to the slope of the curve in a region and the Coef. (in column) and cons (in row) refers to the intercept in that region.


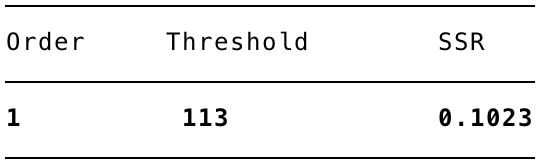

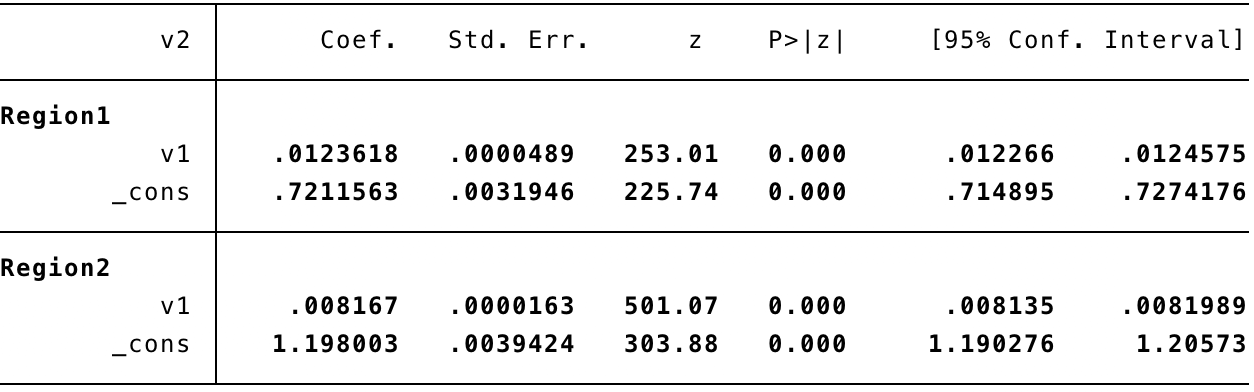


## Supplementary Table 45: Threshold Regression statistics For Figure3d (Stress UK). The upper table shows the suggested thresholds including the order of significance (first and third column) and their values/locations in PM_2.5_ scale (second column). The lower table shows the curve trend for each region according to the thresholds listed in the upper table (from the second row to the bottom row). The Coef. (in column) and v1 (in row) refers to the slope of the curve in a region and the Coef. (in column) and cons (in row) refers to the intercept in that region.

|  | Depression China | Depression UK |
| --- | --- | --- |
| order of polynomial | 3 | 3 |
| coefficient 1 | 7.84E-08 | 2.63E-08 |
| coefficient 2 | -6.36E-05 | -2.95E-05 |
| coefficient 3 | 0.0224 | 0.0164 |
| coefficient 4 | 0.3886 | 0.5991 |
| coefficient 5 | 14 | 14 |
| degrees of freedom | 0.5938 | 0.7263 |
| norm of the residuals | 0.9793 | 0.9690 |
| R-Squared | 3 | 3 |

## Supplementary Table 46: Polynomial Regression statistics For Figure3e (Depression). This table show the statistics information for the fittings in Figure 3d. The statistics includes order of polynomial, coefficients of models and degree of freedom, norm of the residuals and R squared. The columns refer to the country background observers.


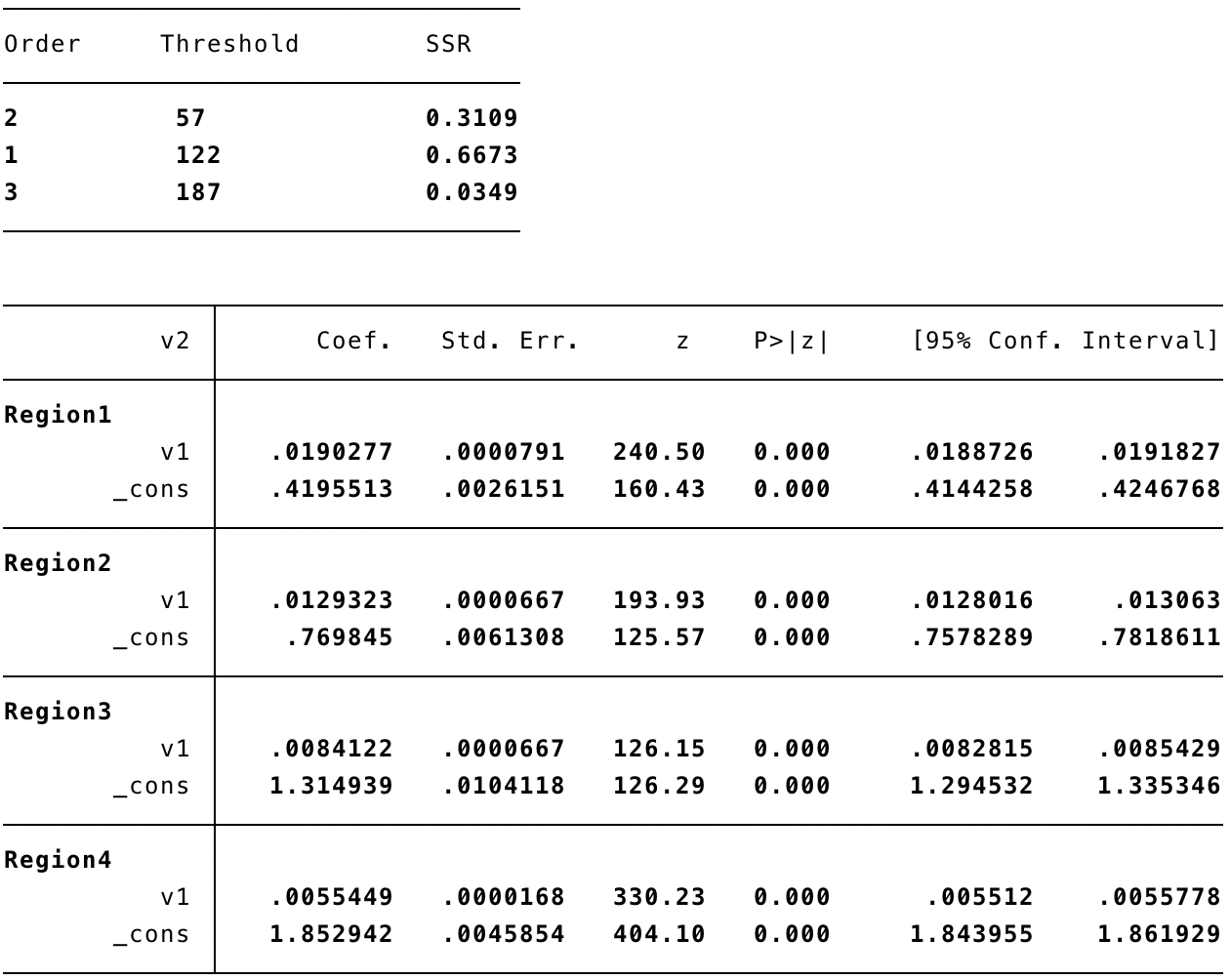


## Supplementary Table 47: Threshold Regression statistics For Figure3e (Depression China). The upper table shows the suggested thresholds including the order of significance (first and third column) and their values/locations in PM_2.5_ scale (second column). The lower table shows the curve trend for each region according to the thresholds listed in the upper table (from the second row to the bottom row). The Coef. (in column) and v1 (in row) refers to the slope of the curve in a region and the Coef. (in column) and cons (in row) refers to the intercept in that region.


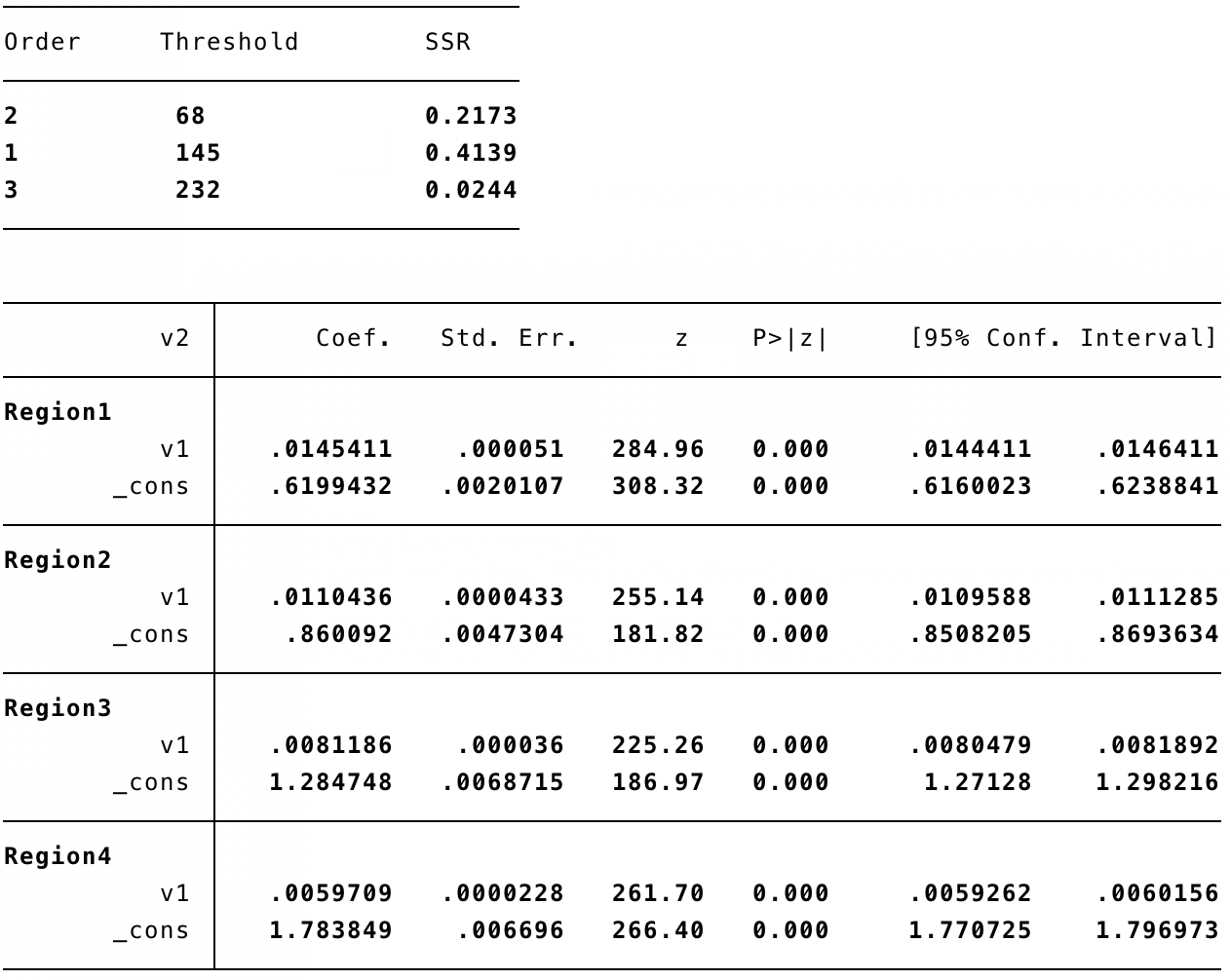


## Supplementary Table 48: Threshold Regression statistics For Figure3e (Depression UK). The upper table shows the suggested thresholds including the order of significance (first and third column) and their values/locations in PM_2.5_ scale (second column). The lower table shows the curve trend for each region according to the thresholds listed in the upper table (from the second row to the bottom row). The Coef. (in column) and v1 (in row) refers to the slope of the curve in a region and the Coef. (in column) and cons (in row) refers to the intercept in that region.

|  | Irritation China | Irritation UK |
| --- | --- | --- |
| order of polynomial | 3 | 3 |
| coefficient 1 | 6.69E-08 | 3.76E-08 |
| coefficient 2 | -5.94E-05 | -3.39E-05 |
| coefficient 3 | 0.0222 | 0.0168 |
| coefficient 4 | 0.3821 | 0.5937 |
| degrees of freedom | 14 | 14 |
| norm of the residuals | 0.5775 | 0.5658 |
| R-Squared | 0.9804 | 0.9812 |

## Supplementary Table 49: Polynomial Regression statistics For Figure3f (Irritation). This table show the statistics information for the fittings in Figure 3f. The statistics includes order of polynomial, coefficients of models and degree of freedom, norm of the residuals and R squared. The columns refer to the country background observers.


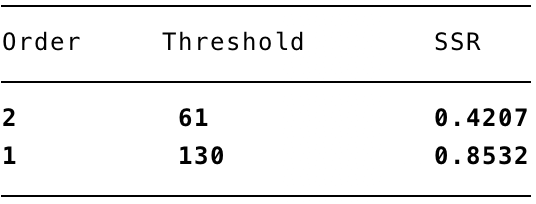

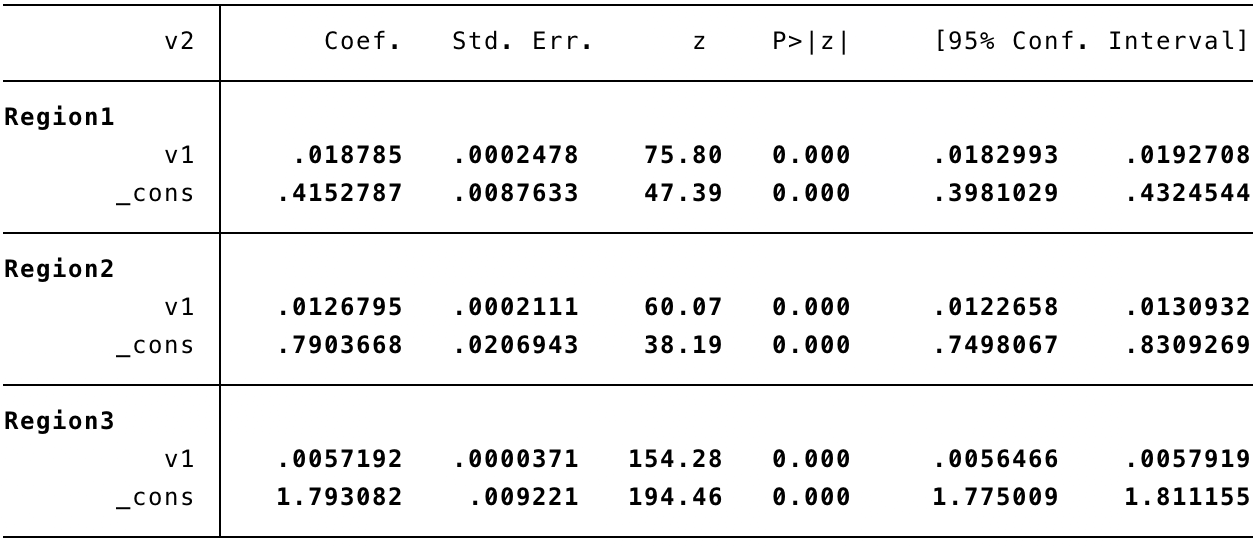


## Supplementary Table 50: Threshold Regression statistics For Figure3f (Irritation China). The upper table shows the suggested thresholds including the order of significance (first and third column) and their values/locations in PM_2.5_ scale (second column). The lower table shows the curve trend for each region according to the thresholds listed in the upper table (from the second row to the bottom row). The Coef. (in column) and v1 (in row) refers to the slope of the curve in a region and the Coef. (in column) and cons (in row) refers to the intercept in that region.


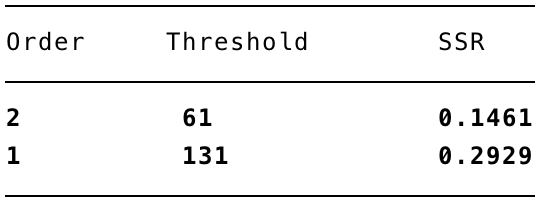

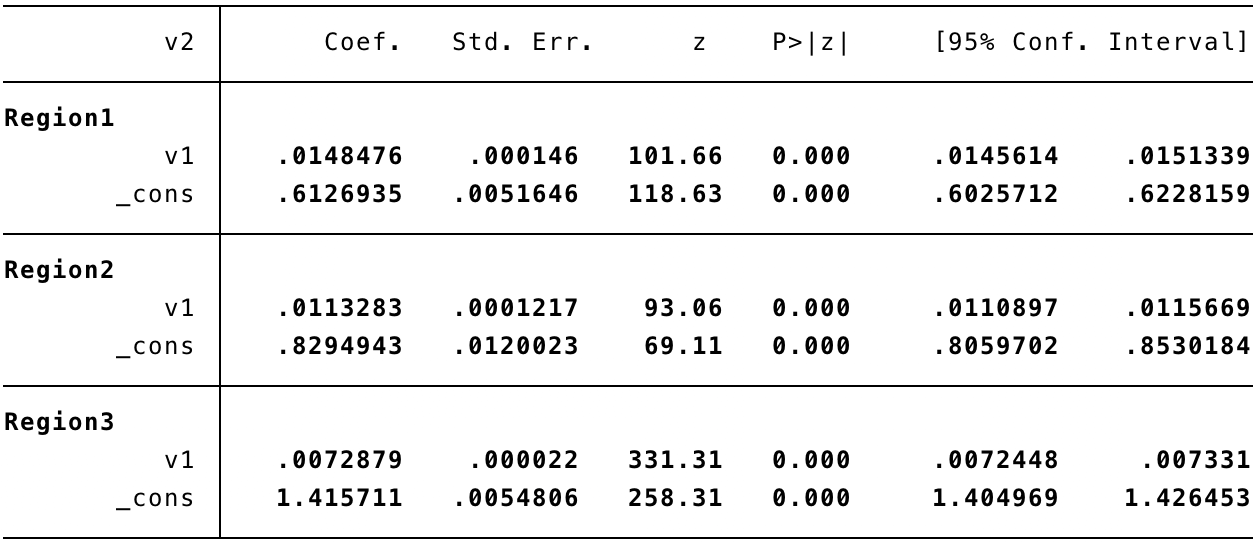


## Supplementary Table 51: Threshold Regression statistics For Figure3f (Irritation UK). The upper table shows the suggested thresholds including the order of significance (first and third column) and their values/locations in PM_2.5_ scale (second column). The lower table shows the curve trend for each region according to the thresholds listed in the upper table (from the second row to the bottom row). The Coef. (in column) and v1 (in row) refers to the slope of the curve in a region and the Coef. (in column) and cons (in row) refers to the intercept in that region.

|  | SWB China | SWB UK |
| --- | --- | --- |
| order of polynomial | 3 | 3 |
| coefficient 1 | 2.12E-08 | -1.22E-10 |
| coefficient 2 | 3.07E-05 | 2.35E-05 |
| coefficient 3 | -0.0309 | -0.02654208 |
| coefficient 4 | 3.8264 | 3.587933603 |
| degrees of freedom | 14 | 14 |
| norm of the residuals | 0.8429 | 0.5768 |
| R-Squared | 0.9894 | 0.9950 |

## Supplementary Table 52: Polynomial Regression statistics For Figure3g (SWB). This table show the statistics information for the fittings in Figure 3g. The statistics includes order of polynomial, coefficients of models and degree of freedom, norm of the residuals and R squared. The columns refer to the country background observers.


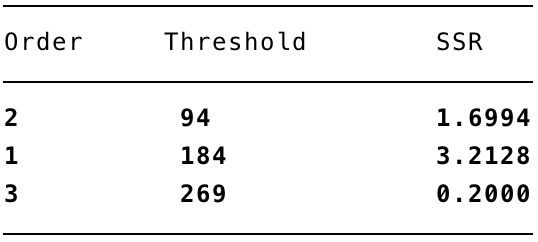

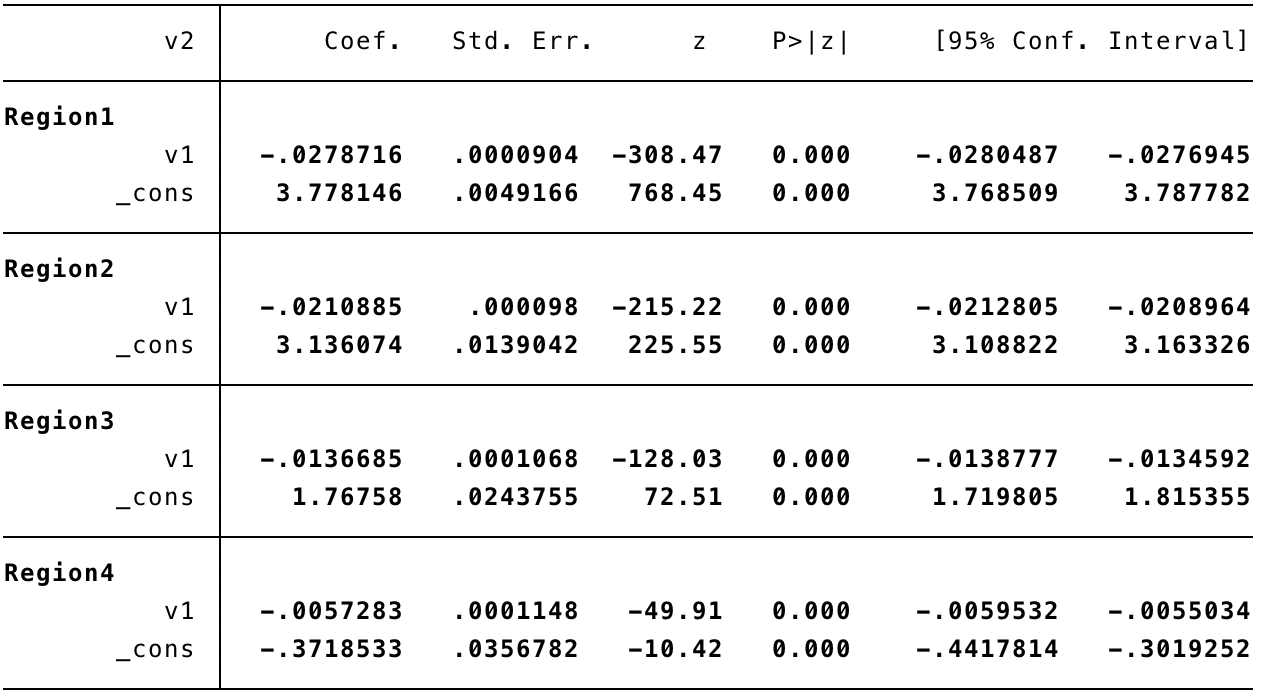


## Supplementary Table 53: Threshold Regression statistics For Figure3g (SWB China). The upper table shows the suggested thresholds including the order of significance (first and third column) and their values/locations in PM_2.5_ scale (second column). The lower table shows the curve trend for each region according to the thresholds listed in the upper table (from the second row to the bottom row). The Coef. (in column) and v1 (in row) refers to the slope of the curve in a region and the Coef. (in column) and cons (in row) refers to the intercept in that region.


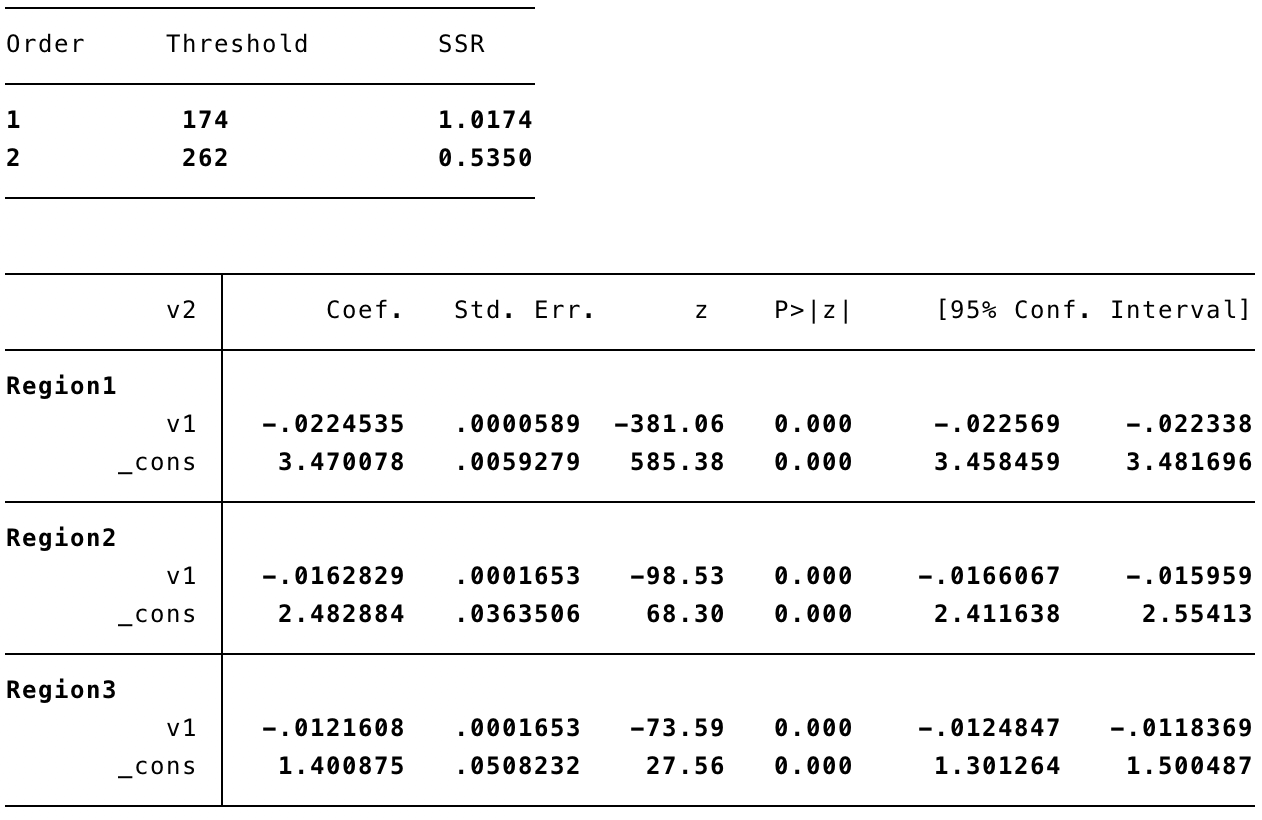


## Supplementary Table 54: Threshold Regression statistics For Figure3g (SWB UK). The upper table shows the suggested thresholds including the order of significance (first and third column) and their values/locations in PM_2.5_ scale (second column). The lower table shows the curve trend for each region according to the thresholds listed in the upper table (from the second row to the bottom row). The Coef. (in column) and v1 (in row) refers to the slope of the curve in a region and the Coef. (in column) and cons (in row) refers to the intercept in that region.

|  | category1 | category2 | category3 | category4 | category5 | category6 | category7 |
| --- | --- | --- | --- | --- | --- | --- | --- |
| image1 | 0 | 0 | 1 | 4 | 8 | 32 | 34 |
| image2 | 0 | 1 | 2 | 5 | 20 | 31 | 20 |
| image3 | 0 | 0 | 4 | 10 | 23 | 27 | 15 |
| image4 | 0 | 0 | 6 | 17 | 30 | 20 | 6 |
| image5 | 0 | 3 | 7 | 25 | 32 | 11 | 1 |
| image6 | 0 | 8 | 9 | 24 | 30 | 8 | 0 |
| image7 | 0 | 8 | 17 | 26 | 22 | 5 | 1 |
| image8 | 0 | 10 | 20 | 31 | 16 | 2 | 0 |
| image9 | 2 | 10 | 27 | 29 | 8 | 3 | 0 |
| image10 | 1 | 23 | 34 | 15 | 6 | 0 | 0 |
| image11 | 5 | 25 | 30 | 12 | 6 | 1 | 0 |
| image12 | 9 | 25 | 26 | 16 | 3 | 0 | 0 |
| image13 | 12 | 31 | 24 | 9 | 3 | 0 | 0 |
| image14 | 21 | 36 | 14 | 6 | 2 | 0 | 0 |
| image15 | 32 | 21 | 20 | 4 | 2 | 0 | 0 |
| image16 | 27 | 38 | 10 | 3 | 1 | 0 | 0 |
| image17 | 42 | 28 | 6 | 3 | 0 | 0 | 0 |
| image18 | 53 | 20 | 4 | 2 | 0 | 0 | 0 |

## Supplementary Table 55: Frequency Matrix F for Happiness. The column refers to happiness categories. The row refers to air pollution level. This table shows the frequency that each air pollution level was judged to be belonged to a certain happiness category by all observers.

|  | category1 | category2 | category3 | category4 | category5 | category6 | category7 |
| --- | --- | --- | --- | --- | --- | --- | --- |
| image1 | 0 | 0 | 1 | 5 | 13 | 45 | 79 |
| image2 | 0 | 1 | 3 | 8 | 28 | 59 | 79 |
| image3 | 0 | 0 | 4 | 14 | 37 | 64 | 79 |
| image4 | 0 | 0 | 6 | 23 | 53 | 73 | 79 |
| image5 | 0 | 3 | 10 | 35 | 67 | 78 | 79 |
| image6 | 0 | 8 | 17 | 41 | 71 | 79 | 79 |
| image7 | 0 | 8 | 25 | 51 | 73 | 78 | 79 |
| image8 | 0 | 10 | 30 | 61 | 77 | 79 | 79 |
| image9 | 2 | 12 | 39 | 68 | 76 | 79 | 79 |
| image10 | 1 | 24 | 58 | 73 | 79 | 79 | 79 |
| image11 | 5 | 30 | 60 | 72 | 78 | 79 | 79 |
| image12 | 9 | 34 | 60 | 76 | 79 | 79 | 79 |
| image13 | 12 | 43 | 67 | 76 | 79 | 79 | 79 |
| image14 | 21 | 57 | 71 | 77 | 79 | 79 | 79 |
| image15 | 32 | 53 | 73 | 77 | 79 | 79 | 79 |
| image16 | 27 | 65 | 75 | 78 | 79 | 79 | 79 |
| image17 | 42 | 70 | 76 | 79 | 79 | 79 | 79 |
| image18 | 53 | 73 | 77 | 79 | 79 | 79 | 79 |

## Supplementary Table 56: Cumulative Frequency Matrix Φ For Happiness. The Matrix Φ can be determined by calculating from the left from Matrix F. The element in the jth row and the gth column is the number of times that image j was sorted below category g.

|  | category1 | category2 | category3 | category4 | category5 | category6 | category7 |
| --- | --- | --- | --- | --- | --- | --- | --- |
| image1 | 0.00 | 0.00 | 0.01 | 0.06 | 0.16 | 0.57 | 1.00 |
| image2 | 0.00 | 0.01 | 0.04 | 0.10 | 0.35 | 0.75 | 1.00 |
| image3 | 0.00 | 0.00 | 0.05 | 0.18 | 0.47 | 0.81 | 1.00 |
| image4 | 0.00 | 0.00 | 0.08 | 0.29 | 0.67 | 0.92 | 1.00 |
| image5 | 0.00 | 0.04 | 0.13 | 0.44 | 0.85 | 0.99 | 1.00 |
| image6 | 0.00 | 0.10 | 0.22 | 0.52 | 0.90 | 1.00 | 1.00 |
| image7 | 0.00 | 0.10 | 0.32 | 0.65 | 0.92 | 0.99 | 1.00 |
| image8 | 0.00 | 0.13 | 0.38 | 0.77 | 0.97 | 1.00 | 1.00 |
| image9 | 0.03 | 0.15 | 0.49 | 0.86 | 0.96 | 1.00 | 1.00 |
| image10 | 0.01 | 0.30 | 0.73 | 0.92 | 1.00 | 1.00 | 1.00 |
| image11 | 0.06 | 0.38 | 0.76 | 0.91 | 0.99 | 1.00 | 1.00 |
| image12 | 0.11 | 0.43 | 0.76 | 0.96 | 1.00 | 1.00 | 1.00 |
| image13 | 0.15 | 0.54 | 0.85 | 0.96 | 1.00 | 1.00 | 1.00 |
| image14 | 0.27 | 0.72 | 0.90 | 0.97 | 1.00 | 1.00 | 1.00 |
| image15 | 0.41 | 0.67 | 0.92 | 0.97 | 1.00 | 1.00 | 1.00 |
| image16 | 0.34 | 0.82 | 0.95 | 0.99 | 1.00 | 1.00 | 1.00 |
| image17 | 0.53 | 0.89 | 0.96 | 1.00 | 1.00 | 1.00 | 1.00 |
| image18 | 0.67 | 0.92 | 0.97 | 1.00 | 1.00 | 1.00 | 1.00 |

## Supplementary Table 57: Proportion Matrix P for Happiness. Matrix P is derived from matrix Φ. The element in matrix P shows the proportion of times that image j is sorted below the gth category. So the P matrix can be obtained by dividing each element in matrix Φ by 79.

|  | category1 | category2 | category3 | category4 | category5 | category6 |
| --- | --- | --- | --- | --- | --- | --- |
| image1 | -4.26 | -4.26 | -2.24 | -1.53 | -0.98 | 0.18 |
| image2 | -4.26 | -2.24 | -1.77 | -1.27 | -0.37 | 0.66 |
| image3 | -4.26 | -4.26 | -1.64 | -0.93 | -0.08 | 0.88 |
| image4 | -4.26 | -4.26 | -1.43 | -0.55 | 0.44 | 1.43 |
| image5 | -4.26 | -1.77 | -1.14 | -0.14 | 1.03 | 2.24 |
| image6 | -4.26 | -1.27 | -0.79 | 0.05 | 1.27 | 4.26 |
| image7 | -4.26 | -1.27 | -0.48 | 0.37 | 1.43 | 2.24 |
| image8 | -4.26 | -1.14 | -0.31 | 0.75 | 1.95 | 4.26 |
| image9 | -1.95 | -1.03 | -0.02 | 1.08 | 1.77 | 4.26 |
| image10 | -2.24 | -0.51 | 0.63 | 1.43 | 4.26 | 4.26 |
| image11 | -1.53 | -0.31 | 0.70 | 1.35 | 2.24 | 4.26 |
| image12 | -1.21 | -0.18 | 0.70 | 1.77 | 4.26 | 4.26 |
| image13 | -1.03 | 0.11 | 1.03 | 1.77 | 4.26 | 4.26 |
| image14 | -0.63 | 0.59 | 1.27 | 1.95 | 4.26 | 4.26 |
| image15 | -0.24 | 0.44 | 1.43 | 1.95 | 4.26 | 4.26 |
| image16 | -0.41 | 0.93 | 1.64 | 2.24 | 4.26 | 4.26 |
| image17 | 0.08 | 1.21 | 1.77 | 4.26 | 4.26 | 4.26 |
| image18 | 0.44 | 1.43 | 1.95 | 4.26 | 4.26 | 4.26 |

## Supplementary Table 58: Normal Deviate Matrix X for Happiness. Matrix X is a basic transformation matrix whose elements are the unit in normal deviates corresponding to elements in matrix P.

| image | 1 | 2 | 3 | 4 | 5 | 6 | 7 | 8 | 9 | 10 | 11 | 12 | 13 | 14 | 15 | 16 | 17 | 18 |
| --- | --- | --- | --- | --- | --- | --- | --- | --- | --- | --- | --- | --- | --- | --- | --- | --- | --- | --- |
| s scale value | 2.75 | 2.11 | 2.28 | 2.01 | 1.24 | 0.69 | 0.90 | 0.36 | -0.12 | -0.74 | -0.55 | -1.04 | -1.17 | -1.39 | -1.45 | -1.59 | -2.08 | -2.20 |
| z z-score | 4.22 | 3.82 | 3.93 | 3.76 | 3.28 | 2.93 | 3.06 | 2.72 | 2.42 | 2.04 | 2.15 | 1.85 | 1.77 | 1.63 | 1.59 | 1.50 | 1.20 | 1.12 |

## Supplementary Table 59: Scale values and z-scores of Happiness. The column refers to each 18 air levels. The column shows the s scale values for each air pollution image based on Supplementary Equation 1 and the standardbred z-scores calculated based on Equation 3 in main text.

# Supplementary Note 1

## Air Pollution and Climate Data Range for Building Air Pollution and Colour Information Model M1

In our database, PM2.5 varied from 17 to 399, ozone varied from 2 to 32, NO2 varied from 5 to 38, SO2 varied from 2 to 50, and CO varied from 2 to 60. Temperature varies from -16 to 15 Celsius degree, wind speed varies from 1 to 13 m/s, humidity covers from 1 to 100, SL pressure covers from 1008.5 to 1043.4, wind direction covers from 0 to 1in 360 degrees, clouds cover covers from 0 to 1, time duration from sunset covers from 32 to 310 minutes.

## Camera Position and Photos Selection

The distance between the camera and the US embassy monitoring station was approximately 5.86 km. Totally 111 photos were selected in this study. The standard for selecting photos aimed to obtain the air pollution data averagely covering the investigated air pollution range.

## Analytical Procedure of Categorical Judgement

Psychophysical scaling method is applied to generate a relationship between physical stimuli and mental or perceptual magnitudes. Torgerson’s law of categorical judgement is developed from Thurstone’s law of comparative judgement with different assumptions. Firstly, the psychophysical continuum of the investigated attribute can be divided into a given number of ordered categories. Secondly, because of various factors, the location of a given category does not always lie on the same place in the continuum but forms a normal distribution and different categories may have different mean locations and different standard deviations. Thirdly, an observer judges a given stimulus to be below a given category boundary whenever the value of the category boundary on the continuum is more than that of the stimulus.

The categorical judgement analytical method used in this study is the standard deviation of each category boundary (7 categories in total) *σ_g_*, the standard deviation of each simulated image (18 images in total) *σ_j_* and the correlation coefficient between the momentary positions (of pollution level) of simulated image and category boundary *r_jg_* are all constants^1-3^. This particular method is referred as ‘condition D’ in the textbook of Theory and methods of scaling, such as Torgerson^3^.

Equation 2 in the main-text is a standard model for categorical judgement. Under the special ‘condition D’, the Supplementary Equation 1 can be simplified as below:


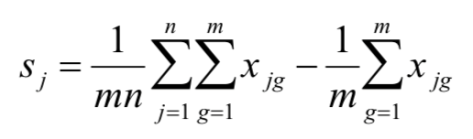


Where *s_j_* is the scale value of simulated image *j*;

*n* is the number of stimuli (*n* =18);

*m* is the number of categories minus 1 (*m*=7-1);

*x_jg_* is the unit normal deviate of stimulus *j* in the *g*th category.

Take happiness as an example. Several basic matrices for processing scale values *s_j_* of happiness in the law of categorical judgement are presented from frequency Matrix **F** to normal deviate Matrix **X**. The values of *x_jg_* can be located in Matrix **X**.

According to the raw data from the experiment where each of 79 observers judged 18 air quality images according to their happiness standard and sorted each of the 18 images into one of 7 categories. The frequency Matrix **F** can be generated as in Supplementary Table 55. From Matrix **F**, cumulative frequency matrix **Φ** (in Supplementary Table 56) can be determined by calculating each element in Matrix **F** from the left. The element in the *j*th row and the *g*th column is the number of times that stimulus (image) j was sorted below category *g*.

Proportion Matrix **P** (in Supplementary Table 57) is derived from matrix **Φ**. The element in matrix **P** shows the proportion of times that stimulus (image) *j* is sorted below the *g*th category. In the illustrated example, there are a total 79 judgements, so the **P** matrix can be obtained by dividing each element in matrix **Φ** by 79.

Normal Deviate Matrix **X** (in Supplementary Table 58) is a basic transformation matrix whose elements are the unit in normal deviates corresponding to elements in matrix **P**. Compared with matrix **X**, the last column is removed as the unit normal deviate of 100 percent is not a number. The value of *x_jg_* can be located from matrix **X**.

According to Supplementary Equation 1 and *x_jg_* in matrix **X**. The happiness scale values *s* of 18 images can be calculated. Based on Equation 3 in main text. The standardized scale values z-score can be calculated in the Supplementary Table 59 so that different emotional scale values can be compared and further calculated. All the emotional and SWB values used in analysis are all processed z-score values.

$\boldsymbol{Z}_{\boldsymbol{sjf}}\boldsymbol{=}\frac{\boldsymbol{s}_{\boldsymbol{jf}}\boldsymbol{-}{\bar{\boldsymbol{s}}}_{\boldsymbol{f}}}{\boldsymbol{\sigma}_{\boldsymbol{f}}}$ + 5 Equation 3

where *Z_sjf_* is the standardized z-score of scale value *s* of the simulated image *j* of the emotion *f*;

*s_jf_* is the scale value of the simulated image *j* of emotion *f*;

$\bar{s}_{\boldsymbol{f}}$ is the mean of variable *s* of the emotion *f*;

*σ_g_* is the standard deviation of variable *s* of the emotion *f*.

# Supplementary Note 2

## Influencing from age on SWB under air pollution between China and UK

In Supplementary Figure 8, the effect of age on SWB under air pollution between Chinese and British people is compared. Supplementary Figure8a and b show the comparison of SWB under air pollution between Chinese (red) and British (blue) in 2 age groups. Supplementary Figure8a is for under 31 and Supplementary Figure8b is for people above 30. It can be found from Supplementary Figure8a that there is not much difference in SWB between Chinese and British younger people when the air condition is fairly good. However, as the deterioration of air condition exceeds 300 AQI, the Chinese younger people numb to serious air pollution earlier than the British younger people. Supplementary Figure8b shows that the difference between 2 countries is more obvious. The Chinese older people have stronger positive feelings about good air condition than British older people and they become less sensitive to deterioration of serious air pollution earlier too. Therefore, it can be found that the country background makes more SWB difference in older people under air condition changes. Supplementary Figure8c and Supplementary Figure8d show the difference of age influence on SWB between two countries. Supplementary Figure8c indicates that in China the older people’s SWB are more sensitive than younger people’s in air condition changes. Obvious gaps can be observed in the figure as the air quality changes. However, Supplementary Figure8d indicates that there is not much difference between British younger and older people’s SWB, especially when the air condition is fairly good. This comparison indicates that the SWB of people who have experienced serious air pollution like Chinese is more sensitive to air condition changes and among the experienced people the older ones are even more sensitive than the younger ones. For those who have no experience of serious air pollution like the British, the factor of age does not make too much difference on SWB under different air conditions.

## Influencing from gender on SWB under air pollution between China and UK

Supplementary Figure 9 shows the gender influence on SWB under air pollution changes between China and the UK. Supplementary Figure9a and Supplementary Figure9b show the comparisons between Chinese and British in male and female groups separately. The results agree with one conclusion in the main information (Figure 3g) very well, that both the Chinese male and female are more sensitive than the British gender groups. Supplementary Figure9c and d shows the gender influence on SWB for difference countries. It can be found in Supplementary Figure9c, that there is no obvious difference between Chinese male’ and female’s SWB under different air conditions. However, in Supplementary Figure9d, the SWB of British female is clearly and constantly higher than British male until the air quality index increases to about 80 AQI and after that it reduces faster than the SWB of British male. This indicates British females are more sensitive than males under fairly good air quality. As a result of the limitation of our air quality scale it does not show an obvious difference between British female and male in the figure when the air quality getting serious. According to Supplementary Figure9, it can be concluded that in China under frequent serious air pollution, gender plays little role on the effect of SWB under different air pollution conditions. However, in the UK the overall air quality is much higher than China, females show more sensitivity than males in SWB with air quality changes.

## Influencing from exposure time on SWB under air pollution between China and UK

Supplementary Figure 10 shows the effect of the average daily outdoor exposure time on people’s SWB with air pollution changes for China and UK. Supplementary Figure10a and Supplementary Figure10b shows the comparisons between these 2 countries among 2 groups. Supplementary Figure10a refers to people averagely spending 2 hours or less outdoors daily and Supplementary Figure10b refers to people spending more than 2 hours outdoors each day. Both sub figures a and b show that Chinese are more sensitive than British in SWB with air pollution changes and they are happier with fairly good air condition and numb earlier when serious air pollution occurs. Interestingly, the two sub figures also show that the gaps between red and blue lines in Supplementary Figure10a are bigger than the gaps in Supplementary Figure10b. This means that in the group which spends less outdoor time, like 2 hours or less each day, people’s SWB difference between Chinese and British is more obvious than in the group which spends more outdoor time. This may be due to the fact that in serious air pollution area people who have less opportunities to spend time outside care more about the air quality when they are out. They are eager to enjoy the limited outdoor time they have each day. Supplementary Figure10c and Supplementary Figure10d shows the average daily exposure time to outdoor air influence on SWB for difference countries. It can be found in Supplementary Figure10c, that people spending less outdoor time are more sensitive under different air conditions in China. They are more care about the air quality. This is probably because serious air pollution occurs more frequently in china and people who have only limited outdoor time daily will desire good air quality more strongly. However, in Supplementary Figure10d it indicates that British people who spend longer outdoor time daily are more sensitive in SWB to air pollution than those who are spending 2 hours or less each day. This could be due to the fact that people living in the UK have been used to excellent air quality. It’s very difficult for people to accept spending lots of time outdoors in air pollution conditions.

## Influencing from knowledge impact on SWB under air pollution between China and UK

Supplementary Figure 11 shows the comparison of people’s SWB under various air pollution between Chinese and British with different knowledge of the harmfulness of air pollution. Supplementary Figure11a provides the comparisons between the 2 countries for people believing that the harmfulness of air pollution is more than smoking or similar to smoking and in Supplementary Figure11b the comparison between China and the UK is for people who believe that air pollution is less harmful than smoking or even has no effect on health. Both sub figures show that Chinese have higher SWB than British people when air pollution is very low and very high. Chinese are more sensitive to perfect air conditions and numb earlier in hazard air conditions. Interestingly, the trend is more obvious for people believe that air pollution is less harmful than smoking or has no effect on health. This could be due to that when people do not believe air pollution affects health, the experience of living in serious air pollution - which Chinese have - is displayed more strongly. For those British with the knowledge that air pollution is more harmful than - or similar to - smoking, the gap between the 2 countries is shrinking. In Supplementary Figure11c and Supplementary Figure11d, it can be found in both Chinese and British that people who believe air pollution is more harmful than, or similar to, smoking are more sensitive to perfect air condition and numb earlier in hazard air condition than people who believe air pollution is less harmful than smoking or even has no effect on health. This trend is more obvious in Chinese, which indicates that experience of living in more serious air pollution does makes people more sensitive to air pollution changes.

# Supplementary References

1 Engeldrum, P. G. *Psychometric scaling a toolkit imaging systems development*. 1 edn, (Imcotek Press, 2000).

2 Torgerson, W. S. in *Consumer Behaviour* (ed L. H. Clark) 92-93 (New York University Press, 1954).

3 Torgerson, W. S. *Theory and methods of scaling*. (John Wiley, 1958).
